# Design, Sustainable Synthesis and Biological Evaluation of a Novel Dual α2A/5-HT7 Receptor Antagonist with Antidepressant-Like Properties

**DOI:** 10.3390/molecules26133828

**Published:** 2021-06-23

**Authors:** Vittorio Canale, Magdalena Kotańska, Anna Dziubina, Matylda Stefaniak, Agata Siwek, Gabriela Starowicz, Krzysztof Marciniec, Patryk Kasza, Grzegorz Satała, Beata Duszyńska, Xavier Bantreil, Frédéric Lamaty, Marek Bednarski, Jacek Sapa, Paweł Zajdel

**Affiliations:** 1 Department of Organic Chemistry, Faculty of Pharmacy, Jagiellonian University Medical College, 30-688 Kraków, Poland; vittorio.canale@uj.edu.pl (V.C.); matylda.stefaniak@uj.edu.pl (M.S.); patryk.kasza@uj.edu.pl (P.K.); 2 Department of Pharmacodynamics, Faculty of Pharmacy, Jagiellonian University Medical College, 30-688 Kraków, Poland; magda.dudek@uj.edu.pl (M.K.); anna.dziubina@uj.edu.pl (A.D.); marek.bednarski@uj.edu.pl (M.B.); jacek.sapa@uj.edu.pl (J.S.); 3 Department of Pharmacobiology, Faculty of Pharmacy, Jagiellonian University Medical College, 30-688 Kraków, Poland; agata.siwek@uj.edu.pl (A.S.); gabriela.starowicz@gmail.com (G.S.); 4 Department of Organic Chemistry, Medical University of Silesia, 41-200 Sosnowiec, Poland; kmarciniec@sum.edu.pl; 5 Department of Medicinal Chemistry, Maj Institute of Pharmacology, Polish Academy of Sciences, 31-343 Kraków, Poland; satala@if-pan.krakow.pl (G.S.); duszyn@if-pan.krakow.pl (B.D.); 6 IBMM, Université de Montpellier, CNRS, ENSCM, 34095 Montpellier, France; xavier.bantreil@umontpellier.fr (X.B.); frederic.lamaty@umontpellier.fr (F.L.)

**Keywords:** α_2_ adrenoceptor antagonist, 5-HT_7_ receptor antagonist, medicinal mechanochemistry, depression, forced swim test

## Abstract

The complex pathophysiology of depression, together with the limits of currently available antidepressants, has resulted in the continuous quest for alternative therapeutic strategies. Numerous findings suggest that pharmacological blockade of α_2_-adrenoceptor might be beneficial for the treatment of depressive symptoms by increasing both norepinephrine and serotonin levels in certain brain areas. Moreover, the antidepressant properties of 5-HT_7_ receptor antagonists have been widely demonstrated in a large set of animal models. Considering the potential therapeutic advantages in targeting both α_2_-adrenoceptors and 5-HT_7_ receptors, we designed a small series of arylsulfonamide derivatives of (dihydrobenzofuranoxy)ethyl piperidines as dually active ligands. Following green chemistry principles, the designed compounds were synthesized entirely using a sustainable mechanochemical approach. The identified compound **8** behaved as a potent α_2A_/5-HT_7_ receptor antagonist and displayed moderate-to-high selectivity over α_1_-adrenoceptor subtypes and selected serotonin and dopaminergic receptors. Finally, compound **8** improved performance of mice in the forced swim test, displaying similar potency to the reference drug mirtazapine.

## 1. Introduction

Depressive disorder is a common and disabling illness characterized by the presence of behavioral and emotional symptoms (i.e., sleep disturbances, low self-esteem, sadness as well as suicidal ideation) [1]. Although different pharmacotherapeutic options are available (e.g., selective serotonin reuptake inhibitors SSRIs–; serotonin/noradrenaline reuptake inhibitors SNRIs; monoamine receptors modulators), the treatment of depressive disorders is still limited. Currently available antidepressants display a delay of therapeutic action, which lasts up to a few weeks in some patients after numerous antidepressant drugs, and numerous unacceptable side effects [2].

The α_2_-adrenoceptor (α_2_-AR) is a member of class A of G-protein coupled receptors (GPCRs) canonically associated with heterotrimeric Gi/o subtypes. Its activation leads to inhibition of adenylyl cyclase and voltage-gated calcium channels [3]. Among the identified α_2_-ARs, α_2A_-AR subtype is the predominant isoform (90% of all α_2_-AR) and represents the primary modulators of monoaminergic neurotransmission in CNS [4]. In particular, the high expression of α_2A_-AR in the hippocampus and cortico-limbic structures together with its involvement in serotonin release [5,6], asserts its pivotal role in cognition, memory, and mood disorders [7].

The 5-HT_7_ receptor (5-HT_7_R) represents the latest addition to a subfamily of serotonin GPCRs [8]. All isolated 5-HT_7_R isoforms are positively coupled to adenylate cyclase via activation of the Gαs subunit with consequent increasing of intracellular cAMP levels [9]. The distribution of 5-HT_7_R in specific CNS regions (i.e., the hippocampus and prefrontal cortex) suggests its implications in the control of rapid eye movement sleep, learning, and memory as well as in pathological processes such as affective disorders, neurodegenerative diseases, and cognitive decline [10,11].

A back-drop of evidence has demonstrated the potential of genetic and pharmacological blockade of α_2_-AR and 5-HT_7_R in several preclinical models of depression [12,13,14,15]. These findings are further supported by the fact that augmentation of the SSRIs and NRSIs with α_2_-AR or 5-HT_7_R antagonists increases their efficiency of monoamine reuptake inhibitors [16,17,18]. Of note, the antidepressant effects of mianserin and mirtazapine, which display high-to-moderate affinity for several monoaminergic receptors and transporters, is mainly attributed to their antagonism at presynaptic α_2A_-AR [19,20].

**Figure 1:**
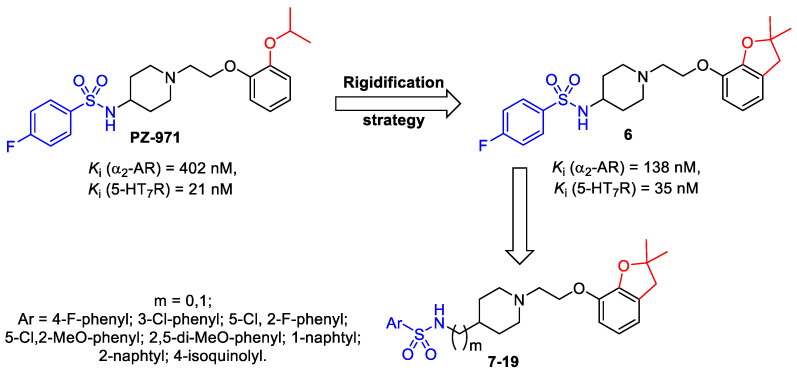
Design strategy for the arylsulfonamide derivatives of (aryloxy)ethyl piperidines **6**–**19**.

Recent studies in a group of arylsulfonamide derivatives of aryloxyalkylpiperidine [21] identified compound I as a potent 5-HT_7_R ligand with affinity for α_2_-AR in a submicromolar range (Figure 1). Further replacement of the flexible isopropoxy moiety with the rigid 2,2-dimethyl-2,3-dihydrofurane moiety increased the affinity for α_2_-AR, providing the moderate α_2_-AR ligand **6** (Figure 1). At the same time, this modification maintained high affinity for 5-HT_7_R. These findings prompted us to design novel dual acting compounds which behave as α_2_-AR and 5-HT_7_R antagonists.

Here, we present a medicinal mechanochemistry approach to the generation of a focused library of arylsulfonamides of (aryloxyethyl)piperidines (Figure 1). The impact of the applied modifications on the affinity for α_2_-AR and 5-HT_7_R was first assessed in vitro in radioligand binding studies. The selectivity of the most promising derivatives over structurally related off-target GPCRs (α_1_-AR, 5-HT_1A_R, 5-HT_2A_R, 5-HT_6_R and D_2_R) was then investigated, followed by a determination of their antagonistic properties at α_2A_-AR and 5-HT_7_R in cellular assays. Finally, the selected derivatives improved performance of mice in the forced swim test.

## 2. Results and Discussion

### 2.1. Chemistry

**Scheme 1:**
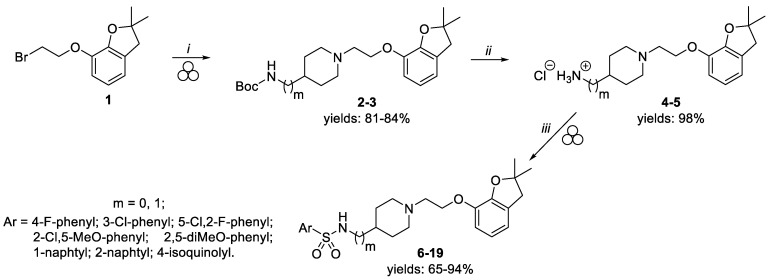
Mechanochemical synthesis of final compounds **6**–**19**. Reagents and conditions: (*i*) vbm 30 Hz, ϕ_ball_ = 1.5 cm, total mass of reagents = 500 mg, 35 mL PTFE jar, alkylating agent (1 eq) Boc-protected alicyclic diamine (1.2 eq), K_2_CO_3_ (3 eq), KI (0.5 eq), 210 min; (*ii*) HCl_gas_, 2 h; (*iii*) vbm 30 Hz, ϕ_ball_ = 1.5 cm, total mass of reagents = 125 mg, 10 mL PTFE jar, primary amine (1 eq), arylsulfonyl chloride (1 eq), K_2_CO_3_ (2 eq), 1–10 min.

In the last decade, mechanochemistry, and in particular medicinal mechanochemistry [22,23], has been recognized as an innovative technique for the generation of various organic compounds as well as producing pharmaceutically relevant fragments and functionalities [24,25,26]. Taking into account the benefits of employing a solid-state approach over classic in-batch procedures (i.e., increased reaction yield, reduction of time, decreased use of organic solvents) [27,28], we developed a mechanochemical approach for the synthesis of the designed derivatives **6**–**19** (Scheme 1). Initially, alkylation of Boc-protected 4-aminopiperidine with the commercially available 7-(2-bromoethoxy)-2,2-dimethyl-2,3-dihydrobenzofuran **1** was carried out in a 10 mL PTFE jar with a 1.5 cm diameter stainless steel ball using a Retsch vibratory ball mill (vbm) operating at 30 Hz. The use of a slight excess of amine, K_2_CO_3_ as base, in the presence of KI, allowed us to achieve high conversion rates for intermediate **2** after a milling time of 140 min (for more details see Table S1). Further scale-up optimization was required to translate reaction conditions to a 35 mL PTFE jar (see Table S1). Increasing the loading of base (from 2 to 3 eq) together with elongation of reaction time (from 140 to 210 min) enabled us to reach a 97% conversion of substrates. After an acidic extraction at pH 3.5 to remove unreacted Boc-protected alicyclic amine, intermediate **2** was isolated with a high 95% purity and 84% yield. Having identified the optimal reaction conditions, the same mechanochemical protocol was applied for the alkylation of Boc protected 4-aminomethylpiperidine to obtain intermediate **3** (96% purity) in a satisfactory yield (81%) (see Table S2). In the next step, primary amine derivatives **4** and **5**, obtained upon treatment of Boc-derivative **2** and **3** with gaseous HCl [29,30], reacted in a ball mill with different substituted arylsulfonyl chlorides to generate the designed sulfonamide derivatives.

Hence, the final compounds **6**–**19** were obtained by milling equimolar amounts of starting materials in the presence of K_2_CO_3_ in moderate-to-high yields (65–94%). According to our previously reported findings on sulfonamide bond formation in the solid-state [29], sulfonylation of the primary amine function was significantly influenced by the nature of the substituent on the phenyl ring of the sulfonyl chloride. Regardless of the type of central amine core, the presence of 4-F and 3-Cl substituents enabled the formation of compounds **6**, **7**, **14** and **15** with high conversion rates in a relatively shorter time (1 min) than all other tested analogs (see Table S3). The introduction of a second substituent at the 3-chlorophenyl moiety in both subsets (5-Cl, 2-F and 5-Cl, 2-MeO) required longer milling times to guarantee similar conversion rates for the generation of derivatives **8**–**10** and **16**–**17**. Notably, compounds **11**, **12** and **18** bearing 1-naphtyl and 2-naphtyl moieties displayed the lowest conversion rates amongst the series (<70%) after 10 min of reaction. Prolongation of the milling time for these derivatives did not increase the formation of desired products while causing degradation of substrates, which was not detected in the solution. In contrast, milling isoquinolyl-4-sulfonyl chloride and primary amines **4** and **5** for 5 min furnished final compounds **13** and **19** with the highest conversion rate amongst the series (90 and 99%, respectively).

### 2.2. In Vitro Pharmacology

All synthesized compounds were evaluated in ^3^[H]clonidine and ^3^[H]5-CT binding experiments for their affinity toward α_2_-AR and 5-HT_7_R, respectively (Table 1). The tested compounds showed high-to-low affinity for α_2_-AR (*K*_i_ = 80–1194 nM) and for 5-HT_7_R (*K*_i_ = 30–727 nM). Structure–activity relationship (SAR) studies were firstly focused on the impact of the central amine core on the affinity for both biological targets. Compounds with a 4-aminopiperidine scaffold displayed a higher affinity for both α_2_-AR and 5-HT_7_R, than their 4-aminomethylpiperidine analogs (**6** vs. **14**, **9** vs. **17** and **11** vs. **18**).

Next, the impact of the kind of substituents at the arylsulfonamide moiety was analyzed. Based on our data, reporting on the impact of monosubstituted benzenesulfonyl moiety on the affinity for α-AR and 5-HT_7_R, the analysis was limited to the 3-Cl substitution pattern [31,32]. Although replacement of the 4-F substituent present in the pilot compound **6** with the 3-Cl one (compound **7**) decreased the affinity for α_2_-AR 5-fold (*K*_i_ = 138 and 649, respectively), this modification was tolerated for interaction with the 5-HT_7_R. Regardless of the kind of central amine core, introducing a fluorine atom in the *meta* position to the 3-Cl-phenylsulfonyl moiety (i.e., 5-Cl, 2-F substitution pattern) improved the affinity of compounds **7** and **15** for both biological targets. In contrast to our previous findings [33], the replacement of one or both halogens with the electron-donating methoxy substituent decreased the affinity for α_2_-AR and 5-HT_7_R ligands (**8** vs. **9** and **10** and **16** vs. **17**).

The bicyclic 1-naphtyhyl and 2-naphthyl moiety displayed no significant improvement over the substituted phenyl ring. An exception was observed when the naphthyl substituent was replaced with the 4-isoquinolyl fragment (**11** and **12**). In line with our previously reported studies demonstrating the preferential α-position of the azinylsulfonyl moiety for interaction with biological targets [36,37,38], this modification highly increased the affinity of compound **13** for α_2_-AR up to four-fold, without drastically reducing activity at the 5-HT_7_R sites (*K*_i_ = 80 and 91 nM for α_2_-AR and 5-HT_7_R, respectively).

Compounds **6**, **8** and **13**, which displayed the most balanced activity towards α_2_-AR and 5-HT_7_R among the series, were further evaluated in vitro for their selectivity over structurally-closed monoaminergic receptors, to assess the risk of evoking CNS or cardiovascular side effects (i.e., hallucinations, body temperature, Parkinsonian-like effects, hypotension).

The tested compounds **6**, **8** and **13** displayed high selectivity over adrenergic α_1_-AR, serotonin 5-HT_2A_R and 5-HT_6_R while showing moderate affinity and selectivity for 5-HT_1A_R and D_2_R subtypes (*K*_i_ = 221–388 nM) (Table 2).

Considering the high and specific distribution of α_2A_-AR in CNS [5], and its engagement in controlling noradrenaline/serotonin release in the hippocampus and the corticolimbic structures involved in affective, cognitive and memory processes [39], targeting α_2A_-AR subtype might provide more beneficial therapeutic effects than other isolated α_2_-AR isoforms. Thus, the functional activity of **6**, **8** and **13** at α_2A_-AR and selectivity over α_2B_-AR subtypes were assessed in fluorescence-based cellular assays (Table 3) [40]. The evaluated compounds were classified as potent α_2A_-AR antagonists (*K*_b_ = 12–40 nM). Although the observed potencies were lower than those of reference α_2_-AR antagonist yohimbine, compounds **6** and **8** displayed higher functional selectivity over α_2B_-AR subtype (up to seven- and four-fold, respectively). In contrast, 4-isoquinolyl derivatives **13** did not show any preference for the tested α_2_-AR subtype.

Next, the antagonistic properties of **6**, **8** and **13** at 5-HT_7_R were confirmed in HEK-293 cells, which stably over-express the 5-HT_7_R (Table 3). All tested derivatives inhibited the cAMP production promoted by the administration of the agonist 5-CT, thus behaving as potent antagonists in this cellular setting. To exclude pharmacological effects associated with interaction with 5-HT_1A_R, further functional profiling of **6**, **8** and **13** was performed at Eurofins (Eurofins Scientific, France), revealing low agonist activity (EC_50_ > 4 μM) and no antagonist property at 1 µM in cAMP-based assays.

### 2.3. In Vivo Pharmacology

In view of the findings that the modulation of noradrenergic/serotonin transmissions by targeting α_2_-AR and the blockade of 5-HT_7_R are involved in behavioral changes responsible for antidepressant-like effects observed in preclinical models [7,10,41], selected compounds **6**, **8** and **13** were assessed for their potential antidepressant properties in the forced swim test using Albino Swiss mice. The clinically used antidepressant mirtazapine was tested as reference. Although mirtazapine displays high-to-moderate affinity for 5-HT_2A/2C_, 5-HT_3_ and5-HT_7_Rs, its antagonism at presynaptic α_2A_-AR, which enhances noradrenaline and serotonin release, is mainly related to the observed in vivo antidepressant effect [42].

**Figure 2:**
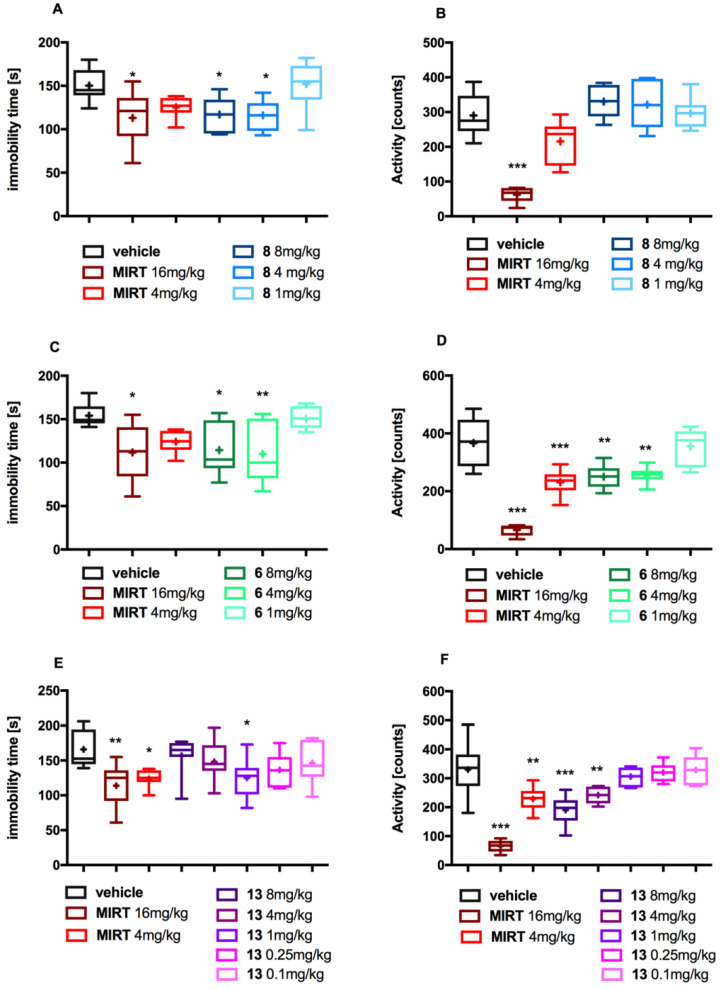
The effect of the tested compounds **6**, **8** and **13** and mirtazapine on the immobility of mice in FST (**A**,**C**,**E**) and on locomotor activity (**B**,**D**,**F**). All compounds or vehicle were administered 30 min (*ip*) before the test. MIRT—mirtazapine. The results are presented as box plots showing the following data: mean (‘+’), median (horizontal line), upper and lower quartile (the width of the box shows interquartile range), upper and lower extreme (whiskers). Statistical analysis: Shapiro–Wilk test for normality and one-way ANOVA (Dunnett post hoc) * *p <* 0.05, ** *p <* 0.01, *** *p <* 0.001 vs. control group (vehicle); *n*= 6–8 mice per group.

All tested compounds (administered at dose range of 1–8 mg/kg, *ip*) shortened the immobility of the mice by about 25% in comparison to control, thus exerting antidepressant-like effects in the FST (F(5,36) = 4.259, *p* = 0.0038; F(5,33) = 4.521, *p* = 0.003; F(7,49) = 3.209, *p* = 0.007; respectively for compounds: 8, 6, 13). In FST, the data sets for the experiments showed normal distribution (α = 0.05). Results are shown at Figure 2A,C,E. The effects were similar to those of mirtazapine displayed at the active dose of 16 mg/kg, however the antidepressant effects of **6**, **8** and **13** occurred at lower doses (8, 4 and 1 mg/kg, respectively).

Although sedation may be perceived as being therapeutically beneficial in certain stress-related mood disorders [43], it represents an unacceptable side effect, mainly related to some antidepressants. Indeed, about 20% of patients treated with mirtazapine for their depression or anxiety report sedation as a side effect [20].

To evaluate potential sedative activity at the doses used in the behavioral experiments, the influence of tested compounds on the spontaneous locomotor activity of mice was assessed. The data sets for the activity showed normal distribution (α = 0.05). Among the studied compounds, only **8** produced an antidepressant-like effect with no influence on the spontaneous locomotor activity of mice (ANOVA, F(5,33) = 21.98, *p* < 0.001) (Figure 2B). Compound **6** (4, 8 mg/kg) and mirtazapine (16 mg/kg) tested in doses effective in the FST induced sedation (ANOVA, F(5,32) = 25.99, *p* < 0.0001) (Figure 2D). Despite the fact that compound **13** showed antidepressant-like properties at the lowest dose (1 mg/kg) without affecting locomotor activity, it caused sedation at higher doses (ANOVA, F(7,44) = 20.4, *p* < 0.001) (Figure 2E). This may partially explain the lack of pharmacological effect of compound **13** at the doses of 4 and 8 mg/kg in the FST.

## 3. Materials and Methods

### 3.1. Chemistry

#### 3.1.1. General Chemical Methods

All commercially available reagents were of the highest purity (from Sigma-Aldrich, Fluorochem, AlfaAesar). The milling treatments were carried out in a vibratory ball-mill Retsch MM400 operated at 30 Hz. The milling load was defined as the sum of the mass of the reactants per free volume in the jar and was equal to 15 mg/mL. All reactions using the vibratory ball mill were performed under air.

^1^H and ^13^C NMR spectra were recorded on a JEOL JNM-ECZR500 RS1 (ECZR version) at 500 and 126 MHz, respectively, and were reported in ppm using deuterated solvent for calibration (CDCl_3_). The *J* values were reported in hertz (Hz), and the splitting patterns were designated as follows: br s. (broad singlet), br d. (broad doublet), s (singlet), d (doublet), t (triplet), q (quartet), dd (doublet of doublets), dt (doublet of triplets), td (triplet of doublets), tt (triplet of triplets), ddd (doublet of doublet of doublets), dq (doublet of quartets), dddd (doublet of doublet of doublet of doublets), m (multiplet).

Mass spectra were recorded on a UPLCMS/MS system consisting of a Waters ACQUITY UPLC (Waters Corporation, Milford, MA, USA) coupled to a Waters TQD mass spectrometer (electrospray ionization mode ESI-tandem quadrupole). Chromatographic separations were carried out using the Acquity UPLC BEH (bridged ethyl hybrid) C18 column; 2.1 mm × 100 mm, and 1.7 μm particle size, equipped with Acquity UPLC BEH C18 Van Guard precolumn; 2.1 mm × 5 mm, and 1.7 μm particle size. The column was maintained at 40 °C and eluted under gradient conditions from 95% to 0% of eluent A over 10 min, at a flow rate of 0.3 mL min^−1^. Eluent A: water/formic acid (0.1%, *v*/*v*), Eluent B: acetonitrile/formic acid (0.1%, *v*/*v*).

Melting points (mp) were determined with a Büchi apparatus and are uncorrected.

Elemental analyses for C, H, N and S were carried out using the elemental Vario EL III Elemental Analyser (Hanau, Germany). All values are given as percentages and were within ±0.4% of the calculated values.

#### 3.1.2. Alkylation of Boc-Protected 4-Aminopiperidine in Ball Mill (Procedure A)

Commercially available bromine derivative **1** (38.9 mg, 0.144 mmol, 1 eq) and Boc-protected alicyclic amine (34.5 mg, 0.172 mmol, 1.2 eq) were introduced in a 10 mL PTFE jar (milling load 15 mg/mL) with one stainless steel ball (ϕ_ball_ = 1.5 cm), followed by the addition of previously ground K_2_CO_3_ (39.7 mg, 0.287 mmol, 2 eq) and KI (14.3 mg, 0.086 mmol, 0.5 eq). The reaction was carried out for 140 min. Then, the product was solubilized in CH_2_Cl_2_ (10 mL), and the organic phase was washed with KHSO_4_ aqueous solution at pH = 3.5 (3 × 5 mL) and saturated NaCl solution (1 × 5 mL), dried over Na_2_SO_4_, and finally filtered and concentrated under reduced pressure.

#### 3.1.3. Alkylation of Boc-Protected 4-Aminopiperidine and 4-Aminomethylpiperidine in Ball Mill (Procedure B)

Commercially available bromine derivative **1** (1 eq) and Boc-protected alicyclic diamine (1.2 eq) were introduced in two 35 mL PTFE jars (milling load 15 mg/mL) with one stainless steel ball (ϕ_ball_ = 1.5 cm), followed by the addition of previously ground K_2_CO_3_ (3 eq) and KI (0.5 eq). The reaction was carried out for 210 min at rt. Then, the product was solubilized in CH_2_Cl_2_ (25 mL), and the organic phase was washed with KHSO_4_ aqueous solution at pH = 3.5 (3 × 10 mL) and saturated NaCl solution (1 × 10 mL), dried over Na_2_SO_4_, and finally filtered and concentrated under reduced pressure. To obtain the desired amount of product, the reaction was carried out twice (4 × 35 mL).

##### Tert-butyl {1-[2-(2,2-Dimethyl-2,3-dihydrobenzofuran-7-yloxy)ethyl]piperidin-4-yl}carbamate (**2**)

General procedure B was followed with bromine derivative **1** (134.4 mg, 0.495 mmol, 1 eq), Boc-protected 4-aminopiperidine (119.1 mg, 0.595 mmol, 1.2 eq), previously ground K_2_CO_3_ (205.4 mg, 1.486 mmol, 3 eq), and KI (49.4 mg, 0.297 mmol, 0.5 eq) to afford intermediate **3** as a white powder (164 mg and 84% yield).

C_22_H_34_N_2_O_4_, MW: 390.52, Monoisotopic Mass: 390.25. UPLC/MS purity 96%, *t*_R_ = 4.99 min; [M+H]^+^ 391.4. ^1^H NMR (500 MHz, CDCl_3_) δ 6.78–6.73 (m, 3H), 4.45 (s, 1H), 4.24–4.17 (m, 2H), 3.48 (d, *J* = 7.1 Hz, 1H), 3.01 (t, *J* = 1.6 Hz, 3H), 3.00–2.95 (m, 2H), 2.88–2.82 (m, 2H), 2.28 (d, *J* = 10.1 Hz, 2H), 1.95 (d, *J* = 11.5 Hz, 2H), 1.50 (s, 1H), 1.48 (s, 6H), 1.43 (s, 9H). ^13^C NMR (126 MHz, CDCl_3_) δ 155.3, 147.9, 143.5, 128.7, 120.5, 118.1, 113.6, 87.5, 79.4, 66.7, 57.1, 52.9, 43.4, 32.3, 28.5, 28.4. Mp for C_22_H_34_N_2_O_4_ 118.1–119.8 °C.

##### Tert-butyl ({1-[2-(2,2-Dimethyl-2,3-dihydrobenzofuran-7-yloxy)ethyl]piperidin-4-yl}methyl)carbamate (**3**)

General procedure B was followed with bromine derivative **1** (132.1 mg, 0.487 mmol, 1 eq), Boc-protected 4-aminomethylpiperidine (125.4 mg, 0.585 mmol, 1.2 eq), previously ground K_2_CO_3_ (202.1 mg, 1.462 mmol, 3 eq), and KI (48.5 mg, 0.292 mmol, 0.5 eq) to afford intermediate **4** as a white powder (154 mg and 81% yield).

C_23_H_36_N_2_O_4_, MW: 404.55 Monoisotopic Mass: 404.27. UPLC/MS: purity 96%, *t*_R_ = 5.17 min; [M+H]^+^ 405.4. ^1^H NMR (500 MHz, CDCl_3_) δ 6.91–6.60 (m, 3H), 4.65 (dd, *J* = 7.2, 6.5 Hz; 1H), 4.30–4.22 (m, 2H), 3.16–3.10 (m, 2H), 3.04–2.98 (m, 4H), 2.95–2.88 (m, 2H), 2.29–2.22 (m, 2H), 1.75–1.71 (m, 2H), 1.56–1.54 (m, 1H), 1.48 (s, 6H), 1.45 (d, *J* = 3.8 Hz, 1H), 1.43 (s, 9H). ^13^C NMR (126 MHz, CDCl_3_) δ 156.2, 148.0, 128.7, 120.5, 118.3, 113.8, 87.5, 79.4, 66.4, 57.2, 53.8, 46.0, 43.4, 31.1, 28.5, 28.4. Mp for C_23_H_36_N_2_O_4_: 205.0–208.0 °C.

#### 3.1.4. General Procedure for the Deprotection of Boc Function in Solid State (Procedure C)

Intermediate **2** or **3** was submitted to HCl_gas_ for 2 h at rt to afford the primary amine **4** or **5** as white hydrochloride salts, according to previously reported procedures [29].

##### 1-{2-[(2,2-Dimethyl-2,3-dihydrobenzofuran-7-yl)oxy]ethyl}piperidin-4-amine hydrochloride (**4**)

General procedure C was followed with derivative **2** (600 mg, 1.537 mmol, 1 eq) to afford intermediate **4** as a yellow powder (492 mg and 98% yield).

C_17_H_26_N_2_O_2·_HCl, MW: 326.86, Monoisotopic Mass: 290.20. UPLC/MS: purity 100%, *t*_R_ = 2.61 min, [M+H]^+^ 291.3. ^1^H NMR for free base (500 MHz, CDCl_3_) δ 6.76 (dt, *J* = 8.1, 4.6 Hz, 3H), 4.18 (t, *J* = 6.4 Hz, 2H), 3.01 (s, *J* = 3.4 Hz, 2H), 2.99–2.92 (m, 2H), 2.81 (t, *J* = 6.4 Hz, 2H), 2.20–2.13 (m, 2H), 1.86 (d, *J* = 15.5 Hz, 1H), 1.51 (s, 1H), 1.49 (s, 6H), 1.46 (d, *J* = 10.5 Hz, 1H). ^13^C NMR for free base (126 MHz, CDCl_3_) δ 147.7, 143.6, 128.5, 120.5, 117.9, 113.0, 87.5, 66.5, 57.0, 52.9, 48.7, 43.4, 35.1, 28.4. Mp for C_17_H_26_N_2_O_2_ HCl: 139.5–140.3 °C.

##### (1-{2-[(2,2-Dimethyl-2,3-dihydrobenzofuran-7-yl)oxy]ethyl}piperidin-4-yl)methanamine hydrochloride (**5**)

General procedure C was followed with derivative **3** (600 mg, 1.483 mmol, 1 eq) to afford intermediate **5** as a yellow powder (505 mg and 98% yield).

C_18_H_28_N_2_O_2·_HCl, MW: 340.89 Monoisotopic Mass: 304.22. UPLC/MS: purity 100%, *t*_R_ =2.72 min, [M+H]^+^ 305.3. ^1^H NMR for free base (500 MHz, CDCl_3_) δ 6.75 (dt, *J* = 9.6, 4.3 Hz, 3H), 4.18 (t*, J* = 6.5 Hz, 3H), 3.01 (d, *J* = 8.9 Hz, 2H), 2.80 (t, *J* = 6.4 Hz, 2H), 2.60 (d, *J* = 6.0 Hz, 2H), 2.52 (s, 1H), 2.08 (t, *J* = 11.5 Hz, 2H) 1.73 (d, *J* = 12.3 Hz, 2H), 1.49 (s, 6H), 1.30–1.23 (m, 4H). ^13^C NMR for free base (126 MHz, CDCl_3_) δ 147.8, 143.7, 128.5, 120.4, 117.8, 113.2, 87.5, 66.7, 57.4, 54.2, 47.9, 43.4, 38.7, 29.9, 28.4. Mp for C_18_H_28_N_2_O_2_ HCl: 110.9–112.4 °C.

#### 3.1.5. Sulfonylation of Primary Amine (Procedure D) for the Preparation of Final Compounds (**6**–**19**)

Intermediate **4** or **5** (1 eq), selected arylsulfonyl chloride (1 eq), and previously ground K_2_CO_3_ (2 eq) were introduced in a 10 mL PTFE jar (milling load 15 mg/mL) with one stainless steel ball (ϕ_ball_ = 1.5 cm). The reaction was carried out for 1−10 min at rt. Then, the crude mixture was solubilized in AcOEt (10 mL), and the organic phase was washed with KHSO_4_ aqueous solution at pH = 3.5 (3 × 5 mL), saturated NaCl solution (1 × 5 mL), dried over Na_2_SO_4_, and finally filtered and concentrated under a vacuum. To obtain the desired amount of product, the reaction was carried out twice (2 × 10 mL).

#### 3.1.6. Sulfonylation of Primary Amine (Procedure E) for the High-Scale Preparation of Selected Final Compounds (**6**, **8** and **13**)

Intermediate **4** or **5** (1 eq), selected arylsulfonyl chloride (1 eq), and previously ground K_2_CO_3_ (2 eq) were introduced in a 35 mL PTFE jar (milling load 15 mg/mL) with one stainless steel ball (ϕ_ball_ = 1.5 cm). The reaction was carried out for 1−5 min at rt. Then, the crude mixture was solubilized in AcOEt (15 mL), and the organic phase was washed with KHSO_4_ aqueous solution at pH = 3.5 (3 × 10 mL), saturated NaCl solution (1 × 10 mL), dried over Na_2_SO_4_, and finally filtered and concentrated under a vacuum. To obtain the desired amount of product, the reaction was carried out twice (2 × 15 mL).

#### 3.1.7. Characterization of Final Compounds

##### 4-Fluoro-*N*-{1-[2-(2,2-dimethyl-2,3-dihydrobenzofuran-7-yloxy)ethyl]piperidin-4-yl} benzenesulfonamide (**6**)

General Procedure D was followed with primary amine **4** (51.27 mg, 0.157 mmol, 1 eq), 4-fluorobenzenesulfonyl chloride (30.4 mg, 0.157 mmol, 1 eq), and previously ground K_2_CO_3_ (43.3 mg, 0.314 mmol, 2 eq) to afford final compound **6** as a white solid, 62.6 mg (89% isolated yield). For the in vivo pharmacological studies, general procedure E was followed with primary amine **4** (205.1 mg, 0.627 mmol, 1 eq), 4-fluoro-benzenesulfonyl chloride (121.7 mg, 0.627 mmol, 1 eq), and previously ground K_2_CO_3_ (173.2 mg, 1.255 mmol, 2 eq) to afford final compound **6** as a white solid, 250.5 mg (85% isolated yield). Compound **6** was converted to the hydrochloride salt according to procedure C.

C_23_H_29_FN_2_O_4_S, MW: 448.55, Monoisotopic Mass: 448.18. UPLC/MS purity 99%, t_R_ = 4.99 min, [M+H]^+^ 449.3. ^1^H NMR (500 MHz, CDCl_3_): δ 7.98 (s, 2H), 7.15 (d, *J* = 6.5 Hz, 2H), 6.86–6.58 (m, 3H), 4.49 (d, *J* = 30.1 Hz, 2H), 3.65 (d, *J* = 57.7 Hz, 2H), 3.40 (d, *J* = 70.9 Hz, 3H), 3.00 (t, *J* = 9.1 Hz, 3H), 2.83–2.60 (m, 2H), 2.34 (s, 2H), 2.08–1.87 (m, 2H), 1.45 (s, 2H), 1.44 (s, 4H). ^13^C NMR (126 MHz, CDCl_3_) δ 166.1(^1^*J*_C-F_ = 254.5 Hz), 164.0, 148.0, 141.8, 137.4 (^4^*J*_C-F_ = 126.8 Hz), 129.9, 129.8 (^3^*J*_C-F_ = 9.0 Hz), 129.3 (^3^*J*_C-F_ = 8.8 Hz), 120.9, 119.7, 116.6, 116.4, 115.2, 88.1 (^2^*J*_C-F_ = 22.4 Hz), 64.4, 55.8, 52.4, 48.6, 48.4, 45.1, 43.1, 29.9, 28.4, 27.8. Mp for C_23_H_29_FN_2_O_4_S: 117.5–119.8 °C. Mp for C_23_H_29_FN_2_O_4_S HCl: 165.0–166.6 °C. Anal. calcd for C_23_H_29_FN_2_O_4_S HCl: C: 56.96, H: 6.23, N: 5.78, S: 6.71; Found C: 57.17, H: 6.32, N: 5.74, S: 6.39.

##### 3-Chloro-*N*-{1-[2-(2,2-dimethyl-2,3-dihydrobenzofuran-7-yloxy)ethyl]piperidin-4-yl}benzenesulfonamide (**7**)

General Procedure D was followed with primary amine **4** (50.2 mg, 0.154 mmol, 1 eq), 3-chlorobenzenesulfonyl chloride (21.6 µL, 0.154 mmol, 1 eq), and previously ground K_2_CO_3_ (42.4 mg, 0.307 mmol, 2 eq) to afford final compound **7** as yellow solid, 61.3 mg (86% isolated yield). 

C_23_H_29_ClN_2_O_4_S, MW: 464.01, Monoisotopic Mass: 464.15. UPLC/MS purity 95%, t_R_ = 5.15 min, [M+H]^+^ 465.2. ^1^H NMR (500 MHz, CDCl_3_): δ 7.88 (t, *J* = 1.9 Hz, 1H), 7.77 (ddd, *J* = 7.8, 1.7, 1.0 Hz, 1H), 7.53 (ddd, *J* = 8.0, 2.1, 1.1 Hz, 1H), 7.45 (t, *J* = 7.9 Hz, 1H), 6.80–6.68 (m, 3H), 5.08–4.91 (m, 1H), 4.16 (t, *J* = 5.9 Hz, 2H), 3.22 (d, *J* = 10.2 Hz, 1H), 3.00 (s, 2H), 2.92 (d, *J* = 11.5 Hz, 2H), 2.82 (t, *J* = 6.0 Hz, 2H), 2.26 (s, 2H), 1.81 (d, *J* = 12.8 Hz, 2H), 1.65–1.52 (m, 2H), 1.47 (s, 6H). ^13^C NMR (126 MHz, CDCl_3_): δ 147.9, 143.4, 143.2, 135.4, 132.8, 130.6, 128.7, 127.2, 125.1, 120.5, 118.3, 113.6, 87.6, 66.6, 56.9, 52.3, 50.7, 43.4, 32.8, 29.8, 28.4. Mp for C_23_H_29_ClN_2_O_4_S: 118.9–120.7 °C.

##### 5-Chloro-2-fluoro-*N*-{1-[2-(2,2-dimethyl-2,3-dihydrobenzofuran-7-yloxy)ethyl]piperidin-4-yl}benzenesulfonamide (**8**)

General Procedure D was followed with primary amine **4** (49.2 mg, 0.150 mmol, 1 eq), 5-chloro-2-fluorobenzenesulfonyl chloride (34.3 mg, 0.150 mmol, 1 eq), and previously ground K_2_CO_3_ (41.5 mg, 0.300 mmol, 2 eq) to afford final compound **8** as a colorless oil, 48.7 g (68% isolated yield). For in vivo pharmacological studies, general procedure E was followed with primary amine **4** (196.7 mg, 0.602 mmol, 1 eq), 5-chloro-2-fluorobenzenesulfonyl chloride (137.2 mg, 0.602 mmol, 1 eq), and previously ground K_2_CO_3_ (166.1 mg, 1.204 mmol, 2 eq) to afford final compound **8** as a colorless oil, 194.7 mg (67% isolated yield). Compound **8** was converted to the hydrochloride salt according to procedure C.

C_23_H_28_ClFN_2_O_4_S, MW: 483.0, Monoisotopic Mass: 482.14. UPLC/MS purity 100%, t_R_ = 5.37 min, [M+H]^+^ 483.2. ^1^H NMR (500 MHz, CDCl_3_): δ 7.85 (td, *J* = 6.4, 2.6 Hz, 1H), 7.56–7.46 (m, 1H), 7.14 (t, *J* = 9.0 Hz, 1H), 6.85–6.69 (m, 3H), 4.51 (dt, *J* = 41.8, 4.3 Hz, 1H), 3.71–3.40 (m, 5H), 3.08 (d, *J* = 11.6 Hz, 1H), 3.03–2.95 (m, 2H), 2.44 (d, *J* = 13.5 Hz, 2H), 2.30 (s, 2H), 2.07–1.85 (m, 2H), 1.46 (s, 3H), 1.46 (s, 3H). ^13^C NMR (126 MHz, CDCl_3_) δ 158.3, 156.3 (^1^*J*_C-F_ = 253.9 Hz), 147.9, 143.4, 134.7, 134.6 (^3^*J*_C-F_ = 8.5 Hz), 131.0, 130.9 (^2^*J*_C-F_ = 15,2 Hz), 130.0, 130.0 (^4^*J*_C-F_ = 3.4 Hz), 129.8, 128.7, 120.5, 118.6, 118.4 (^2^*J*_C-F_ = 23.1 Hz), 118.2, 113.5, 87.5, 66.6, 56.9, 53.6, 52.3, 51.0, 43.4, 32.7, 28.4. Mp for C_23_H_28_ClFN_2_O_4_S: 170.9–173.3 °C. Mp for C_23_H_28_ClFN_2_O_4_S HCl: 189.2–191.6 °C. Anal. calculated for C_23_H_28_ClFN_2_O_4_S HCl: C: 53.18, H: 5.63, N: 5.39, S: 6.17; Found C: 53.37, H: 5.43, N: 5.54, S: 6.39.

##### 5-Chloro-2-methoxy-*N*-{1-[2-(2,2-dimethyl-2,3-dihydrobenzofuran-7-yloxy)ethyl]piperidin-4-yl}benzenesulfonamide (**9**)

General Procedure D was followed with primary amine **4** (48.5 mg, 0.148 mmol, 1 eq), 5-chloro-2-methoxybenzenesulfonyl chloride (35.6 mg, 0.148 mmol, 1 eq), and previously ground K_2_CO_3_ (41.0 mg, 0.297 mmol, 2 eq) to afford final compound **9** as a white solid, 61.7 mg (84% isolated yield).

C_24_H_31_ClN_2_O_5_S, MW: 495.03, Monoisotopic Mass: 494.16. UPLC/MS purity 95%, t_R_ = 5.37 min, [M+H]^+^ 495.3. ^1^H NMR (300 MHz, CDCl_3_): 7.89 (d, *J* = 2.7 Hz, 1H), 7.48 (dd, *J* = 8.8, 2.7 Hz, 1H), 6.96 (d, *J* = 8.8 Hz, 1H), 6.79–6.67 (m, 3H), 4.97 (d, *J* = 8.0 Hz, 1H), 4.16 (t, *J* = 6.0 Hz, 2H), 3.97 (s, 3H), 3.24 (dd, *J* = 7.9, 4.0 Hz, 1H), 3.02–2.97 (m, 2H), 2.90 (d, *J* = 10.7 Hz, 2H), 2.82 (s, 2H), 2.26 (s, 2H), 1.75 (s, 2H), 1.60–1.53 (m, 2H), 1.47 (s, 6H). ^13^C NMR (126 MHz, CDCl_3_) δ 154.7, 148.0, 143.3, 134.2, 130.5, 129.6, 128.8, 126.1, 120.5, 118.3, 113.8, 113.7, 87.5, 66.7, 56.9, 56.8, 52.3, 43.3, 32.4, 29.8, 28.4. Mp for C_24_H_31_ClN_2_O_5_S: 147.4–149.7 °C.

##### 2,5-Dimethoxy-*N*-{1-[2-(2,2-dimethyl-2,3-dihydrobenzofuran-7-yloxy)ethy]piperidin-4-yl}benzenesulfonamide (**10**)

General Procedure D was followed with primary amine **4** (48.7 mg, 0.149 mmol, 1 eq), 2,5-dimethoxybenzenesulfonyl chloride (35.2 mg, 0.149 mmol, 1 eq), and previously ground K_2_CO_3_ (41.1 mg, 0.298 mmol, 2 eq) to afford final compound **6** as a light-yellow solid, 51.1 mg (70% isolated yield). 

C_25_H_34_N_2_O_6_S, MW: 490.62, Monoisotopic Mass: 490.21. UPLC/MS purity 100%, t_R_ = 4.96 min, [M+H]^+^ 491.3. ^1^H NMR (500 MHz, CDCl_3_): δ 7.44 (d, *J* = 3.2 Hz, 1H), 7.05 (dd, *J* = 9.0, 3.1 Hz, 1H), 6.95 (d, *J* = 9.0 Hz, 1H), 6.79–6.68 (m, 3H), 4.97 (d, *J* = 7.7 Hz, 1H), 4.15 (t, *J* = 6.1 Hz, 2H), 3.92 (s, 3H), 3.81 (s, 3H), 3.24–3.15 (m, 1H), 3.00 (d, *J* = 1.0 Hz, 2H), 2.79 (d, *J* = 6.9 Hz, 2H), 2.22 (s, 2H), 1.76 (d, *J* = 14.5 Hz, 4H), 1.52 (d, *J* = 11.1 Hz, 2H), 1.46 (s, 6H). ^13^C NMR (126 MHz, CDCl_3_) δ 153.4, 150.1, 147.95, 143.4, 129.6, 128.7, 120.4, 120.3, 118.2, 114.2, 113.8, 113.7, 0 87.5, 66.8, 56.9, 56.1, 52.3, 43.3, 32.5, 28.4. Mp for C_25_H_34_N_2_O_6_S:124.9–125.8 °C.

##### 1-Naphthalene-*N*-{1-[2-(2,2-dimethyl-2,3-dihydrobenzofuran-7-yloxy)ethyl]piperidin-4-yl}sulfonamide (**11**)

General Procedure D was followed with primary amine **4** (49.2 mg, 0.151 mmol, 1 eq), 1-naphthalenesulfonyl chloride (34.2 mg, 0.151 mmol, 1 eq), and previously ground K_2_CO_3_ (41.6 mg, 0.301 mmol, 2 eq) to afford final compound **11** as a light-brown solid, 48.5 mg (67% isolated yield).

C_27_H_32_N_2_O_4_S, MW: 480.62, Monoisotopic Mass: 480.21. UPLC/MS purity 95%, t_R_ = 5.54 min, [M+H]^+^ 481.3. ^1^H NMR (500 MHz, CDCl_3_): δ 8.61 (dq, *J* = 8.7, 1.0 Hz, 1H), 8.28 (dd, *J* = 7.3, 1.3 Hz, 1H), 8.06 (dt, *J* = 8.4, 1.2 Hz, 1H), 8.00–7.91 (m, 1H), 7.66 (ddd, *J* = 8.5, 6.9, 1.4 Hz, 1H), 7.59 (ddd, *J* = 8.0, 6.9, 1.1 Hz, 1H), 7.53 (dd, *J* = 8.2, 7.3 Hz, 1H), 6.80–6.62 (m, 3H), 4.97 (s, 1H), 4.09 (t, *J* = 5.9 Hz, 2H), 3.22–3.15 (m, 1H), 2.99 (s, 2H), 2.82 (d, *J* = 11.7 Hz, 2H), 2.75 (t, *J* = 5.9 Hz, 2H), 2.15 (s, 2H), 1.64 (d, *J* = 11.8 Hz, 2H), 1.49 (d, *J* = 2.8 Hz, 2H), 1.45 (s, 6H). ^13^C NMR (126 MHz, CDCl_3_): δ 147.8, 143.4, 135.8, 134.4, 134.4, 129.5, 129.3, 128.7, 128.5, 128.2, 127.1, 124.5, 124.3, 120.5, 118.2, 113.3, 87.6, 66.2, 56.8, 52.3, 43.3, 32.5, 28.4. Mp for C_27_H_32_N_2_O_4_S: 170.0–173.7 °C.

##### 2-Naphthalene-*N*-{1-[2-(2,2-dimethyl-2,3-dihydrobenzofuran-7-yloxy)ethyl]piperidin-4-yl}sulfonamide (**12**)

General Procedure D was followed with primary amine **4** (49.2 mg, 0.151 mmol, 1 eq), 1-naphthalenesulfonyl chloride (34.2 mg, 0.151 mmol, 1 eq), and previously ground K_2_CO_3_ (41.6 mg, 0.301 mmol, 2 eq) to afford final compound **12** as a white solid, 47.1 mg (65% isolated yield).

C_27_H_32_N_2_O_4_S, MW: 480.62, Monoisotopic Mass: 480.21. UPLC/MS purity 95%, t_R_ = 5.53 min, [M+H]^+^ 481.3. ^1^H NMR (500 MHz, CDCl_3_): δ 8.40 (d, *J* = 1.9 Hz, 1H), 7.95–7.76 (m, 4H), 7.57 (dddd, *J* = 17.6, 8.2, 6.9, 1.4 Hz, 2H), 6.72–6.58 (m, 3H), 4.86 (ddt, *J* = 10.2, 2.3, 1.2 Hz, 1H), 4.07 (t, *J* = 5.9 Hz, 2H), 3.21–3.17 (m, 1H), 2.97–2.90 (m, 2H), 2.88–2.78 (m, 2H), 2.75–2.70 (m, 2H), 2.13 (d, *J* = 22.5 Hz, 2H), 1.73 (d, *J* = 12.0 Hz, 2H), 1.57–1.47 (m, 2H), 1.38 (s, 6H). ^13^C NMR (126 MHz, CDCl_3_): δ 147.9, 143.4, 138.1, 134.9, 132.3, 129.7, 129.4, 128.9, 128.7, 128.3, 128.1, 127.7, 122.4, 120.5, 118.2, 113.6, 87.5, 66.6, 56.8, 53.6, 52.3, 43.3, 28.3. Mp for C_27_H_32_N_2_O_4_S: 175.0–178.9 °C.

##### 4-Isoquinoline-*N*-{1-[2-(2,2-dimethyl-2,3-dihydrobenzofuran-7-yloxy)ethyl]piperidin-4-yl}sulfonamide (**13**)

General Procedure D was followed with primary amine **4** (49.2 mg, 0.150 mmol, 1 eq), 4-isoquinolinsulfonyl chloride (34.3 mg, 0.150 mmol, 1 eq), and previously ground K_2_CO_3_ (41.6 mg, 0.301 mmol, 2 eq) to afford final compound **13** as white solid, 59.4 mg (82% isolated yield). For in vivo pharmacological studies, general procedure E was followed with primary amine **4** (196.7 mg, 0.602 mmol, 1 eq), 4-isoquinolinsulfonyl chloride (137.0 mg, 0.602 mmol, 1 eq), and previously ground K_2_CO_3_ (166.3 mg, 1.204 mmol, 2 eq) to afford final compound **13** as a white solid, 220.3 mg (76% isolated yield). Compound **13** was converted to the hydrochloride salt according to procedure C.

C_26_H_31_N_3_O_4_S, MW: 481.61, Monoisotopic Mass: 481.2. UPLC/MS purity 99%, t_R_ = 4.59 min, [M+H]^+^ 482.3. ^1^H NMR (500 MHz, CD_3_OD) δ 9.95 (s, 1H), 9.10 (s, 1H), 8.84 (d, *J* = 8.6 Hz, 1H), 8.61 (d, *J* = 8.1 Hz, 1H), 8.35 (ddd, *J* = 8.4, 7.1, 1.1 Hz, 1H), 8.20–8.04 (m, 1H), 6.89–6.66 (m, 3H), 4.28 (t, *J* = 4.6 Hz, 2H), 3.65–3.53 (m, 3H), 3.45 (t, *J* = 4.6 Hz, 2H), 3.27 (dt, *J* = 1.6 Hz, 2H), 3.10 (s, 1H), 3.02–2.97 (m, 2H), 1.87 (dd, *J* = 69.4, 13.4 Hz, 4H), 1.42 (s, 1H), 1.41 (s, 4H). ^13^C NMR (126 MHz, CDCl_3_) δ 151.2, 147.9, 143.4, 143.3, 137.2, 133.3, 130.3, 129.0, 128.7, 125.9, 122.4, 120.4, 118.1, 113.6, 87.4, 66.7, 56.9, 53.6, 52.3, 43.3, 32.4, 28.3. Mp for C_26_H_31_N_3_O_4_S: 172.6–174.6 °C. Mp for C_26_H_31_N_3_O_4_S HCl: 205.1–206.6 °C. Anal. calcd for C_26_H_31_N_3_O_4_S HCl: C: 60.28, H: 6.23, N: 8.11, S: 6.19; Found C: 60.47, H: 6.32, N: 8.52, S: 6.29.

##### 4-Fluoro-*N*-({1-[2-(2,2-dimethyl-2,3-dihydrobenzofuran-7-yloxy)ethyl]piperidin-4-yl}methyl)benzenesulfonamide (**14**)

General Procedure D was followed with primary amine **5** (52.5 mg, 0.154 mmol, 1 eq), 4-fluorobenzenesulfonyl chloride (30.0 mg, 0.154 mmol, 1 eq), and previously ground K_2_CO_3_ (42.5 mg, 0.308 mmol, 2 eq) to afford final compound **14** as yellow solid, 64.1 mg (90% isolated yield).

C_24_H_31_FN_2_O_4_S, MW: 462.58, Monoisotopic Mass: 462.2. UPLC/MS purity 100%, *t*_R_ = 5.13 min, [M+H]^+^ 463.3. ^1^H NMR (300 MHz, CDCl_3_): δ 7.87 (dd, *J* = 8.9, 5.0 Hz, 2H), 7.17 (t, *J* = 8.6 Hz, 2H), 6.82–6.59 (m, 3H), 4.16 (s, 2H), 3.09–3.03 (m, 2H), 3.01 (d, *J* = 0.8 Hz, 2H), 2.83 (t, *J* = 5.9 Hz, 4H), 2.10 (td, *J* = 11.8, 2.5 Hz, 2H), 1.70–1.63 (m, 2H), 1.48 (s, 6H), 1.40–1.20 (m, 3H). ^13^C NMR (126 MHz, CDCl_3_) δ 167.1, 165.1 (^1^*J*_C-F_ = 254.6 Hz), 148.6, 144.4, 137.1, 137.1, 130.9, 130.8 (^3^*J*_C-F_ = 9.3 Hz), 129.5, 121.5, 119.0, 117.5, 117.3 (^2^*J*_C-F_ = 22.4 Hz), 113.9, 88.6, 66.7, 58.2, 54.5, 49.5, 44.3, 36.8, 30.1, 29.3. Mp for C_24_H_31_FN_2_O_4_S: 189.1–192.4 °C.

##### 3-Chloro-*N*-({1-[2-(2,2-dimethyl-2,3-dihydrobenzofuran-7-yloxy)ethyl]piperidin-4-yl}methyl)benzenesulfonamide (**15**)

General Procedure D was followed with primary amine **5** (51.5 mg, 0.151 mmol, 1 eq), 3-chlorobenzenesulfonyl chloride (21.3 µL, 0.151 mmol, 1 eq), and previously ground K_2_CO_3_ (41.7 mg, 0.302 mmol, 2 eq) to afford final compound **15** as a colorless oil, 60.0 mg (83% isolated yield).

C_24_H_31_ClN_2_O_4_S, MW: 479.03, Monoisotopic Mass: 478.17. UPLC/MS purity 100%, t_R_ = 5.45 min, [M+H]^+^ 479.3. ^1^H NMR (300 MHz, CDCl_3_): δ 7.85 (t, *J* = 1.9 Hz, 1H), 7.74 (dt, *J* = 7.9, 1.4 Hz, 1H), 7.53 (ddd, *J* = 8.1, 2.1, 1.1 Hz, 1H), 7.45 (t, *J* = 7.9 Hz, 1H), 6.79–6.69 (m, 3H), 4.18 (t, *J* = 6.0 Hz, 2H), 3.05 (d, *J* = 11.4 Hz, 2H), 3.01 (s, 2H), 2.85 (dq, *J* = 6.1, 2.8 Hz, 4H), 2.16–2.09 (m, 2H), 1.72–1.66 (m, 2H), 1.48 (s, 6H), 1.37–1.23 (m, 3H). ^13^C NMR (126 MHz, CDCl_3_): δ 147.5, 143.2, 141.6, 135.2, 132.6, 130.3, 128.4, 127.0, 125.0, 120.3, 117.8, 113.0, 87.3, 65.9, 56.9, 53.3, 48.4, 43.1, 35.6, 29.0, 28.1.

##### 5-Chloro-2-flluoro-*N*-({1-[2-(2,2-dimethyl-2,3-dihydrobenzofuran-7-yloxy)ethylpiperidin-4-yl}methyl)benzenesulfonamide (**16**)

General Procedure D was followed with primary amine **5** (50.4 mg, 0.148 mmol, 1 eq), 5-chloro-2-fluorobenzenesulfonyl chloride (33.7 mg, 0.148 mmol, 1 eq), and previously ground K_2_CO_3_ (40.9 mg, 0.296 mmol, 2 eq) to afford final compound **16** as a colorless oil, 49.3 mg (67% isolated yield).

C_24_H_30_ClFN_2_O_4_S, MW: 497.02, Monoisotopic Mass: 496.16. UPLC/MS purity 100%, t_R_ = 5.51 min, [M+H]^+^ 497.3. ^1^H NMR (300 MHz, CDCl_3_): δ 7.86 (dd, *J* = 6.1, 2.7 Hz, 1H), 7.51 (ddd, *J* = 8.8, 4.2, 2.7 Hz, 1H), 7.16 (t, *J* = 9.1 Hz, 1H), 6.82–6.69 (m, 3H), 5.12 (s, 1H), 4.20 (t, *J* = 6.0 Hz, 2H), 3.08 (d, *J* = 11.5 Hz, 2H), 3.01 (d, *J* = 1.0 Hz, 2H), 2.94–2.81 (m, 4H), 2.24–2.11 (m, 2H), 1.79–1.70 (m, 2H), 1.48 (s, 6H), 1.43–1.24 (m, 3H). ^13^C NMR (126 MHz, CDCl_3_): δ 158.3, 156.3 (^1^*J*_C-F_ = 253.95 Hz), 147.8, 143.4, 134.8, 134.7 (^3^*J*_C-F_ = 8.57 Hz), 130.2, 130.1, 130.1 (^5^*J*_C-F_ = 3.75 Hz), 129.6, 129.4 (^3^*J*_C-F_ = 15.4 Hz), 128.7, 120.5, 118.6, 118.4, 118.2 (^2^*J*_C-F_ = 23.3 Hz), 113.4, 87.6, 66.2, 57.2, 53.5, 48.68, 43.4, 35.9, 29.2, 28.4.

##### 5-Chloro-2-methoxy-*N*-({1-[2-(2,2-dimethyl-2,3-dihydrobenzofuran-7-yloxy)ethyl]piperidin-4-yl}methyl)benzenesulfonamide (**17**)

General Procedure D was followed with primary amine **5** (49.7 mg, 0.146 mmol, 1 eq), 5-chloro-2-methoxybenzenesulfonyl chloride (35.0 mg, 0.146 mmol, 1 eq), and previously ground K_2_CO_3_ (40.3 mg, 0.292 mmol, 2 eq) to afford final compound **17** as a yellow solid, 56.4 mg (76% isolated yield).

C_25_H_33_ClN_2_O_5_S, MW: 509.06, Monoisotopic: 508.18. UPLC/MS purity 96%, t_R_ = 5.57 min, [M+H]^+^ 509.3. ^1^H NMR (500 MHz, CDCl_3_): δ 7.88–7.84 (m, 1H), 7.54–7.47 (m, 1H), 7.17–6.94 (m, 1H), 6.79–6.71 (m, 3H), 5.11–5.00 (m, 1H), 4.22 (dd, *J* = 13.8, 6.1 Hz, 2H), 3.96 (s, 2H), 3.16–3.08 (m, 2H), 3.01 (s, 2H), 2.95–2.84 (m, 3H), 2.75 (t, *J* = 6.7 Hz, 1H), 2.26–2.15 (m, 2H), 1.75 (t, *J* = 17.9 Hz, 2H), 1.58–1.52 (m, 1H), 1.48 (s, 6H), 1.38–1.33 (m, 2H).^13^C NMR (126 MHz, CDCl_3_): δ 154.7, 147.9, 134.3, 130.3, 130.2, 128.7, 128.7, 128.6, 126.1, 120.5, 120.5, 118.3, 113.6, 87.5, 87.5, 66.5, 66.3, 57.1, 56.9, 53.6, 53.5, 48.8, 48.7, 43.3, 28.4. Mp for C_25_H_33_ClN_2_O_5_S: 189.0–191.5 °C.

##### 1-Naphthalene-*N*-({1-[2-(2,2-dimethyl-2,3-dihydrobenzofuran-7-yloxy)ethyl]piperidin-4-yl}methyl)sulfonamide (**18**)

General Procedure D was followed with primary amine **5** (50.5 mg, 0.148 mmol, 1 eq), 1-naphthalenesulfonyl chloride (33.6 mg, 0.148 mmol, 1 eq), and previously ground K_2_CO_3_ (40.9 mg, 0.296 mmol, 2 eq) to afford final compound **18** as a colorless oil, 53.5 mg (73% isolated yield).

C_28_H_34_N_2_O_4_S, MW: 494.65, Monoisotopic Mass: 494.2. UPLC/MS purity 100%, t_R_ = 5.61 min, [M+H]^+^ 495.3. ^1^H NMR (500 MHz, CDCl_3_): δ 8.63 (dd, *J =* 8.6, 1.1 Hz, 1H), 8.23 (dd, *J =* 7.3, 1.3 Hz, 1H), 8.06 (d, *J =* 8.2 Hz, 1H), 7.94 (dd, *J =* 8.2, 1.4 Hz, 1H), 7.68–7.51 (m, 3H), 6.82–6.60 (m, 3H), 4.99–4.94 (m, 1H), 4.17 (t, *J =* 5.9 Hz, 2H), 2.99 (s, 4H), 2.83 (d, *J =* 6.2 Hz, 2H), 2.76 (t, *J =* 6.6 Hz, 2H), 2.11–2.02 (m, 2H), 1.62–1.55 (m, 3H), 1.47 (s, 1H), 1.46 (s, 6H). ^13^C NMR (126 MHz, CDCl_3_): δ 147.9, 143.3, 134.6, 134.4, 134.4, 129.8, 129.3, 128.7, 128.6, 128.2, 127.1, 124.4, 124.3, 120.5, 118.2, 113.6, 87.5, 66.3, 57.0, 53.5, 48.6, 43.4, 29.8, 28.4.

##### 4-Isoquinoline-*N*-({1-[2-(2,2-dimethyl-2,3-dihydrobenzofuran-7-yloxy)ethyl]piperidin-4-yl}methyl)sulfonamide (**19**)

General Procedure D was followed with primary amine **5** (50.4 mg, 0.148 mmol, 1 eq), 4-isoquinolinsulfonyl chloride (33.7 mg, 0.148 mmol, 1 eq), and previously ground K_2_CO_3_ (40.9 mg, 0.296 mmol, 2 eq) to afford final compound **19** as white solid, 68.9 mg (94% isolated yield).

C_27_H_33_N_3_O_4_S, MW: 495.63, Monoisotopic Mass: 495.22. UPLC/MS purity 100%, t_R_ = 4.85 min, [M+H]^+^ 496.3. ^1^H NMR (500 MHz, CDCl_3_): δ 9.41 (d, *J* = 0.9 Hz, 1H), 9.11 (s, 1H), 8.62 (dq, *J* = 8.6, 0.9 Hz, 1H), 8.10 (dt, *J* = 8.2, 1.0 Hz, 1H), 7.88 (ddd, *J* = 8.4, 6.9, 1.3 Hz, 1H), 7.74 (ddd, *J* = 8.1, 7.0, 1.0 Hz, 1H), 6.79–6.67 (m,3H), 5.51 (s, 1H), 4.17 (t, *J* = 6.0 Hz, 2H), 2.99 (s, 5H), 2.86 (t, *J* = 5.6 Hz, 2H), 2.82 (t, *J* = 6.0 Hz, 2H), 2.10 (t, *J* = 11.8 Hz, 2H), 1.67–1.60 (m, 2H), 1.46 (s, 6H), 1.29–1.24 (m, 2H). ^13^C NMR (126 MHz, CDCl_3_): δ 158.0, 147.8, 144.8, 143.3, 133.0, 130.9, 129.7, 129.0, 128.9, 128.7, 128.6, 123.9, 120.5, 118.2, 113.5, 87.5, 66.3, 57.1, 53.5, 48.6, 43.3, 35.7, 29.2, 28.3. Mp for C_27_H_33_N_3_O_4_S: 114.9–116.0 °C.

### 3.2. In Vitro Pharmacology

#### 3.2.1. Determination of the Affinity of the Test Compounds at the α_1_- and α_2_-ARs

The affinity of the obtained compounds were evaluated by radioligand binding assays (the ability to displace [^3^H]prazosin and [^3^H]clonidine from α_1_- and α_2_-adrenoreceptors, respectively) on rat cerebral cortex [33,44]. The brains were homogenized in 20 volumes of ice-cold 50 mM Tris–HCl buffer (pH 7.6) and centrifuged at 20,000 × g for 20 min (0–4 °C). The cell pellet was resuspended in the Tris–HCl buffer and centrifuged again. Radioligand binding assays were performed in plates (MultiScreen/Millipore). The final incubation mixture (final volume 300 μL) consisted of 240 μL of the membrane suspension, 30 μL of [^3^H]prazosin (0.2 nM) or [^3^H]clonidine (2 nM) solution, and 30 μL of the buffer containing seven to eight concentrations (10^−11^–10^−4^M) of the tested compounds. For measuring the unspecific binding, phentolamine, 10 μM (in the case of [^3^H]prazosin) and clonidine, 10 μM (in the case of [^3^H]clonidine) were applied. The incubation was terminated by rapid filtration over glass fibre filters (Whatman GF/C) using a vacuum manifold (Millipore). The filters were then washed twice with the assay buffer and placed in scintillation vials with a liquid scintillation cocktail. Radioactivity was measured in a WALLAC 1409 DSA liquid scintillation counter. All assays were made in duplicate.

#### 3.2.2. Determination of the Affinity of the Test Compounds at the Serotonin 5-HT_1A_, 5-HT_2A_, 5-HT_6_, 5-HT_7_ and Dopaminergic D_2_ Receptors

All experiments were performed using HEK293 cells stably expressing human 5-HT_1A_, 5-HT_6_, 5-HT_7b_, and D_2L_ receptors or CHO-K1 cells with a plasmid containing the human 5-HT_2A_ receptor coding sequence (PerkinElmer, Waltham, MA, USA), according to previously reported procedures [38,45,46]. Cells were cultured in 150 cm^2^ flasks and, after reaching a 90% confluence, were washed with PBS and the centrifugated (200 g) in PBS containing 0.1 mM EDTA and 1 mM dithiothreitol. Cell pellets were subsequently homogenized (in Ultra Turrax tissue homogenizer) and centrifugated twice (35,000× *g* for 15 min at 4 °C), with 15 min incubation at 37 °C between the centrifugations. All assays were incubated in dedicated buffers and in a total volume of 200 µL in 96-well microtitre plates for 1 h at 37 °C, except for 5-HT_1A_R and 5-HT_2A_R that was incubated for 1 h at room temperature. The process of equilibration is terminated by rapid filtration through Unifilter-96 (PerkinElmer) plates with a 96-well cell harvester and radioactivity retained on the filters was quantified on a Microbeta plate reader (PerkinElmer, USA). For displacement studies, the following assay samples containing the proper radioligand were used: 2.5 nM [^3^H]-8-OH-DPAT (PerkinElmer, #NET929001MC) for 5-HT_1A_R; 1 nM [^3^H]-ketanserin (PerkinElmer, #NET791250UC) for 5-HT_2A_R; 2 nM [^3^H]-LSD (PerkinElmer, #NET638250UC) for 5-HT_6_R; and 0.8 nM [^3^H]-5-CT (PerkinElmer, #NET1188U100UC) for 5-HT_7_R or 2.5 nM [^3^H]-raclopride (PerkinElmer, #NET975001MC) for D_2L_R. Non-specific binding was determined using 10 μM 5-HT for 5-HT_1A_R and 5-HT_7_R, 20 μM mianserin for 5-HT_2A_R, 10 μM methiothepine for 5-HT_6_R, and 10 μM haloperidol for D_2L_R. Each compound was tested in triplicate at seven concentrations ranging from 10^−10^ to 10^−4^ M. The inhibition constants (*K*_i_) were calculated from the Cheng–Prusoff equation [47]. Results were expressed as means of at least three separate experiments.

#### 3.2.3. Determination of the Intrinsic Activity of the Test Compounds at the α_2_-AR Subtypes

Intrinsic activity assays for α_2A_-adrenergic receptor were performed according to the instructions of the manufacturer of the assay kit containing the ready to use cells with stable expression of the α_2A_-adrenoceptor (Invitrogen, Life Technologies, Waltham, MA, USA). Tango™ ADRA2A-bla U2OS DA cells (10,000 cells/well) were plated in a 384-well format and incubated for 20 h. Cells were exposed to Yohimbine (Sigma-Aldrich, Merck, Darmstadt, Germany) for 30 min, then stimulated with an EC_80_ concentration of UK14,304 (Sigma-Aldrich) in the presence of 0.1% DMSO for 5 h. Cells were then loaded with LiveBLAzer™-FRET B/G substrate for 2 h. Fluorescence emission values at 460 nm and 530 nm were obtained using a standard fluorescence plate reader and the % inhibition plotted against the indicated concentrations of Yohimbine.

The intrinsic activity assays for the α_2B_-adrenergic receptor were assessed by luminescence detection of calcium mobilization using the recombinant expressed jellyfish photoprotein, aequorin. Measurements were performed with adrenergic α_2B_ AequoScreen cell line (PekinElmer). The cell density in 96-well format measurements was 5000 cells per well. Cell harvesting, coelenterazine h (Invitrogen, cat. no. C 6780) loading and preparation were done according to instructions presented in the AequoScreen Starter Kit Manual (PerkinElmer). Compound concentration series (50 μL/well) were diluted in 0.1% BSA (Intergen) containing assay buffer (D-MEM/F-12, Invitrogen cat. no. 11039) and prepared in white ½ Area Plate–96 well microplates (PerkinElmer,). The cell suspension was dispensed on the ligands using the POLARstar optima reader injectors. For the antagonist assay, cells were injected (50 μL) into the assay plate with antagonists (50 μL) using the POLARstar optima reader. The antagonist dilution series with four replicates were prepared as instructed in the AequoScreen Starter Kit Manual at the concentrations from 10^–11^ to 10^–6^ M/L. The agonist used for α_2A_-AR cells was Oxymetazoline (Sigma, cat. no. O2378), which at a single concentration was injected (50 μL, final concentration EC_80_) on the preincubated (15–20 min) mixture of cells and antagonist, and the emitted light was recorded for 20 s.

#### 3.2.4. Determination of the Intrinsic Activity of the Test Compounds at the 5-HT_7_R

Cells (prepared with the use of Lipofectamine 2000) were maintained at 37 °C in a humidified atmosphere with 5% CO_2_ and grown in Dulbeco’s Modifier Eagle Medium containing 10% dialyzed fetal bovine serum and 500 mg/mL G418 sulphate. For functional experiments, cells were subcultured in 25 cm diameter dishes, grown to 90% confluence, washed twice with pre-warmed to 37 °C phosphate buffered saline (PBS) and were centrifuged for 5 min (160× *g*). The supernatant was aspirated, then the cell pellet was resuspended in stimulation buffer (1 × HBSS, 5 mM HEPES, 0.5 mM IBMX, 0.1% BSA). The cAMP level was measured using the LANCE cAMP detection kit (PerkinElmer), according to the manufacturer’s directions. For the investigation of the antagonist effect on 5-HT_7_R, the agonist 5-carboxyamidotryptamine (5-CT; EC_50_ = 1 nM) was used in submaximal concentration (10nM) to stimulate cAMP production. Cells (5 µL) were incubated with compounds (5 µL) for 30 min at room temperature in 384-well white opaque microtiter plate. After incubation, the reaction was stopped, and cells were lysed by the addition of 10 µL working solution (5 µL Eu-cAMP and 5 µL ULight-anti-cAMP). The assay plate was incubated for 1 h at room temperature. Time-resolved fluorescence resonance energy transfer (TR-FRET) was detected by an Infinite M1000 Pro (Tecan) using instrument settings from LANCE cAMP detection kit manual. *K*_b_ values were calculated from the Cheng–Prusoff equation specific for the analysis of functional inhibition curves: *K*_b_ = IC_50_/(1+A/EC_50_) where A is the agonist concentration, IC_50_ is the concentration of the antagonist producing a 50% reduction in the response to the agonist, and EC_50_ is the agonist concentration which causes half of the maximal response [47].

### 3.3. In Vivo Pharmacology

#### 3.3.1. Animals

Adult male Albino Swiss mice (CD-1, 8 weeks old, 25–30 g: Jagiellonian University Medical College, Krakow, Poland) were used in the study. Animals were housed in groups of 8 in a transparent plastic cage (382 × 220 × 150 mm) at room temperature (22 ± 2 °C), on a 12 h light/dark cycle with ad libitum access to food and water. Mice were handled for one week before starting the experimental procedures. The separate groups of animals were used in the forced swim test and in the locomotor studies. All studies were approved by the Institutional Animal Care and Ethics Committee of the Jagiellonian University (approval no.: 80/2015).

#### 3.3.2. Drug Administration

Mirtazapine (16 mg/kg or 4 mg/kg, Sigma-Aldrich) was dissolved in DMSO and diluted to the appropriate dose with 1% Tween 80, immediately before use (the maximal final DMSO concentration was 2%). Tested compounds were dissolved with 1% Tween 80. Solutions of mirtazapine and the tested compounds were administered intraperitoneally (*ip*) 30 min prior to the experiment. The control animals were given *ip* injections of the 2% DMSO in 1% Tween 80 (vehicle). The volume of vehicle or drug solutions was 10 mL/kg.

#### 3.3.3. Forced Swim Test

The experiment was performed on mice according to the method previously described [48,49]. Mice were forced to swim individually in the glass cylinders (height 25 cm, diameter 10 cm) filled with water at 24 ± 1 °C to a depth of 10 cm and left there for 6 min. Following a 2 min habituation period, total time spent immobile was recorded over the next 4 min. The animal was regarded as immobile when it remained floating passively in the water, making only small movements to keep its head above the water.

#### 3.3.4. Spontaneous Locomotor Activity

Locomotor activity was recorded with an Opto M3 multichannel activity monitor, photoresistor actometers connected to a counter for the recording of light-beam interruptions (MultiDevice Software v1.3, Columbus Instruments, Columbus, OH, USA). The mice, after being placed into the cages individually, had their activity evaluated between the 2nd and the 6th minute. The chosen time period corresponded with the time interval considered in the FST. Spontaneous locomotor activity was evaluated as the distance travelled plus the movements of climbing by animals.

#### 3.3.5. Statistical Analysis

Statistical calculations were performed using GraphPad Prism 6 software (GraphPad Software, San Diego, CA, USA). The normality of data sets was determined using Shapiro–Wilk test. Comparisons between experimental and control groups were performed by one-way ANOVA, followed by Dunnett post hoc.

## 4. Conclusions

Based on the finding that concurrent blockade of α_2_-AR and 5-HT_7_R might be beneficial in the treatment of depressive disorders, we elaborated a medicinal mechanochemical approach to provide a limited series of arylsulfonamides of (dihydrobenzofuranoxy)ethyl piperidines as dual acting α_2_/5-HT_7_R antagonists. Sustainable solid-state protocol furnished designed compounds **6**–**19** in high yields and purities, limiting the amount of organic solvents as well as the formation of by-products. Further focused SAR studies revealed that the presence of a 4-aminopiperidine central core, together with di-halogenated substituents or a 4-isoquinolyl moiety at the sulfonamide fragment, were responsible for the high affinity of tested compounds for both biological targets. Finally, the study identified 5-chloro-2-fluoro-*N*-{1-[2-(2,2-dimethyl-2,3-dihydrobenzofuran-7-yloxy)ethyl]piperidin-4-yl}benzenesulfonamide (compound **8**) as a potent α_2A_/5-HT_7_R antagonist, which produced an antidepressant-like effect in FST in mice. The effect was similar to that produced by mirtazapine used in a two-fold higher dose, without inducing sedation. Preliminary data for compound **8** are promising enough to warrant further efficacy and safety studies on the potential of dual-acting α_2A_/5-HT_7_R antagonists in the treatment of affective disorders.

## Figures and Tables

**Table 1:**
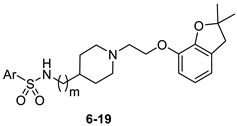
The binding data of the synthesized compounds **6**–**19** for α_2_-AR and 5-HT_7_R.

Compound	Ar	m	*K*_i_ [nM] ± SEM	
			α_2_^a^	5-HT_7_^b^
**6**	4-F-phenyl	0	138 ± 44	35 ± 22
**7**	3-Cl-phenyl	0	649 ± 62	64 ± 15
**8**	5-Cl,2-F-phenyl	0	148 ± 23	30 ± 11
**9**	5-Cl,2-MeO-phenyl	0	573 ± 56	86 ± 26
**10**	2,5-diMeO-phenyl	0	244 ± 52	95 ± 18
**11**	1-naphthyl	0	366 ± 46	50 ± 16
**12**	2-naphthyl	0	200 ± 51	67 ± 21
**13**	4-isoquinolyl	0	80 ± 42	91 ± 25
**14**	4-F-phenyl	1	1194 ± 44	727 ± 65
**15**	3-Cl-phenyl	1	743 ± 32	664 ± 82
**16**	5-Cl,2-F-phenyl	1	154 ± 28	316 ± 73
**17**	5-Cl,2-MeO-phenyl	1	1097 ± 78	488 ± 70
**18**	1-naphthyl	1	907 ± 64	366 ± 69
**19**	4-isoquinolyl	1	298 ± 24	387 ± 58
Clonidine			2.7 ± 0.3	NT ^c^
SB-267790			NT^c^	3 ± 0.5
Mirtazapine			112 **^d^**	265 ^e^

^a^*K*_i_ ± SEMs values based on three independent binding experiments in rat cerebral cortex; ^b^ *K*_i_ ± SEMs values based on three independent binding experiments in HEK-293 cells; ^c^ Not Tested; ^d^ Data taken from [34] with binding experiments performed in rat cerebral cortex; ^e^ Data taken from [35] with binding experiments performer in cloned human receptors.

**Table 2:** The binding data of selected compounds **6**, **8** and **13** for α_1_-AR, 5-HT_1A_, 5-HT_2A_, 5-HT_6_ and D_2_Rs.

Compd	*K*_i_ [nM]				
	α_1_^a^	5-HT_1A_^b^	5-HT_2A_^b^	5-HT_6_^b^	D_2_^b^
**6**	761 ± 86	221 ± 25	538 ± 38	839 ± 70	327 ± 29
**8**	1256 ± 101	260 ± 29	1420 ± 98	873 ± 91	388 ± 37
**13**	429 ± 66	260 ± 18	1422 ± 105	1123 ± 114	326 ± 25

^a^*K*_i_ ± SEMs values based on three independent binding experiments in rat cerebral cortex; ^b^ *K*_i_ ± SEMs values based on three independent binding experiments in HEk-293 cells.

**Table 3:** The antagonist activity of selected compounds **6**, **8**, **13** and reference yohimbine at α_2A_-AR, α_2B_-AR and 5-HT_7_R.

Compd	α_2_	α_2A_	α_2B_	5-HT_7_	
	*K*_i_ [nM] ^a^	*K*_b_ [nM] ^b^	*K*_b_ [nM] ^c^	*K*_i_ [nM] ^d^	*K*_b_[nM] ^e^
**6**	138 ± 44	12 ± 4	103 ± 25	35 ± 22	186 ± 37
**8**	148 ± 23	40 ± 11	142 ± 31	30 ± 11	141 ± 43
**13**	80 ± 42	31 ± 8	27 ± 13	91 ± 25	155 ± 30
**Yohimbine**	164 ± 55	0.81 ± 0.3	2.99 ± 0.7	NT ^f^	NT ^f^

^a^*K*_i_ ± SEMs values based on three independent binding experiments in rat cerebral cortex; ^b^ *K*_b_ ± SEMs values based on two independent experiments in ADRA2A-bla U2OS DA cells; ^c^ *K*_b_ ± SEMs values based on two independent experiments in AequoScreen cells; ^d^ *K*_i_ ± SEMs values based on three independent binding experiments in HEK293 cells; ^e^ *K*_b_ ± SEMs values based on three independent experiments in HEK293 cells; ^f^ Not Tested.

## Data Availability

The data presented in this study are available in the Supplementary Materials.

## Supplementary Materials

The following are available online: optimization of mechanochemical reactions (Tables S1–S3); MS, ^1^H-NMR and ^13^C-NMR spectra of all intermediates and final compounds (Figures S1–S54).

## Sample Availability

Samples of the compounds are available from the authors.

## References

[1] World Health Organization (2017). Depression and Other Common Mental Disorders: Global Health Estimates.

[2] Conradi H.J., Ormel J., De Jonge P. (2010). Presence of individual (residual) symptoms during depressive episodes and periods of remission: A 3-year prospective study. Psychol. Med..

[3] Philipp M., Brede M., Hein L. (2002). Physiological significance of α2-adrenergic receptor subtype diversity: One receptor is not enough. Am. J. Physiol. Integr. Comp. Physiol..

[4] Bücheler M., Hadamek K., Hein L. (2002). Two α2-adrenergic receptor subtypes, α2A and α2C, inhibit transmitter release in the brain of gene-targeted mice. Neuroscience.

[5] Scheinin M., Lomasney J.W., Hayden-Hixson D.M., Schambra U.B., Caron M.G., Lefkowitz R.J., Fremeau R.T. (1994). Distribution of α2-adrenergic receptor subtype gene expression in rat brain. Mol. Brain Res..

[6] Scheibner J., Trendelenburg A.-U., Hein L., Starke K. (2001). α2 -Adrenoceptors modulating neuronal serotonin release: A study in α2 -adrenoceptor subtype-deficient mice. Br. J. Pharmacol..

[7] Cottingham C., Wang Q. (2012). α2 adrenergic receptor dysregulation in depressive disorders: Implications for the neurobiology of depression and antidepressant therapy. Neurosci. Biobehav. Rev..

[8] Lovenberg T.W., Baron B.M., De Lecea L., Miller J.D., Prosser R., Rea M.A., Foye P.E., Racke M., Slone A.L., Siegel B.W. (1993). A novel adenylyl cyclase-activating serotonin receptor (5-HT7) implicated in the regulation of mammalian circadian rhythms. Neuron.

[9] Guseva D., Wirth A., Ponimaskin E. (2014). Cellular mechanisms of the 5-HT7receptor-mediated signaling. Front. Behav. Neurosci..

[10] Nikiforuk A. (2015). Targeting the Serotonin 5-HT7 Receptor in the Search for Treatments for CNS Disorders: Rationale and Progress to Date. CNS Drugs.

[11] Modica M.N., LaCivita E., Intagliata S., Salerno L., Romeo G., Pittalà V., Leopoldo M. (2018). Structure–Activity Relationships and Therapeutic Potentials of 5-HT7 Receptor Ligands: An Update. J. Med. Chem..

[12] Langer S.Z. (2015). α2-Adrenoceptors in the treatment of major neuropsychiatric disorders. Trends Pharmacol. Sci..

[13] Dwyer J.M., Platt B.J., Rizzo S.J.S., Pulicicchio C.M., Wantuch C., Zhang M.-Y., Cummons T., Leventhal L., Bender C.N., Zhang J. (2010). Preclinical characterization of BRL 44408: Antidepressant—And analgesic-like activity through selective α2A-adrenoceptor antagonism. Int. J. Neuropsychopharmacol..

[14] Guscott M., Bristow L., Hadingham K., Rosahl T., Beer M., Stanton J., Bromidge F., Owens A., Huscroft I., Myers J. (2005). Genetic knockout and pharmacological blockade studies of the 5-HT7 receptor suggest therapeutic potential in depression. Neuropharmacology.

[15] Wesołowska A., Tatarczynska E., Nikiforuk A., Chojnacka-Wójcik E. (2007). Enhancement of the anti-immobility action of antidepressants by a selective 5-HT7 receptor antagonist in the forced swimming test in mice. Eur. J. Pharmacol..

[16] Sanacora G., Berman R.M., Cappiello A., Oren D.A., Kugaya A., Liu N., Gueorguieva R., Fasula D., Charney D.S. (2004). Addition of the α2-Antagonist Yohimbine to Fluoxetine: Effects on Rate of Antidepressant Response. Neuropsychopharmacology.

[17] Dhir A., Kulkarni S. (2007). Effect of Addition of Yohimbine (Alpha-2-Receptor Antagonist) to the Antidepressant Activity of Fluoxetine or Venlafaxine in the Mouse Forced Swim Test. Pharmacology.

[18] Canale V., Partyka A., Kurczab R., Krawczyk M., Kos T., Satała G., Kubica B., Jastrzębska-Więsek M., Wesołowska A., Bojarski A. (2017). Novel 5-HT 7 R antagonists, arylsulfonamide derivatives of (aryloxy)propyl piperidines: Add-on effect to the antidepressant activity of SSRI and DRI, and pro-cognitive profile. Bioorg. Med. Chem..

[19] Marshall R. (1983). The pharmacology of mianserin-an update. Br. J. Clin. Pharmacol..

[20] Anttila S.A., Leinonen E. (2001). V A review of the pharmacological and clinical profile of mirtazapine. CNS Drug Rev..

[21] Canale V., Kurczab R., Partyka A., Satała G., Lenda T., Jastrzębska-Więsek M., Wesołowska A., Bojarski A., Zajdel P. (2016). Towards new 5-HT 7 antagonists among arylsulfonamide derivatives of (aryloxy)ethyl-alkyl amines: Multiobjective based design, synthesis, and antidepressant and anxiolytic properties. Eur. J. Med. Chem..

[22] Tan D., Loots L., Friščić T. (2016). Towards medicinal mechanochemistry: Evolution of milling from pharmaceutical solid form screening to the synthesis of active pharmaceutical ingredients (APIs). Chem. Commun..

[23] Colacino E., Porcheddu A., Charnay C., Delogu F. (2019). From enabling technologies to medicinal mechanochemistry: An eco-friendly access to hydantoin-based active pharmaceutical ingredients. React. Chem. Eng..

[24] Friščić T., Mottillo C., Titi H.M. (2020). Mechanochemistry for Synthesis. Angew. Chem. Int. Ed..

[25] Beillard A., Bantreil X., Métro T.-X., Martinez J., Lamaty F. (2019). Alternative Technologies That Facilitate Access to Discrete Metal Complexes. Chem. Rev..

[26] Milbeo P., Quintin F., Moulat L., Didierjean C., Martinez J., Bantreil X., Calmès M., Lamaty F. (2021). Synthesis, characterisation and cytotoxic activity evaluation of new metal-salen complexes based on the 1,2-bicyclo[2.2.2]octane bridge. Tetrahedron Lett..

[27] Métro T.-X., Martinez J., Lamaty F. (2017). 1,1′-Carbonyldiimidazole and Mechanochemistry: A Shining Green Combination. ACS Sustain. Chem. Eng..

[28] Ardila-Fierro K.J., Hernández J.G. (2021). Sustainability Assessment of Mechanochemistry by Using the Twelve Principles of Green Chemistry. ChemSusChem.

[29] Canale V., Frisi V., Bantreil X., Lamaty F., Zajdel P. (2020). Sustainable Synthesis of a Potent and Selective 5-HT7 Receptor Antagonist Using a Mechanochemical Approach. J. Org. Chem..

[30] Declerck V., Nun P., Martinez J., Lamaty F. (2009). Solvent-Free Synthesis of Peptides. Angew. Chem. Int. Ed..

[31] Zajdel P., Canale V., Partyka A., Marciniec K., Kurczab R., Satała G., Siwek A., Jastrzębska-Więsek M., Wesołowska A., Kos T. (2015). Arylsulfonamide derivatives of (aryloxy)ethylpiperidines as selective 5-HT7 receptor antagonists and their psychotropic properties. MedChemComm.

[32] Rak A., Canale V., Marciniec K., Żmudzki P., Kotańska M., Knutelska J., Siwek A., Stachowicz G., Bednarski M., Nowiński L. (2016). Arylsulfonamide derivatives of (aryloxy)ethyl pyrrolidines and piperidines as α 1 -adrenergic receptor antagonist with uro-selective activity. Bioorg. Med. Chem..

[33] Canale V., Rak A., Kotańska M., Knutelska J., Siwek A., Bednarski M., Nowiński L., Zygmunt M., Koczurkiewicz P., Pękala E. (2018). Synthesis and Pharmacological Evaluation of Novel Silodosin-Based Arylsulfonamide Derivatives as α1A/α1D-Adrenergic Receptor Antagonist with Potential Uroselective Profile. Molecules.

[34] De Boer T., Maura G., Raiteri M., De Vos C., Wieringa J., Pinder R. (1988). Neurochemical and autonomic pharmacological profiles of the 6-aza-analogue of mianserin, org 3770 and its enantiomers. Neuropharmacology.

[35] Fernandez J., Alonso J.M., Andres J.I., Cid J.M., Diaz A., Iturrino L., Gil P., Megens A., Sipido V.K., Trabanco A.A. (2005). Discovery of New Tetracyclic Tetrahydrofuran Derivatives as Potential Broad-Spectrum Psychotropic Agents. J. Med. Chem..

[36] Zajdel P., Marciniec K., Satała G., Canale V., Kos T., Partyka A., Jastrzębska-Więsek M., Wesołowska A., Basińska-Ziobroń A., Wójcikowski J. (2016). N1-Azinylsulfonyl-1H-indoles: 5-HT6 Receptor Antagonists with Procognitive and Antidepressant-Like Properties. ACS Med. Chem. Lett..

[37] Partyka A., Kurczab R., Canale V., Satała G., Marciniec K., Pasierb A., Jastrzębska-Więsek M., Pawłowski M., Wesołowska A., Bojarski A. (2017). The impact of the halogen bonding on D 2 and 5-HT 1A /5-HT 7 receptor activity of azinesulfonamides of 4-[(2-ethyl)piperidinyl-1-yl]phenylpiperazines with antipsychotic and antidepressant properties. Bioorg. Med. Chem..

[38] Zajdel P., Kos T., Marciniec K., Satała G., Canale V., Kamiński K., Hołuj M., Lenda T., Koralewski R., Bednarski M. (2018). Novel multi-target azinesulfonamides of cyclic amine derivatives as potential antipsychotics with pro-social and pro-cognitive effects. Eur. J. Med. Chem..

[39] Campbell S., MacQueen G. (2004). The role of the hippocampus in the pathophysiology of major depression. J. Psychiatry Neurosci..

[40] Marcinkowska M., Kotańska M., Zagórska A., Śniecikowska J., Kubacka M., Siwek A., Bucki A., Pawłowski M., Bednarski M., Sapa J. (2018). Synthesis and biological evaluation of N-arylpiperazine derivatives of 4,4-dimethylisoquinoline-1,3(2H,4H)-dione as potential antiplatelet agents. J. Enzym. Inhib. Med. Chem..

[41] Bogdanova O.V., Kanekar S., D’Anci K.E., Renshaw P.F. (2013). Factors influencing behavior in the forced swim test. Physiol. Behav..

[42] Watanabe N., Omori I.M., Nakagawa A., Cipriani A., Barbui C., Churchill R., Furukawa T.A. (2011). Mirtazapine versus other antidepressive agents for depression. Cochrane Database Syst. Rev..

[43] Murphy M.J., Peterson M.J. (2015). Sleep Disturbances in Depression. Sleep Med. Clin..

[44] Maj J., Klimek V., Nowak G. (1985). Antidepressant drugs given repeatedly increase binding to α1-adrenoceptors in the rat cortex. Eur. J. Pharmacol..

[45] Kurczab R., Canale V., Satała G., Zajdel P., Bojarski A.J. (2018). Amino Acid Hot Spots of Halogen Bonding: A Combined Theoretical and Experimental Case Study of the 5-HT7 Receptor. J. Med. Chem..

[46] Hogendorf A.S., Hogendorf A., Kurczab R., Kalinowska-Tłuścik J., Popik P., Nikiforuk A., Krawczyk M., Satała G., Lenda T., Knutelska J. (2019). 2-Aminoimidazole-based antagonists of the 5-HT6 receptor—A new concept in aminergic GPCR ligand design. Eur. J. Med. Chem..

[47] Yung-Chi C., Prusoff W.H. (1973). Relationship between the inhibition constant (KI) and the concentration of inhibitor which causes 50 per cent inhibition (I50) of an enzymatic reaction. Biochem. Pharmacol..

[48] Porsolt R.D., Bertin A., Jalfre M. (1977). Behavioral despair in mice: A primary screening test for antidepressants. Arch. Int. Pharmacodyn. Ther..

[49] Pytka K., Socala K., Rapacz A., Nieoczym D., Pieróg M., Gryboś A., Siwek A., Waszkielewicz A., Wlaź P. (2017). HBK-14 and HBK-15, triple 5-HT_1A_, 5-HT_7_ and 5-HT_3_ antagonists with potent antidepressant- and anxiolytic-like properties, increase seizure threshold in various seizure tests in mice. Prog. Neuro Psychopharmacol. Biol. Psychiatry.

