# Supplementary material for: Design, Sustainable Synthesis and Biological Evaluation of a Novel Dual α2A/5-HT7 Receptor Antagonist with Antidepressant-Like Properties
Source: Molecules. 2021 Jun 23;26(13):3828. doi: 10.3390/molecules26133828 (PMC8270334; doi:10.3390/molecules26133828)
Supplement: Supplementary file 1 [file molecules-26-03828-s001.zip › molecules-1254488-supplementary.pdf]

# *Supplementary Materials*

# Design, Sustainable Synthesis and Biological Evaluation of a Novel Dual $\alpha_{2A}$/5-$HT_7$ Receptor Antagonist

**Vittorio Canale [1], Magdalena Kotańska [2], Anna Dziubina [2], Matylda Stefaniak [1], Agata Siwek [3], Gabriela Starowicz [3], Krzysztof Marciniec [4], Patryk Kasza [1], Grzegosz Satała [5], Beata Duszyńska [5], Xavier Bantreil [6], Frédéric Lamaty [6], Marek Bednarski [2], Jacek Sapa [2] and Paweł Zajdel [1,*]**

[1] Department of Organic Chemistry, Faculty of Pharmacy, Jagiellonian University Medical College, 9 Medyczna Street, 30-688 Kraków, Poland
[2] Department of Pharmacodynamics, Faculty of Pharmacy, Jagiellonian University Medical College, 9 Medyczna Street, 30-688 Kraków, Poland
[3] Department of Pharmacobiology, Faculty of Pharmacy, Jagiellonian University Medical College, 9 Medyczna Street, 30-688 Kraków, Poland
[4] Department of Organic Chemistry, Medical University of Silesia, 4 Jagiellońska Street, 41-200 Sosnowiec, Poland
[5] Department of Medicinal Chemistry, Maj Institute of Pharmacology, Polish Academy of Sciences, 12 Smętna Street, 31-343 Kraków, Poland
[6] IBMM, Université de Montpellier, CNRS, ENSCM, 34095 Montpellier, France

**Table S1.** Optimization of milling conditions for alkylation of 4-Boc-*N*-aminopiperidine.

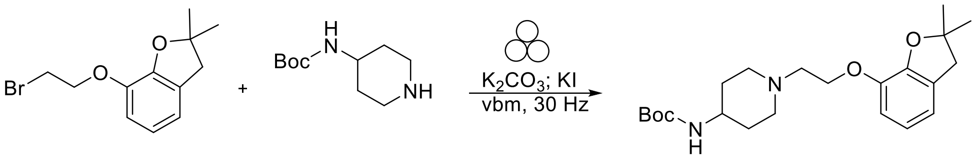


**1** **2**

| | **Amine eq** | **$K_2CO_3$ eq** | **KI eq** | **Time [min]** | **% conversion for 2[a]** |
|---|---|---|---|---|---|
| **1** | 1 | 3 | 0.5 | 140 | 74 |
| **2** | 1.2 | 3 | - | 20 | 0 |
| **3** | 1.2 | 3 | 0.5 | 20 | 4 |
| **4** | 1.2 | 3 | - | 100 | 15 |
| **5** | 1.2 | 3 | 0.5 | 100 | 49 |
| **6** | 1.2 | 3 | - | 120 | 19 |
| **7** | 1.2 | 3 | 0.5 | 120 | 82 |
| **8** | 1.2 | 3 | - | 140 | 23 |
| **9** | 1.2 | 3 | 0.5 | 140 | 87 |
| **19** | 1.2 | 2 | 0.5 | 140 | 87 |
| **11** | 1.2 | 2 | 0.25 | 140 | 36 |
| **12[b]** | 1.2 | 2 | 0.5 | 140 | 36 |
| **13[b]** | 1.2 | 2 | 0.5 | 210 | 69 |
| **14[b]** | 1.2 | 3 | 0.5 | 140 | 57 |
| **15[b]** | 1.2 | 3 | 0.5 | 210 | 97 (84)[c] |

Reaction conditions: vbm 30 Hz, $\phi_{ball}$ = 1.5 cm, total mass of reagents = 125 mg, milling load 15 mg/mL, 10 mL PTFE jar; [a]Conversions were determined by HPLC, [b]Reaction conditions: vbm 30 Hz, $\phi_{ball}$ = 1.5 cm, total mass of reagents = 500 mg, 35 mL PTFE jar, [c]Yield for isolated compound.

**Table S2.** Optimization of milling conditions for alkylation of 4-Boc-*N* aminomethylpiperidine.

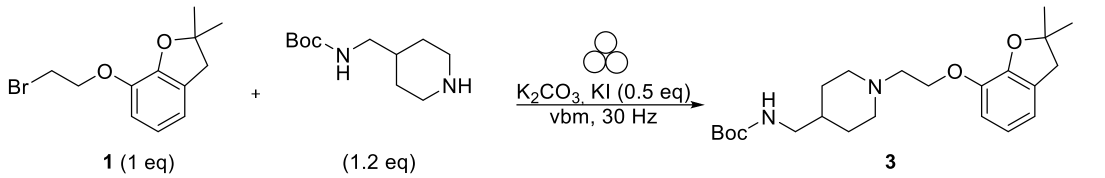


| entry | base ($K_2CO_3$) (eq) | time [min] | % conversion for 3[a] |
| --- | --- | --- | --- |
| 1 | 2 | 140 | 62 |
| 2 | 3 | 140 | 82 |
| 3 | 3 | 210 | 84 (81)[b] |

Reaction conditions: vbm 30 Hz, $\phi_{ball}$ = 1.5 cm, total mass of reagents = 500 mg, milling load 15 mg/mL, 35 mL PTFE jar, [a]Conversions were determined by HPLC, [b]Yield for isolated compound

**Table S3.** Optimization of milling conditions for sulfonylation of primary amine.

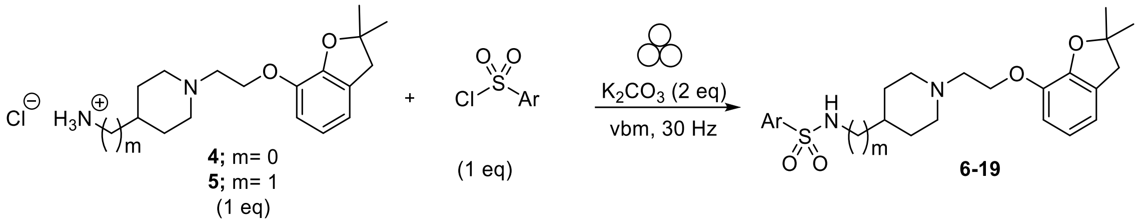


| entry | product | Ar | time [min] | % conversion[a] | % yield[b] |
| --- | --- | --- | --- | --- | --- |
| 1 | 6 | 4-F-phenyl | 1 | 91 | 89 |
| 2 | 7 | 3-Cl-phenyl | 1 | 88 | 86 |
| 3 | 8 | 5-Cl,2-F-phenyl | 5 | 69 | 68 |
| 4 | 9 | 5-Cl,2-MeO-phenyl | 10 | 84 | 84 |
| 5 | 10 | 2,5-diMeO-phenyl | 5 | 79 | 70 |
| 6 | 11 | 1-naphtyl | 5 | 74 | 69 |
| 7 | 12 | 2-naphtyl | 5 | 65 | 65 |
| 8 | 13 | 4-isoquinolyl | 5 | 85 | 82 |
| 9 | 14 | 4-F-phenyl | 1 | 91 | 90 |
| 10 | 15 | 3-Cl-phenyl | 1 | 82 | 83 |
| 11 | 16 | 5-Cl,2-F-phenyl | 5 | 70 | 67 |
| 12 | 17 | 5-Cl,2-MeO-phenyl | 10 | 78 | 76 |
| 13 | 18 | 1-naphtyl | 5 | 78 | 73 |
| 14 | 19 | 4-isoquinolyl | 5 | 98 | 94 |

Reaction conditions: vbm 30 Hz, $\phi_{ball}$ = 1.5 cm, total mass of reagents = 125 mg, milling load 15 mg/mL, 10 mL PTFE jar, [a]Conversions were determined by HPLC, [b]Yield for isolated compound.

## 1. MS, ¹H-NMR and ¹³C-NMR for intermediates and final compounds

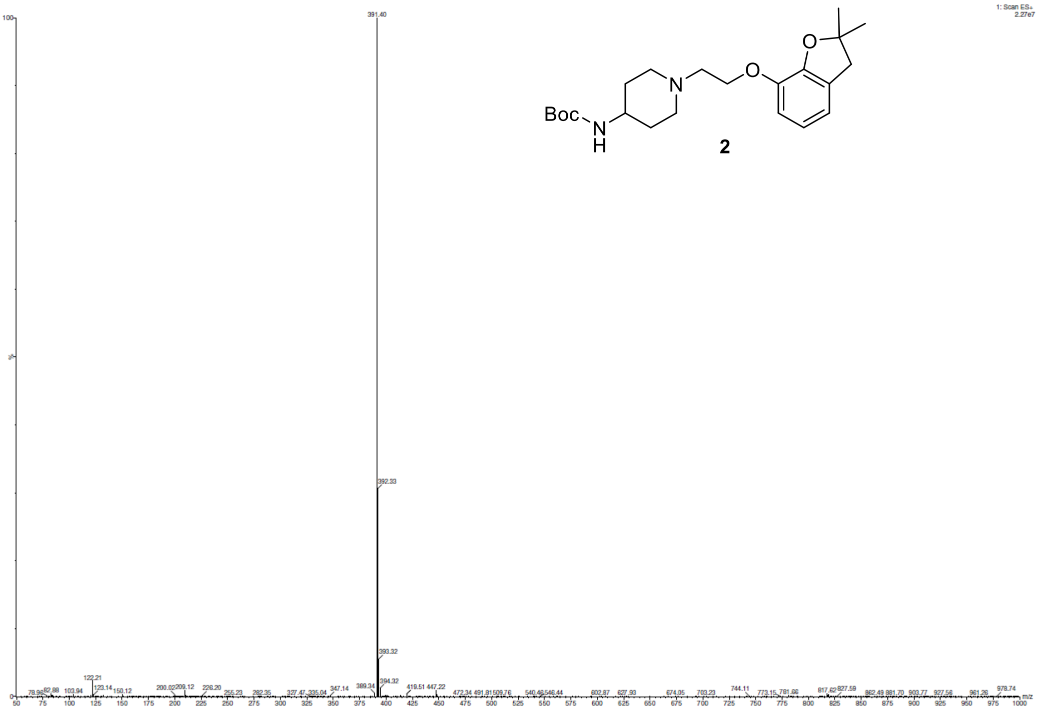

**Figure S1.** MS spectra for *tert*-butyl {1-[2-(2,2-dimethyl-2,3-dihydrobenzofuran-7-yloxy)ethyl]piperidin-4-yl}carbamate (**2**).

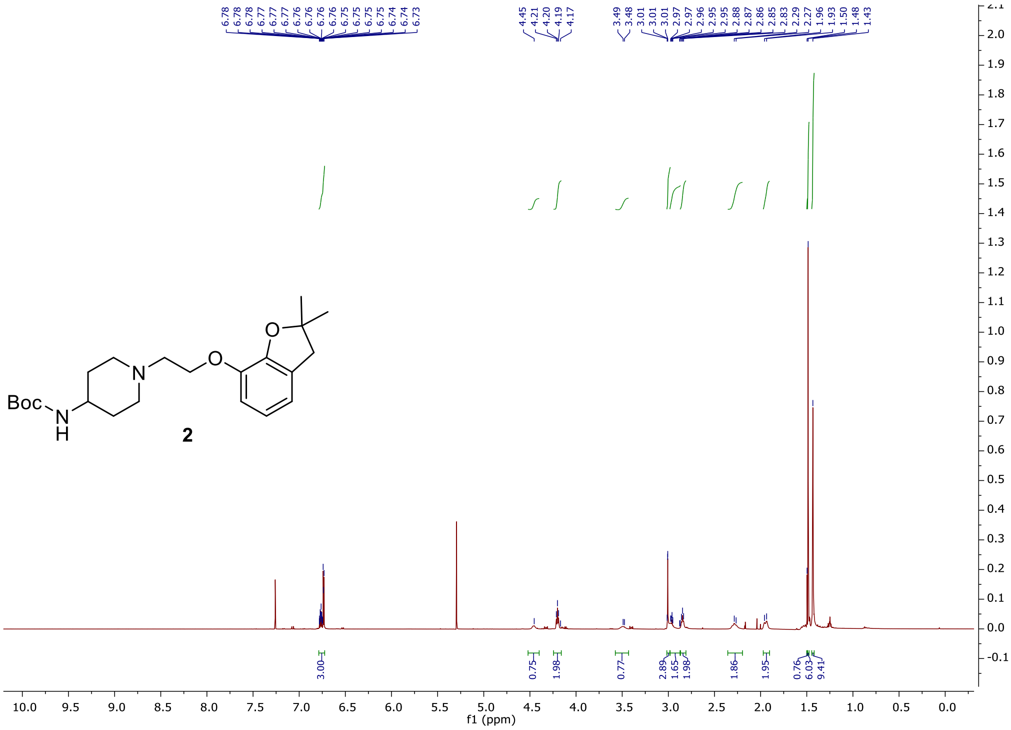

**Figure S2.** ¹H-NMR spectra (500 MHz, $CDCl_3$) for *tert*-butyl {1-[2-(2,2-dimethyl-2,3-dihydrobenzofuran-7-yloxy)ethyl]piperidin-4-yl}carbamate (**2**).

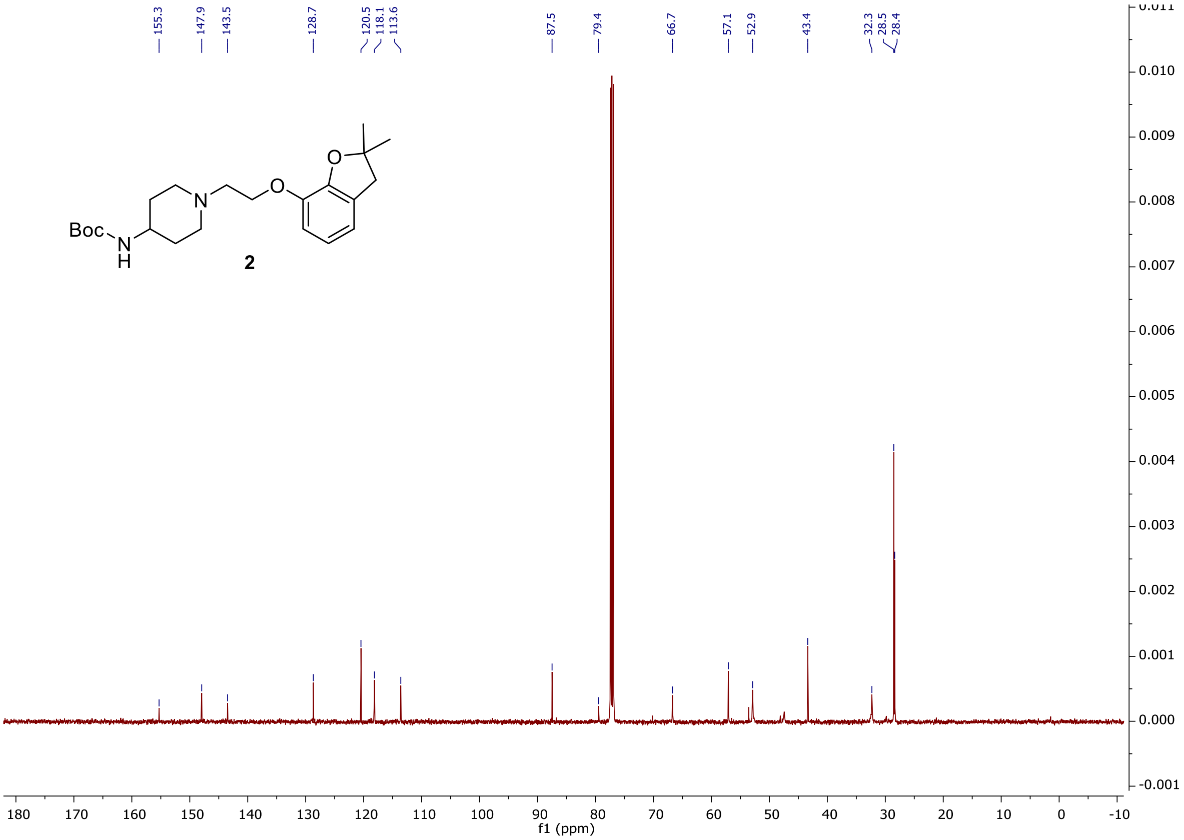


**Figure S3.** $^{13}$C-NMR spectra (125 MHz, $CDCl_3$) for *tert*-butyl {1-[2-(2,2-dimethyl-2,3-dihydrobenzofuran-7-yloxy)ethyl]piperidin-4-yl}carbamate (**2**).

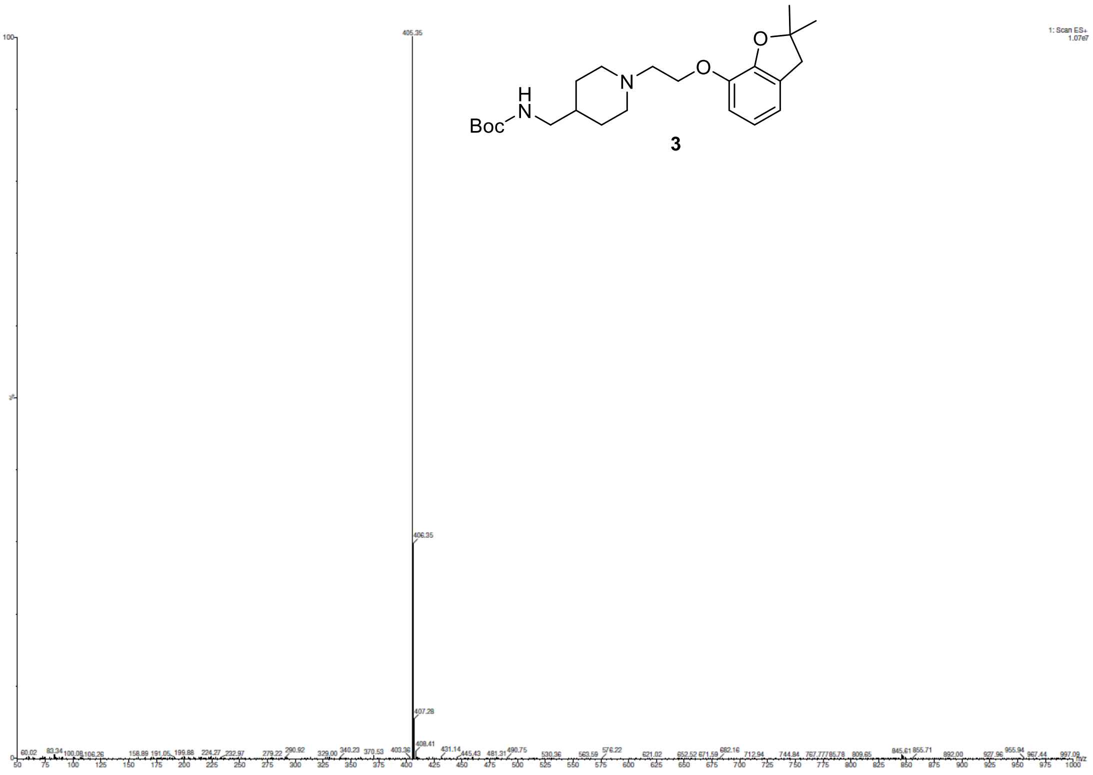


**Figure S4.** MS spectra for tert-butyl ({1-[2-(2,2-dimethyl-2,3-dihydrobenzofuran-7-yloxy)ethyl]piperidin-4-yl}methyl)carbamate (**3**).

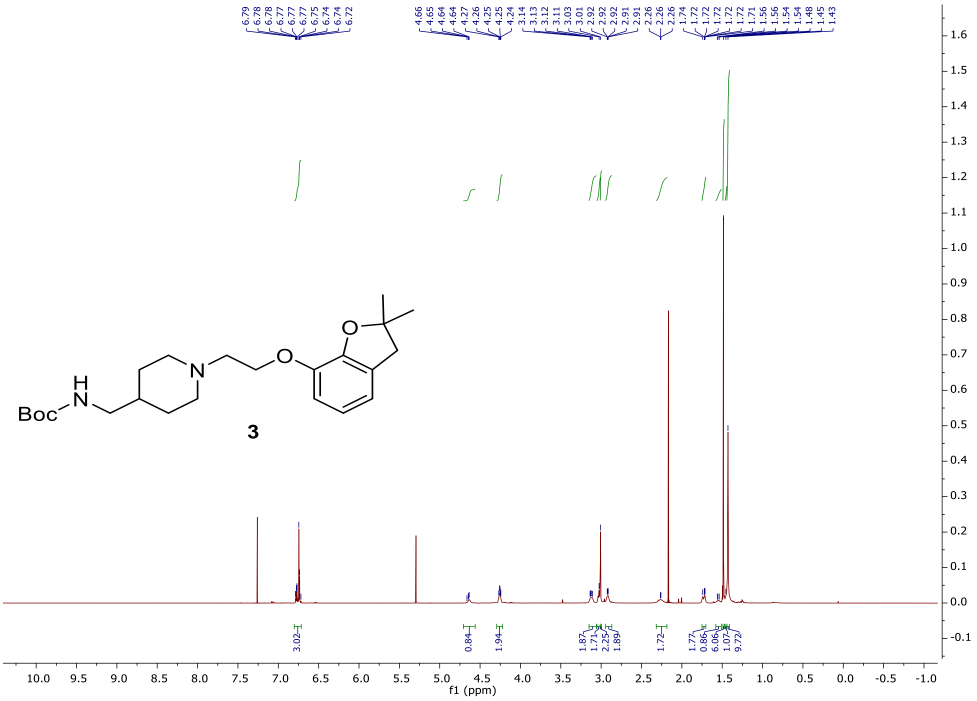


**Figure S5.** $^{1}H$-NMR spectra (500 MHz, $CDCl_3$) for *tert*-butyl ({1-[2-(2,2-dimethyl-2,3-dihydrobenzo-furan-7-yloxy)ethyl]piperidin-4-yl}methyl)carbamate (**3**).

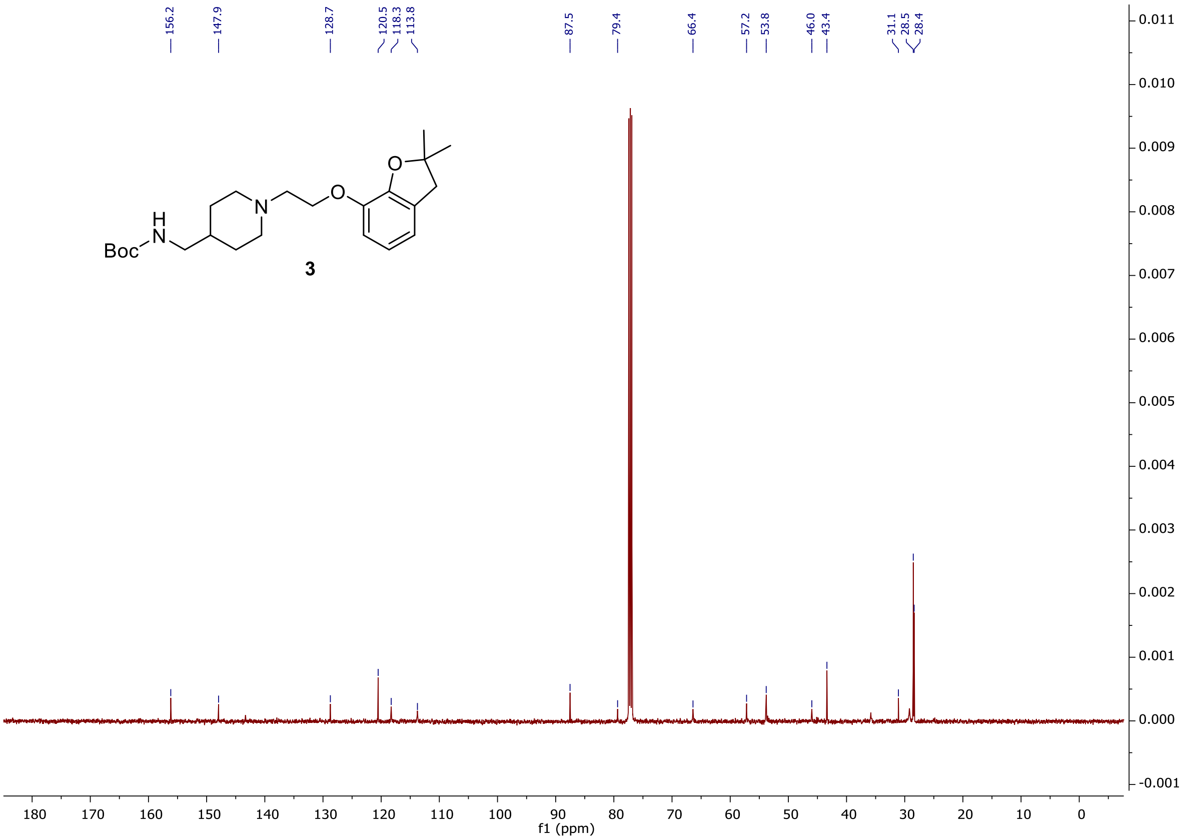


**Figure S6.** $^{13}C$-NMR spectra (125 MHz, $CDCl_3$) for *tert*-butyl ({1-[2-(2,2-dimethyl-2,3-dihydrobenzofuran-7-yloxy)ethyl]piperidin-4-yl}methyl)carbamate (**3**).

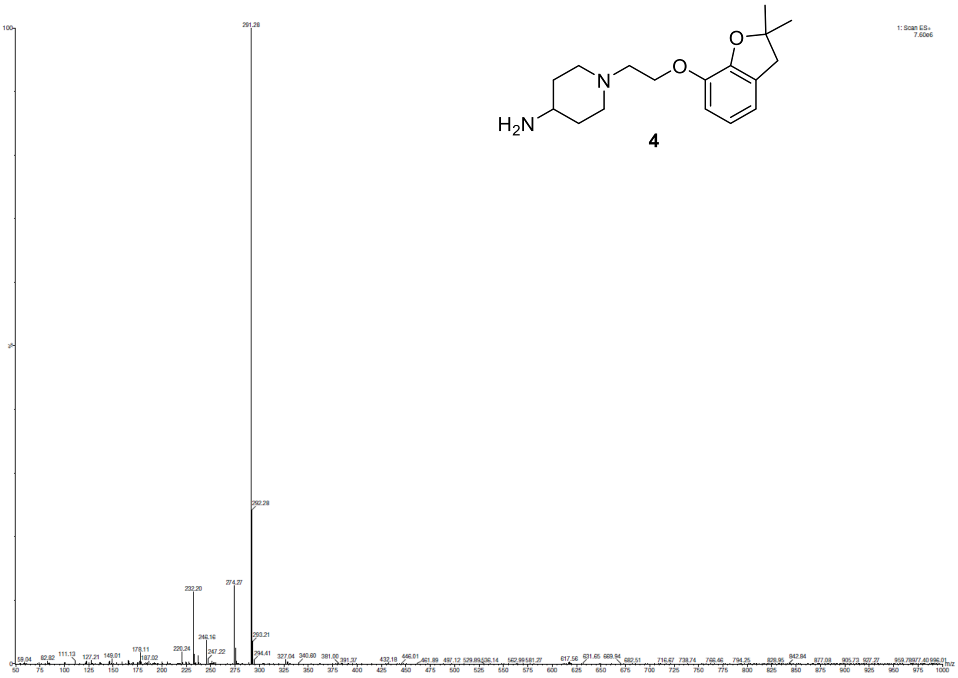

**Figure S7.** MS spectra for 1-{2-[(2,2-dimethyl-2,3-dihydrobenzofuran-7-yl)oxy]ethyl}piperidin-4-amine (**4**).

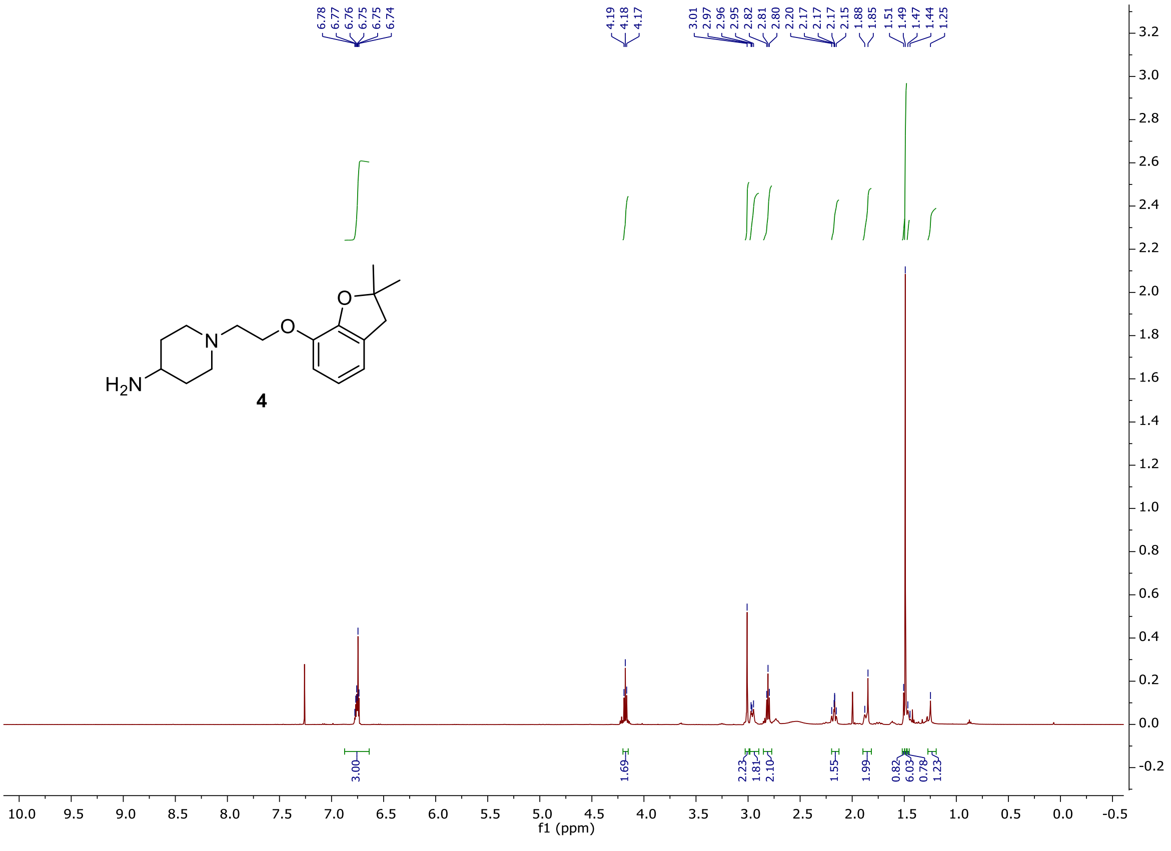

**Figure S8.** $^{1}$H-NMR spectra (500 MHz, $CDCl_3$) for 1-{2-[(2,2-dimethyl-2,3-dihydrobenzofuran-7-yl)oxy]ethyl}piperidin-4-amine (**4**).

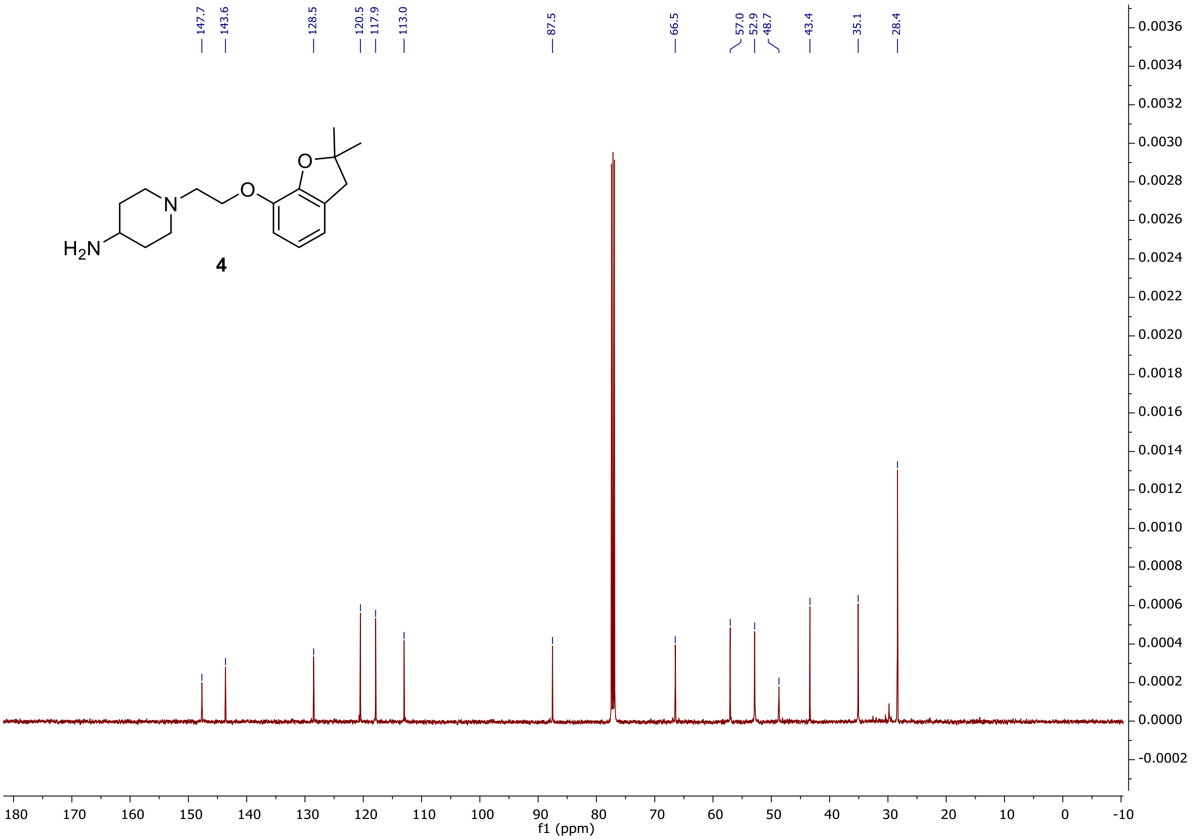


**Figure S9.** $^{13}$C-NMR spectra (125 MHz, $CDCl_3$) for 1-{2-[(2,2-dimethyl-2,3-dihydrobenzofuran-7-yl)oxy]ethyl}piperidin-4-amine (**4**).

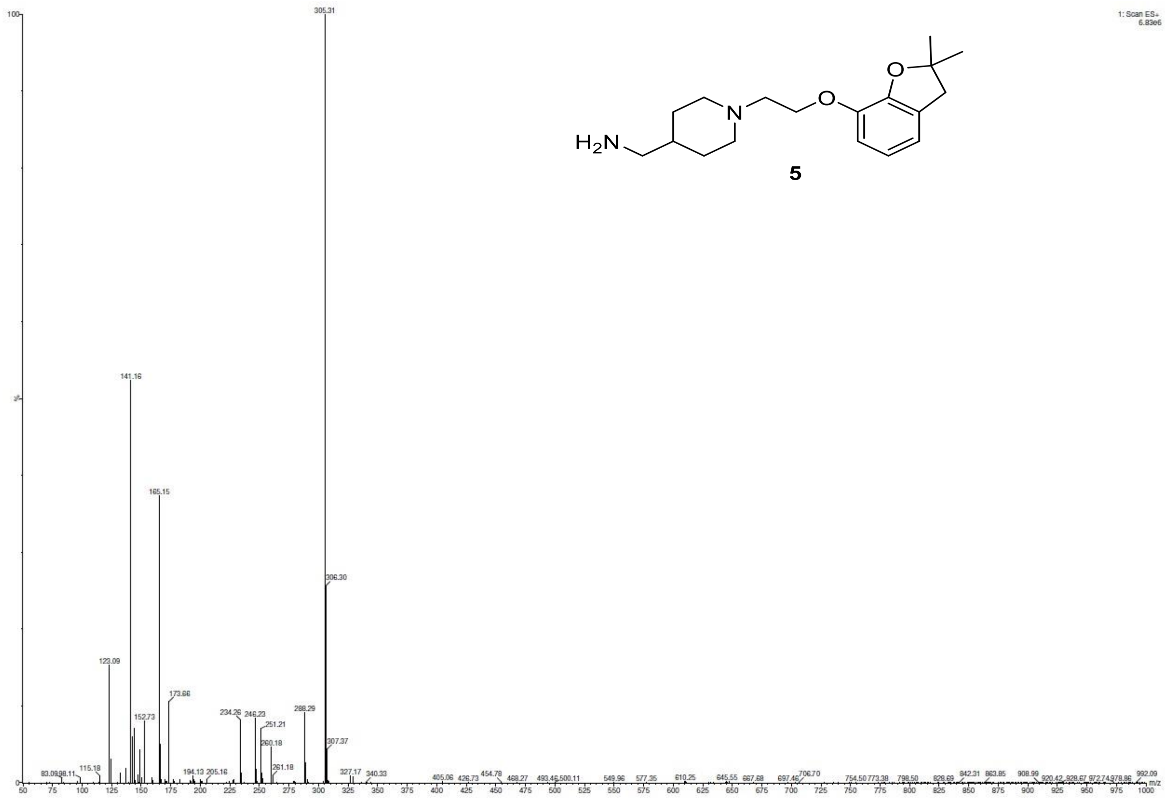


**Figure S10.** MS spectra for (1-{2-[(2,2-dimethyl-2,3-dihydrobenzofuran-7-yl)oxy]ethyl)piperidin-4-yl)methanamine (**5**).

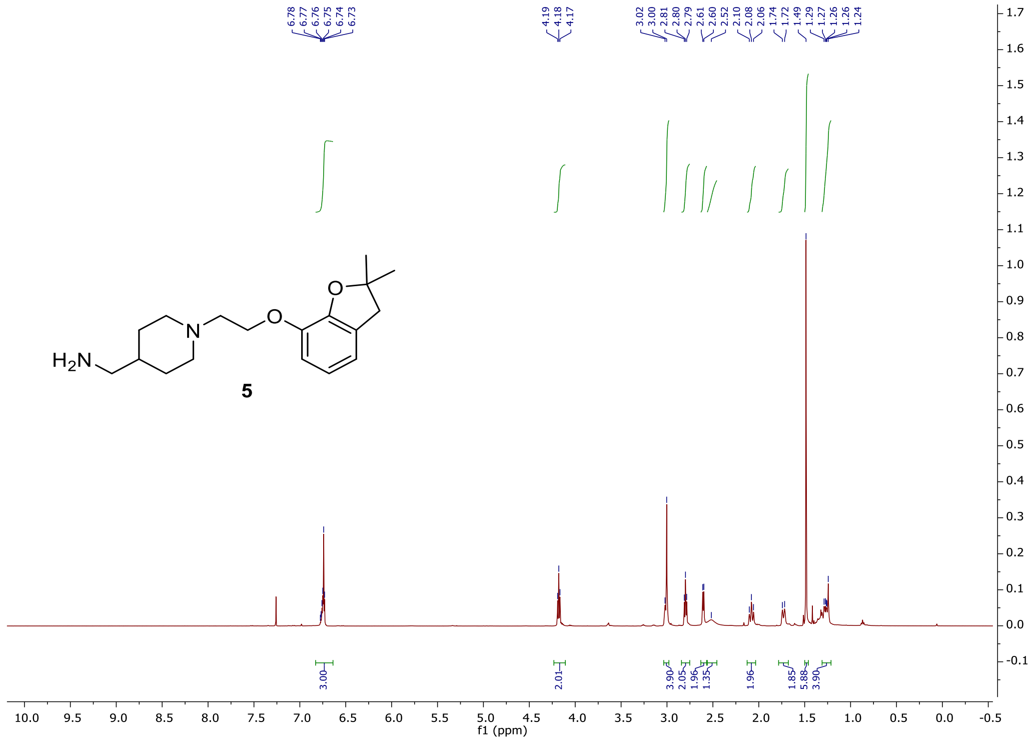

**Figure S11.** $^{1}H$-NMR spectra (500 MHz, $CDCl_3$) for (1-{2-[(2,2-dimethyl-2,3-dihydrobenzofuran-7-yl)oxy]ethyl)piperidin-4-yl)methanamine (**5**).

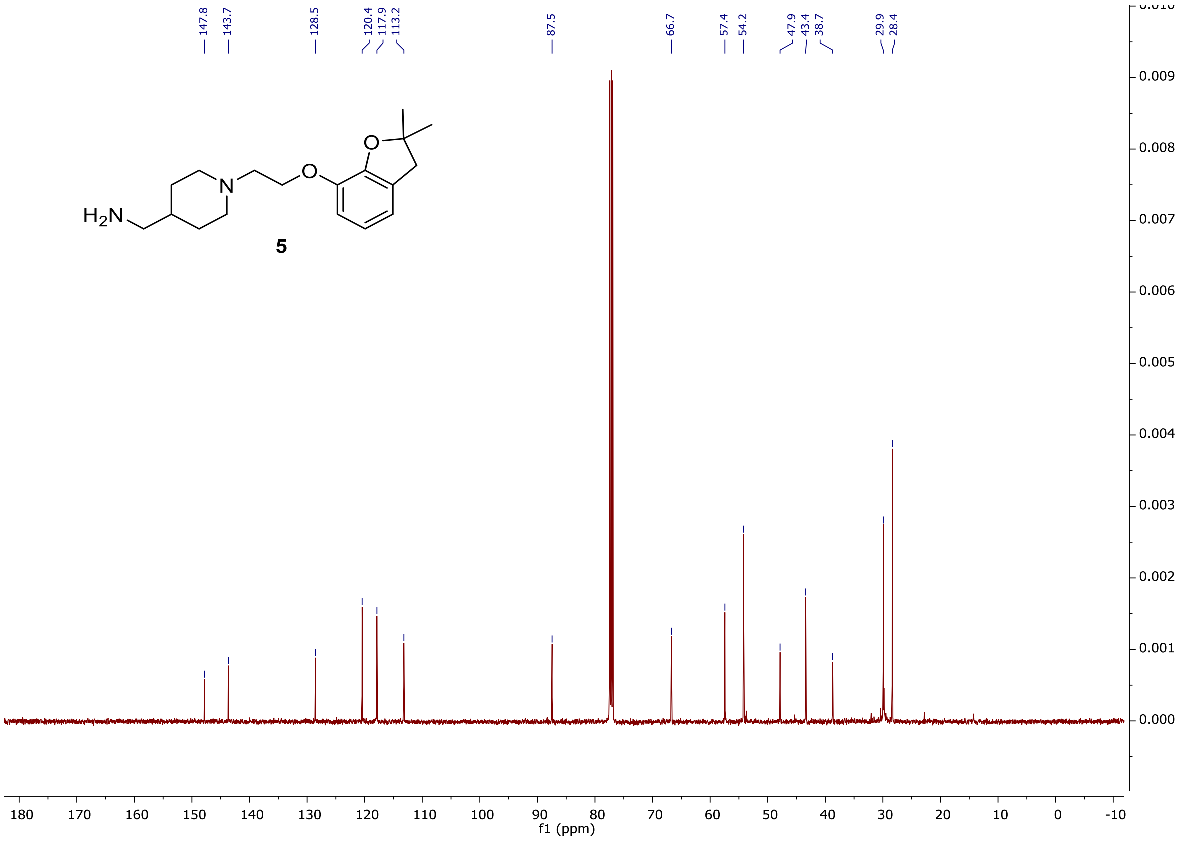

**Figure S12.** $^{13}C$-NMR spectra (125 MHz, $CDCl_3$) for (1-{2-[(2,2-dimethyl-2,3-dihydrobenzofuran-7-yl)oxy]ethyl)piperidin-4-yl)methanamine (**5**).

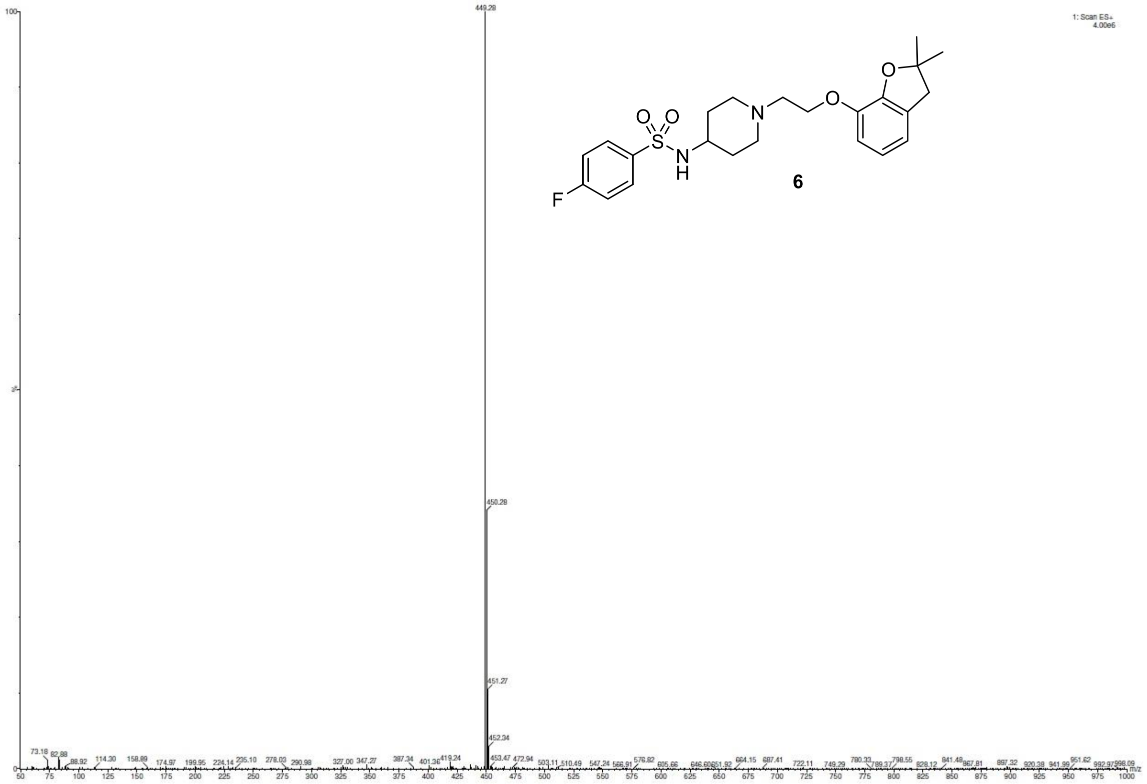

**Figure S13.** MS spectra for 4-fluoro-*N*-{1-[2-(2,2-dimethyl-2,3-dihydrobenzofuran-7-yloxy)ethyl]piperidin-4-yl}benzenesulfonamide (**6**).

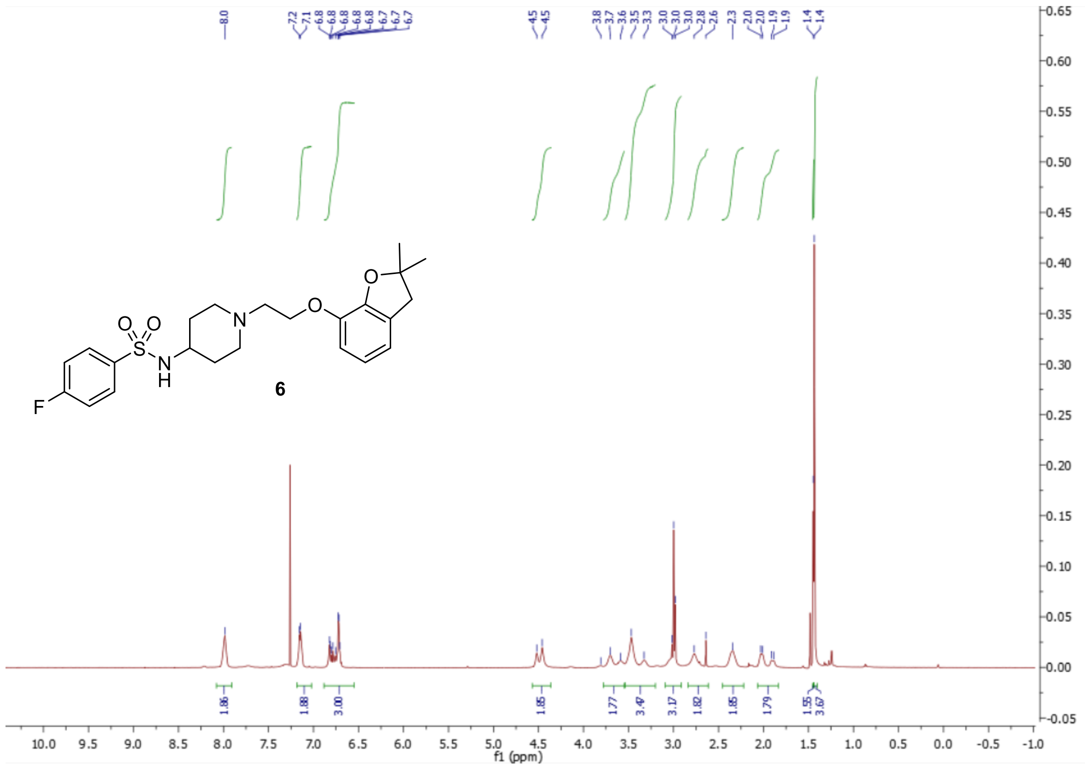

**Figure S14.** $^{1}$H-NMR spectra (500 MHz, $CDCl_3$) for 4-fluoro-*N*-{1-[2-(2,2-dimethyl-2,3-dihydrobenzofuran-7-yloxy)ethyl]piperidin-4-yl}benzenesulfonamide (**6**).

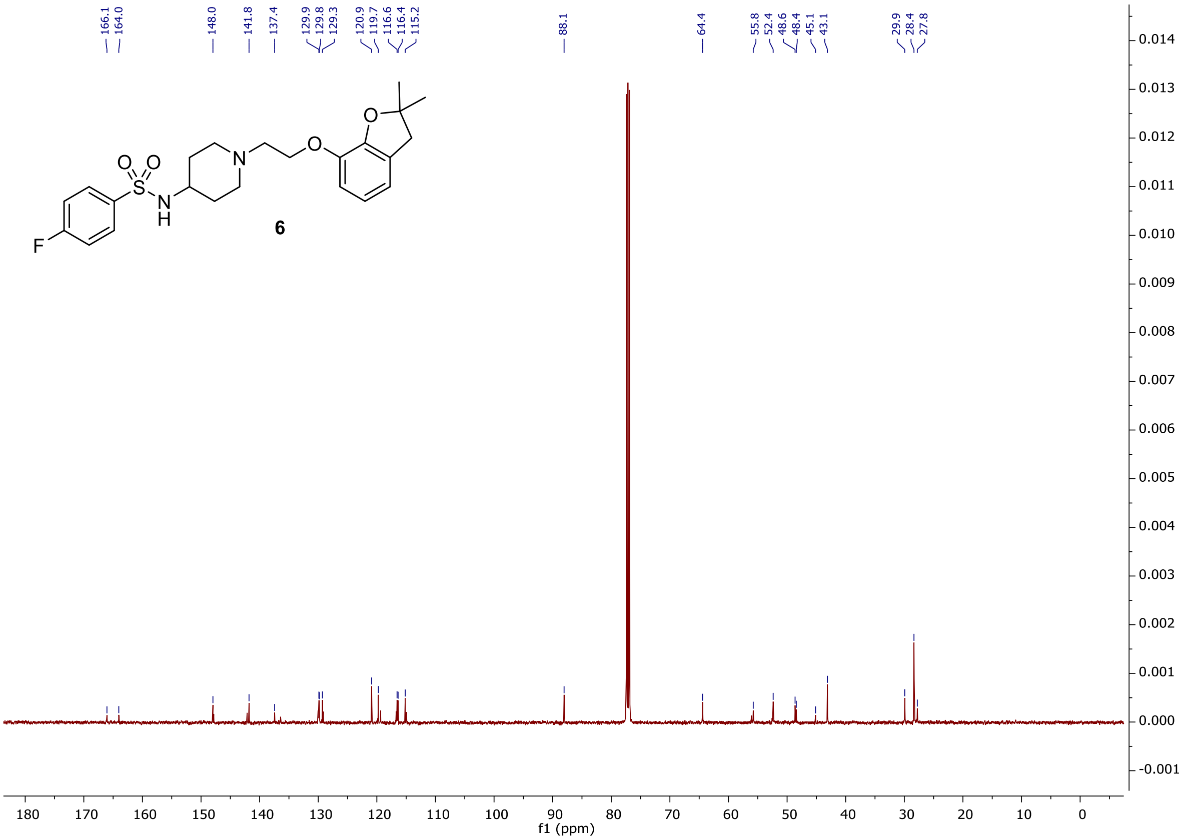


**Figure S15.** $^{13}$C-NMR spectra (125 MHz, $CDCl_3$) for 4-fluoro-*N*-{1-[2-(2,2-dimethyl-2,3-dihydrobenzofuran-7-yloxy)ethyl]piperidin-4-yl}benzenesulfonamide (**6**).

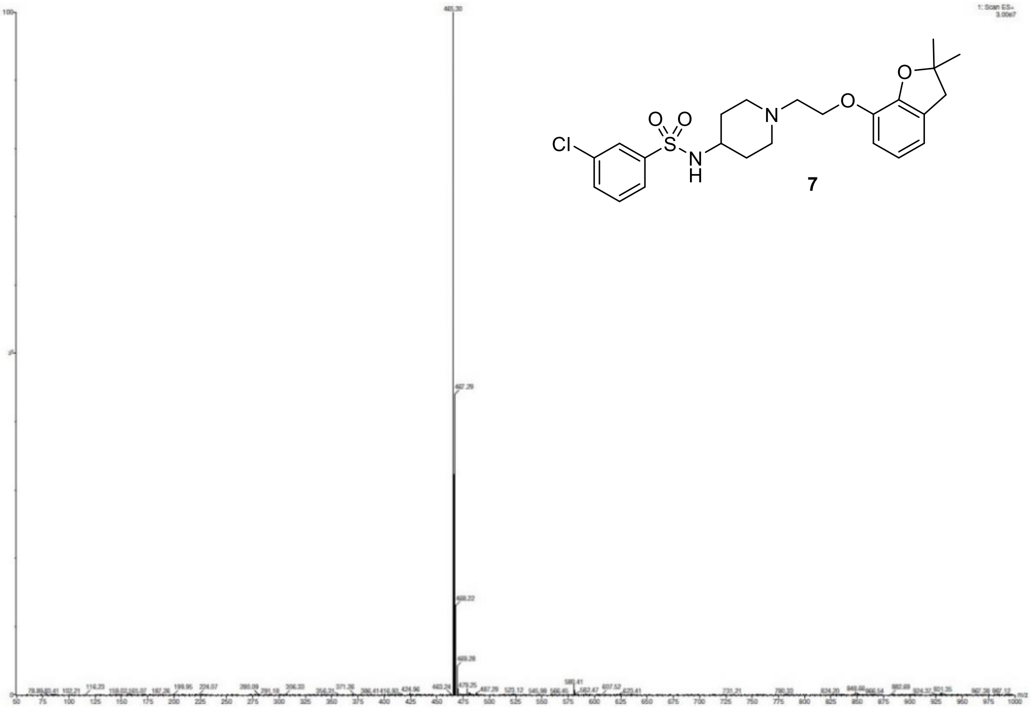


**Figure S16.** MS spectra for 3-chloro-*N*-{1-[2-(2,2-dimethyl-2,3-dihydrobenzofuran-7-yloxy)ethyl]piperidin-4-yl} benzenesulfonamide (**7**).

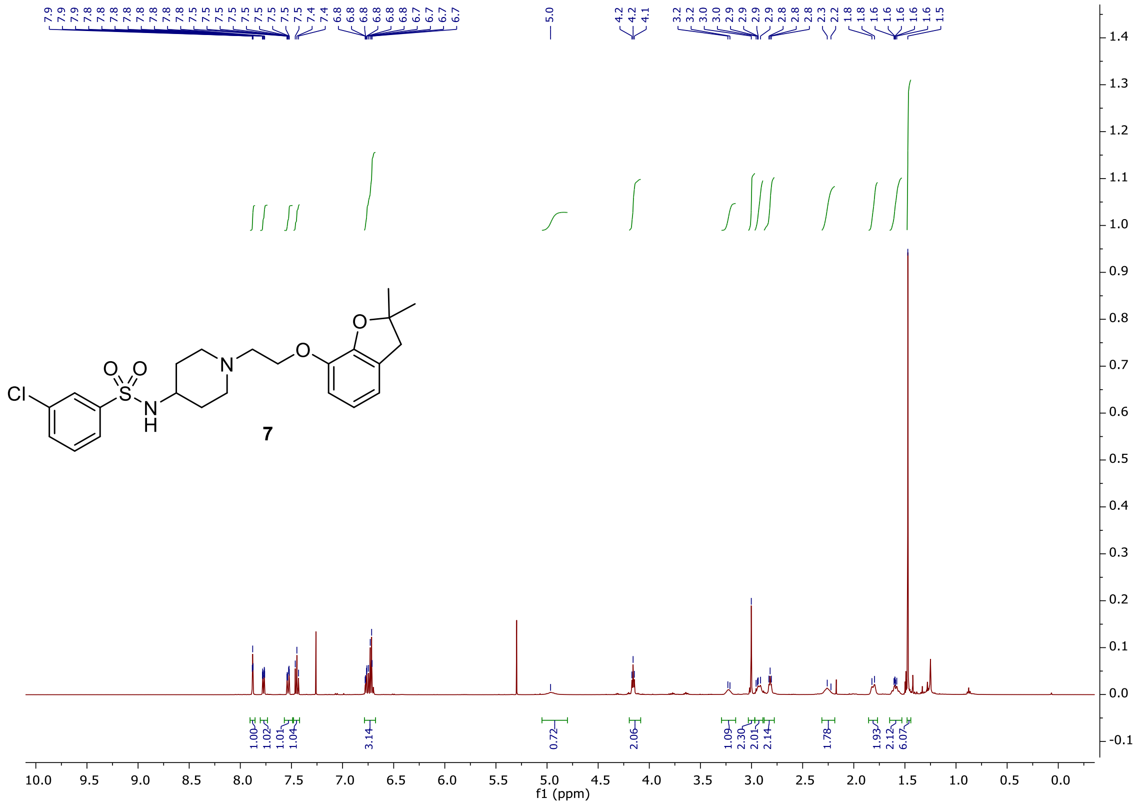

**Figure S17.** $^{1}$H-NMR spectra (500 MHz, $CDCl_3$) for 3-chloro-*N*-{1-[2-(2,2-dimethyl-2,3-dihydrobenzofuran-7-yloxy)ethyl]piperidin-4-yl} benzenesulfonamide (**7**).

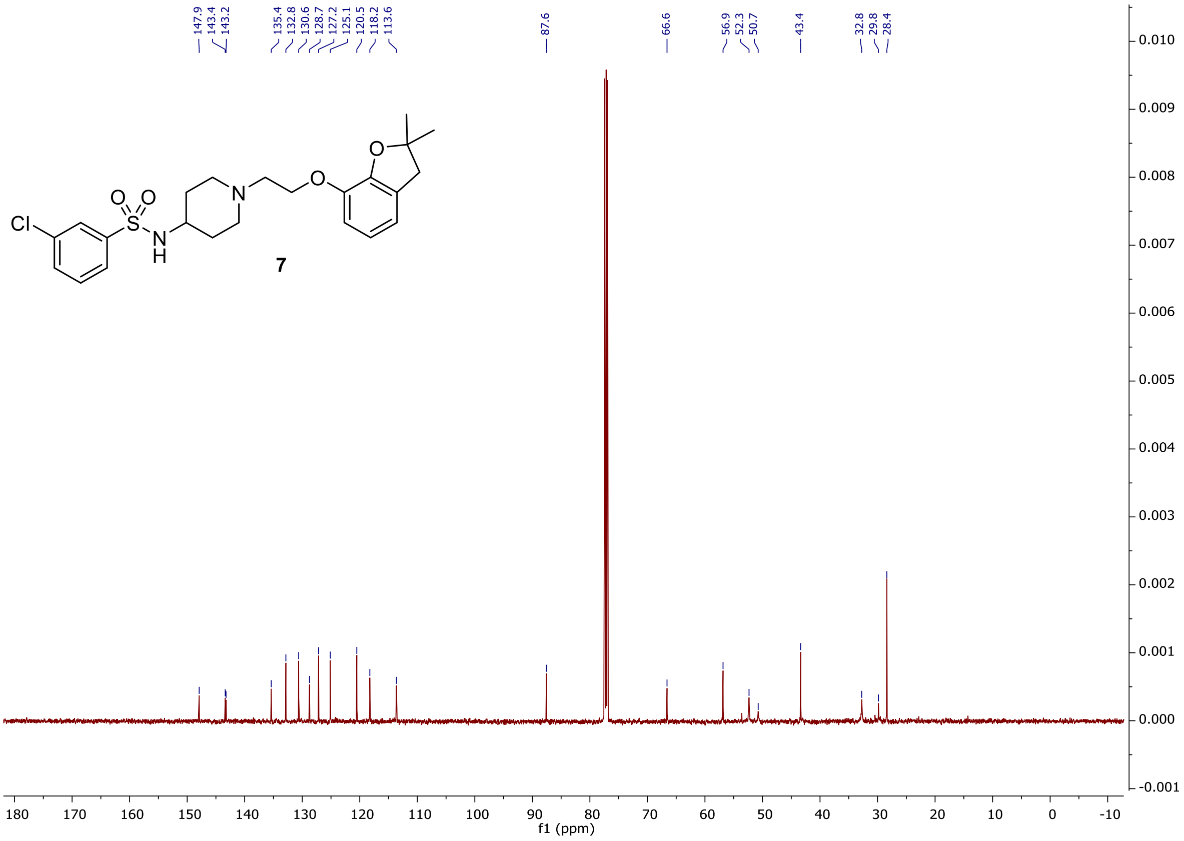

**Figure S18.** $^{13}$C-NMR spectra (125 MHz, $CDCl_3$) for 3-chloro-*N*-{1-[2-(2,2-dimethyl-2,3-dihydrobenzofuran-7-yloxy)ethyl]piperidin-4-yl} benzenesulfonamide (**7**).

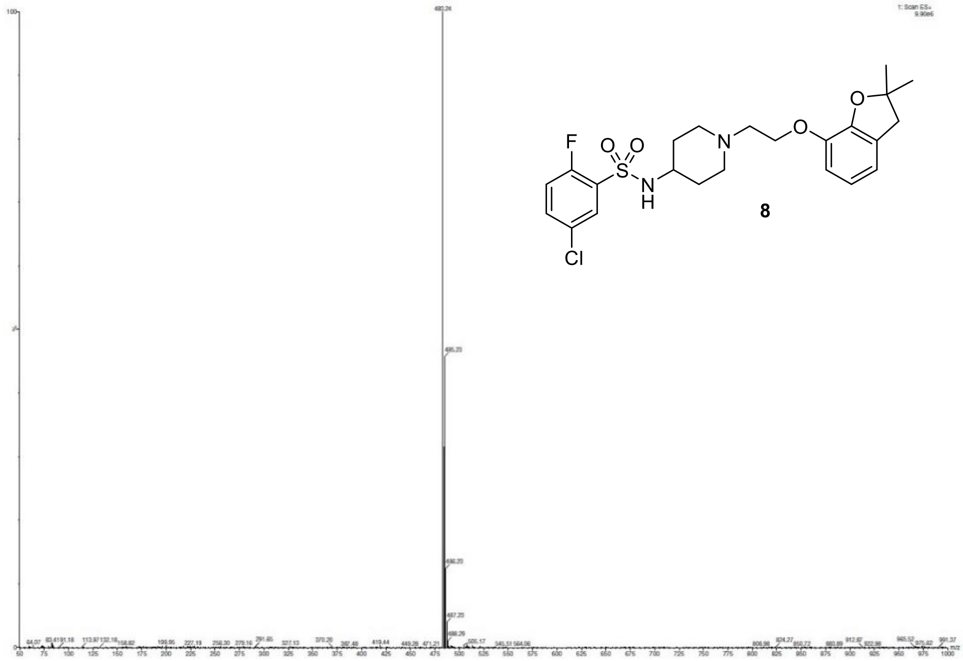


**Figure S19.** MS spectra for 5-chloro-2-fluoro-*N*-{1-[2-(2,2-dimethyl-2,3-dihydrobenzofuran-7-yloxy)ethyl]piperidin-4-yl}benzenesulfonamide (**8**).

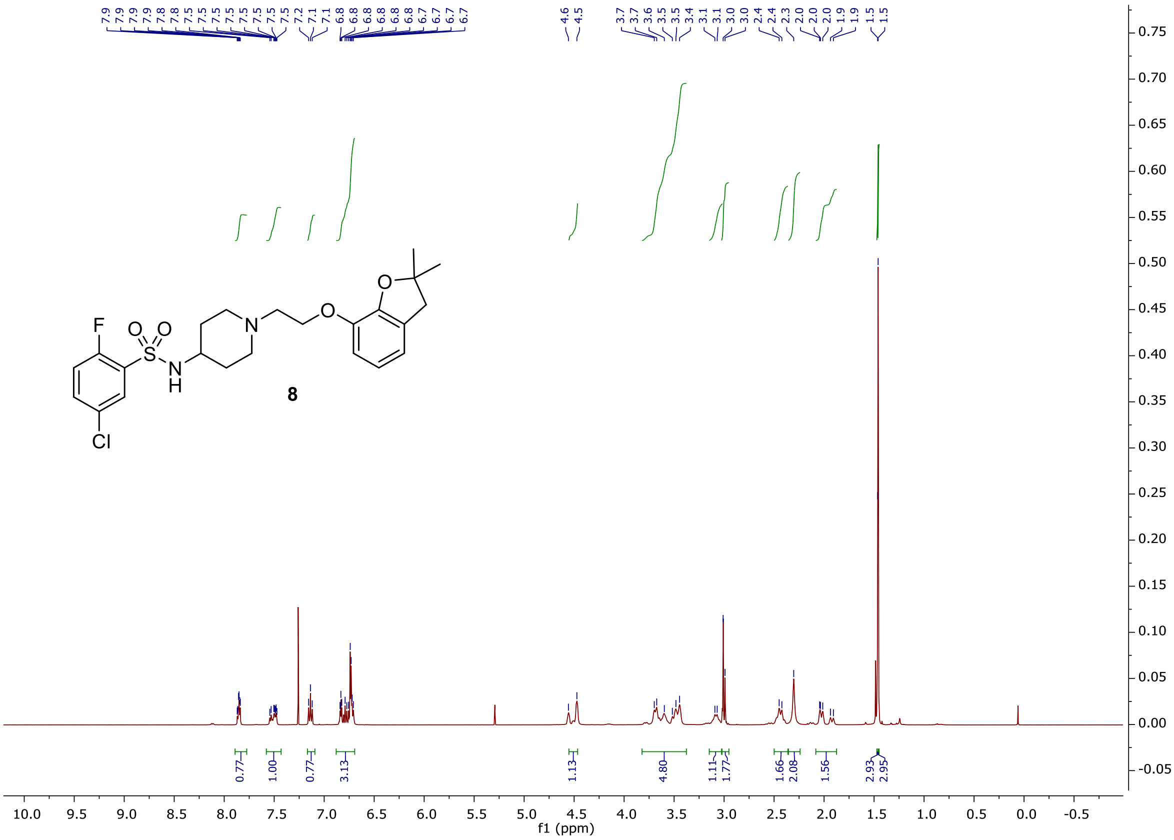


**Figure S20.** $^{1}$H-NMR spectra (500 MHz, $CDCl_3$) for 5-chloro-2-fluoro-*N*-{1-[2-(2,2-dimethyl-2,3-dihydrobenzofuran-7-yloxy)ethyl]piperidin-4-yl}benzenesulfonamide (**8**).

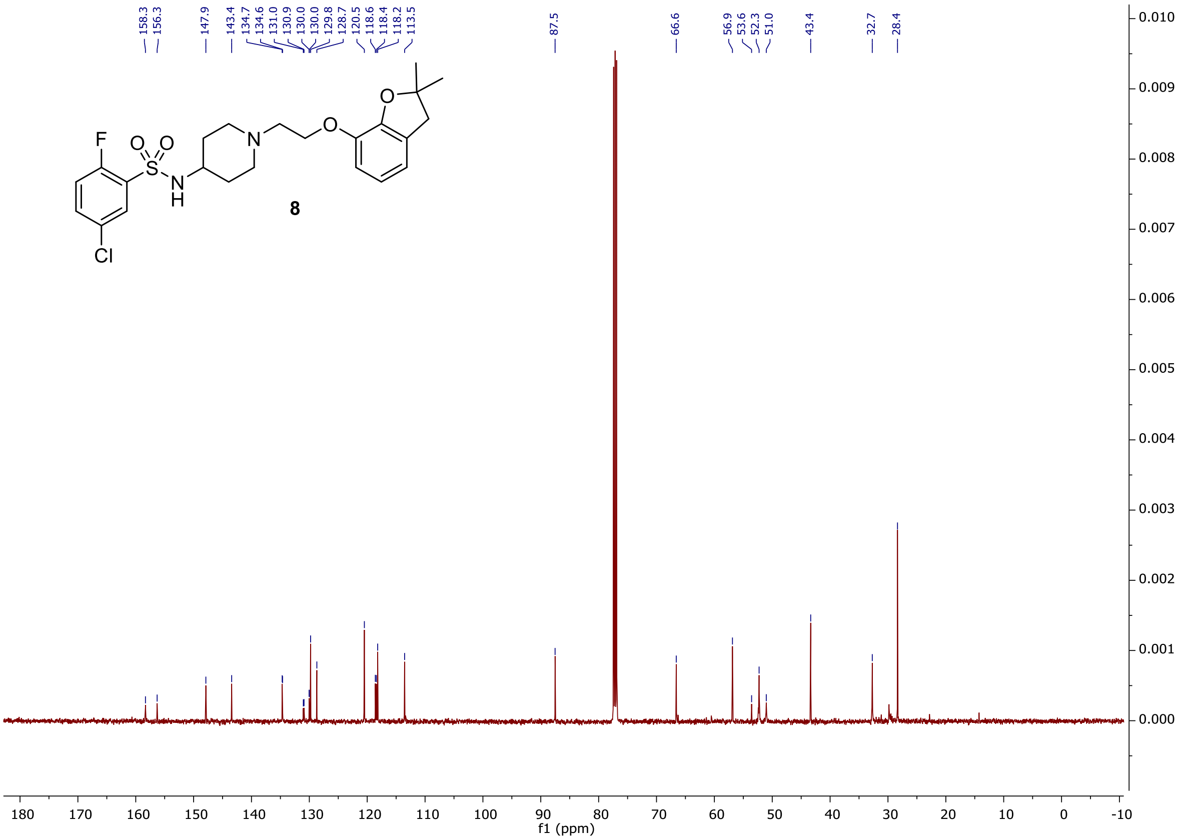


**Figure S21.** $^{13}$C-NMR spectra (125 MHz, $CDCl_3$) for 5-chloro-2-fluoro-*N*-{1-[2-(2,2-dimethyl-2,3-dihydrobenzofuran-7-yloxy)ethyl]piperidin-4-yl}benzenesulfonamide (**8**).

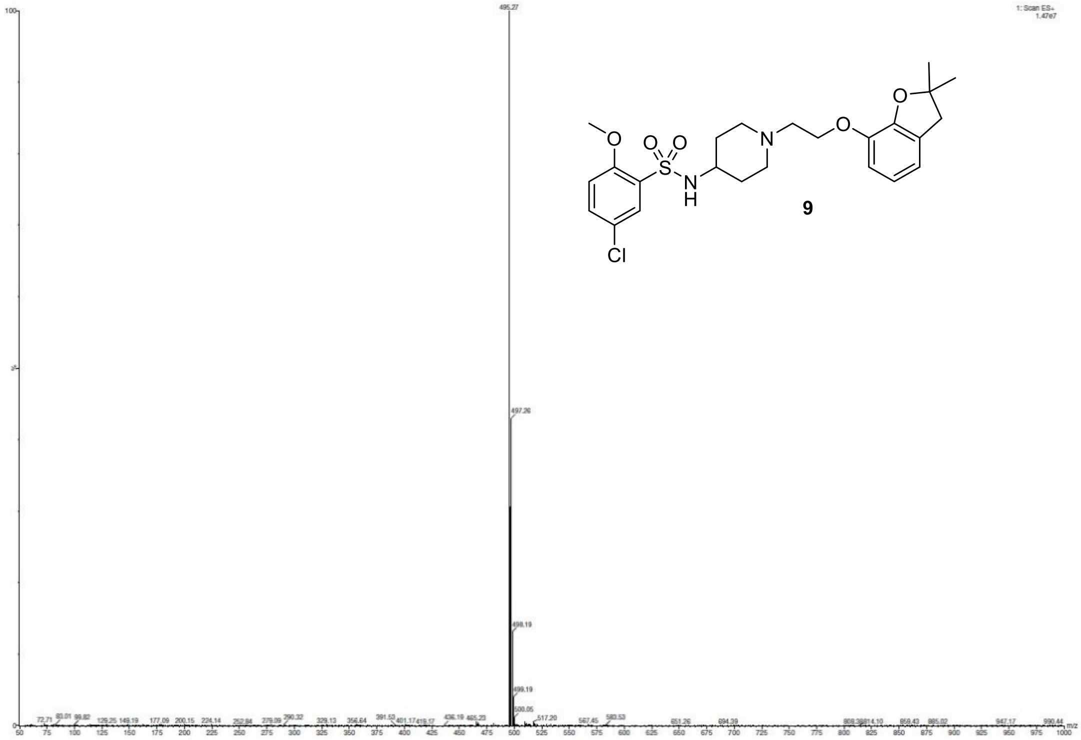


**Figure S22.** MS spectra for 5-chloro-2-methoxy-*N*-{1-[2-(2,2-dimethyl-2,3-dihydrobenzofuran-7-yloxy)ethyl] piperidin-4-yl}benzenesulfonamide (**9**).

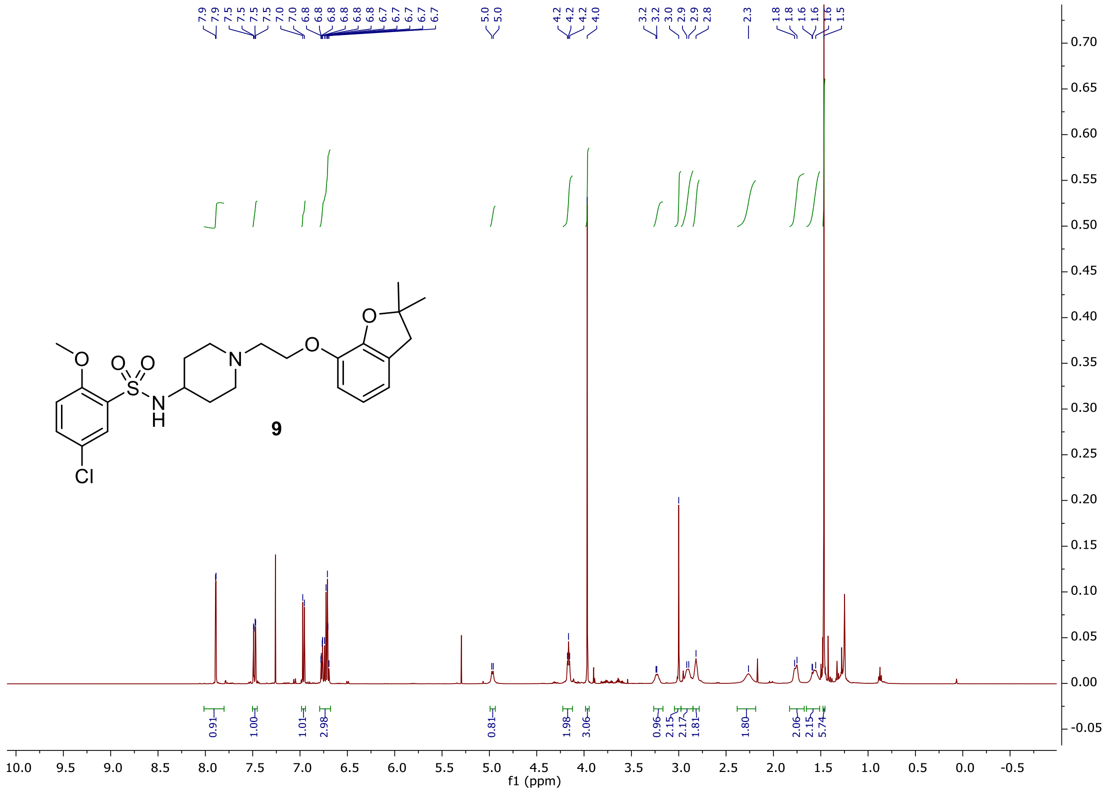


**Figure S23.** $^{1}$H-NMR spectra (500 MHz, $CDCl_3$) for 5-chloro-2-methoxy-*N*-{1-[2-(2,2-dimethyl-2,3-dihydrobenzofuran-7-yloxy)ethyl]piperidin-4-yl}benzenesulfonamide (**9**).

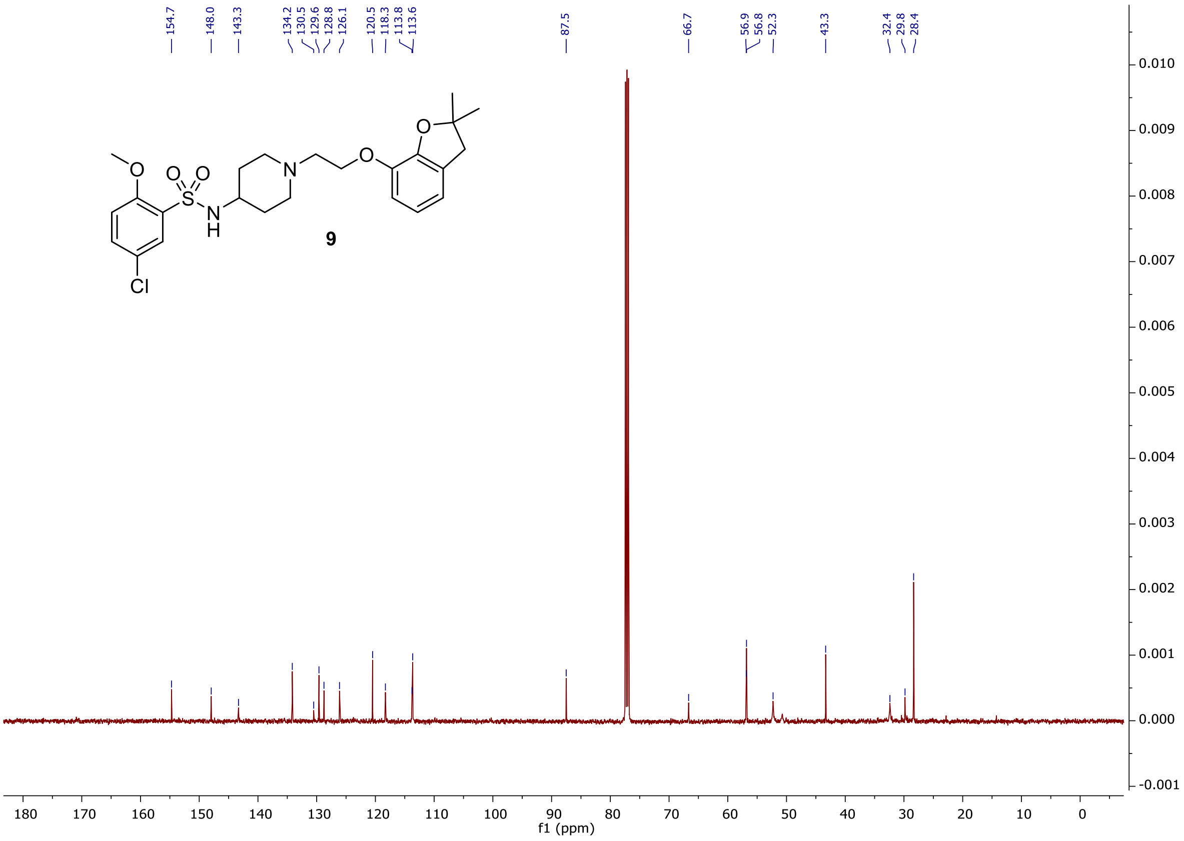


**Figure S24.** $^{13}$C-NMR spectra (125 MHz, $CDCl_3$) for 5-chloro-2-methoxy-*N*-{1-[2-(2,2-dimethyl-2,3-dihydrobenzofuran-7-yloxy)ethyl]piperidin-4-yl}benzenesulfonamide (**9**).

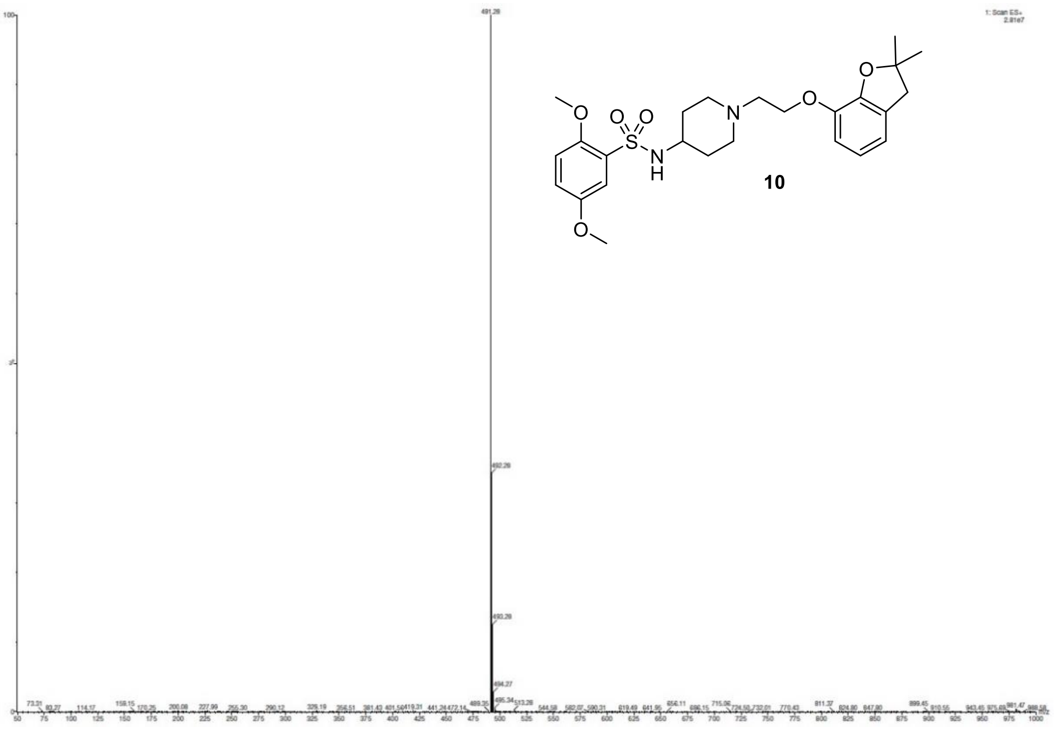


**Figure S25.** MS spectra for 2,4-dimethoxy-*N*-{1-[2-(2,2-dimethyl-2,3-dihydrobenzofuran-7-yloxy)ethy]piperidin-4-yl}benzenesulfonamide (**10**).

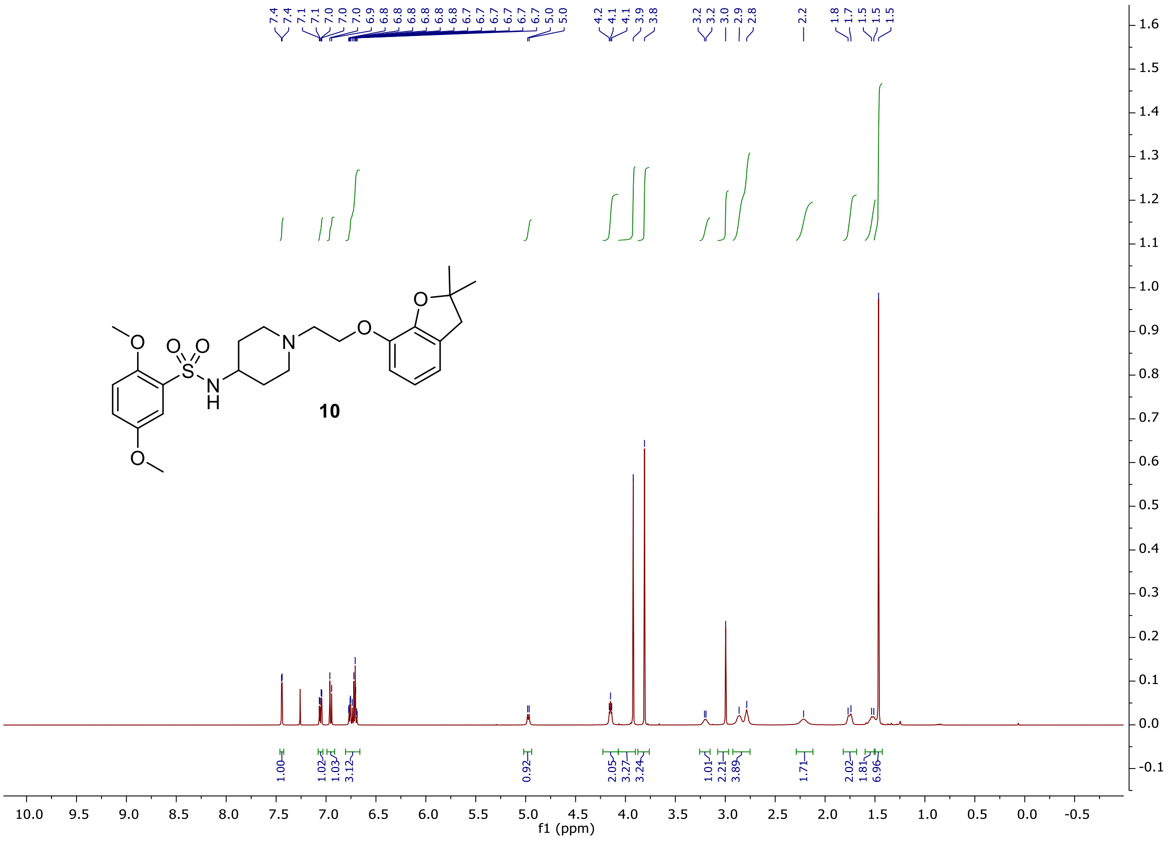


**Figure S26.** $^{1}$H-NMR spectra (500 MHz, $CDCl_3$) for 2,4-dimethoxy-*N*-{1-[2-(2,2-dimethyl-2,3-dihydrobenzofuran-7-yloxy)ethy]piperidin-4-yl}benzenesulfonamide (**10**).

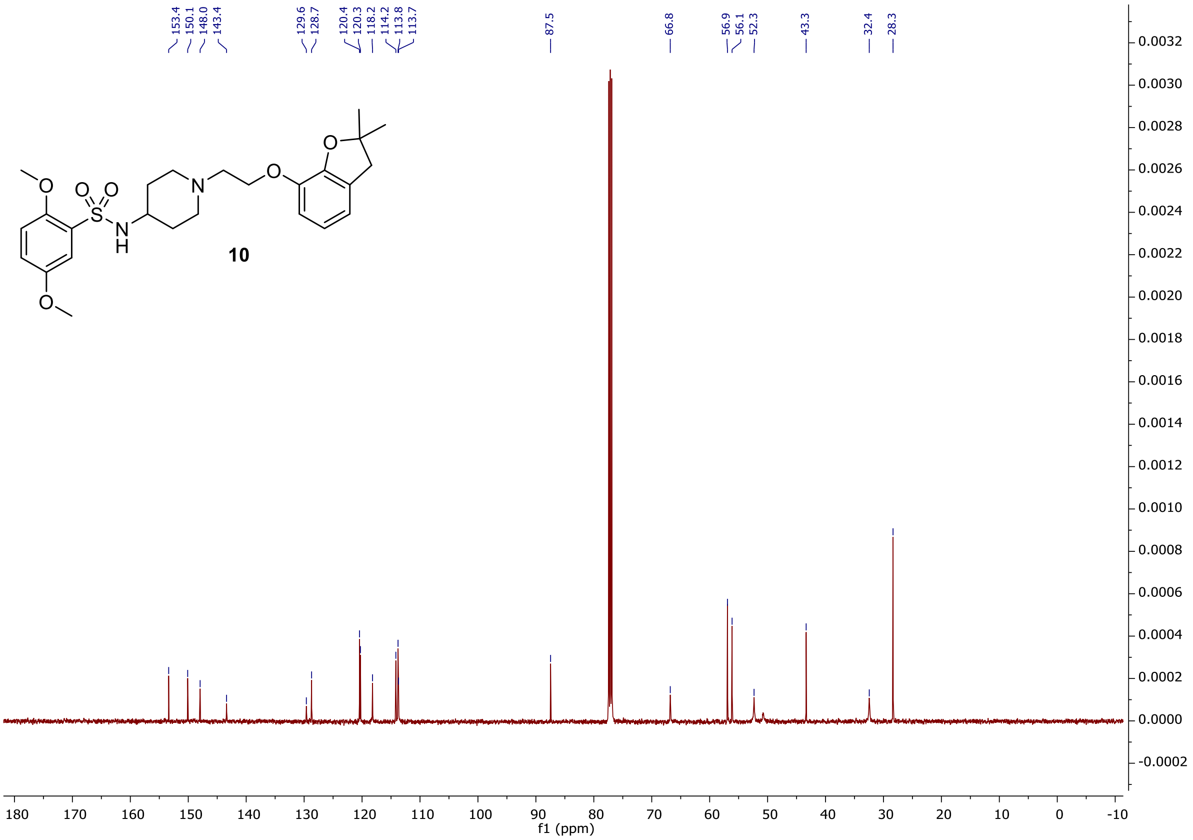


**Figure S27.** $^{13}$C-NMR spectra (125 MHz, $CDCl_3$) for 2,4-dimethoxy-*N*-{1-[2-(2,2-dimethyl-2,3-dihydrobenzofuran-7-yloxy)ethy]piperidin-4-yl}benzenesulfonamide (**10**).

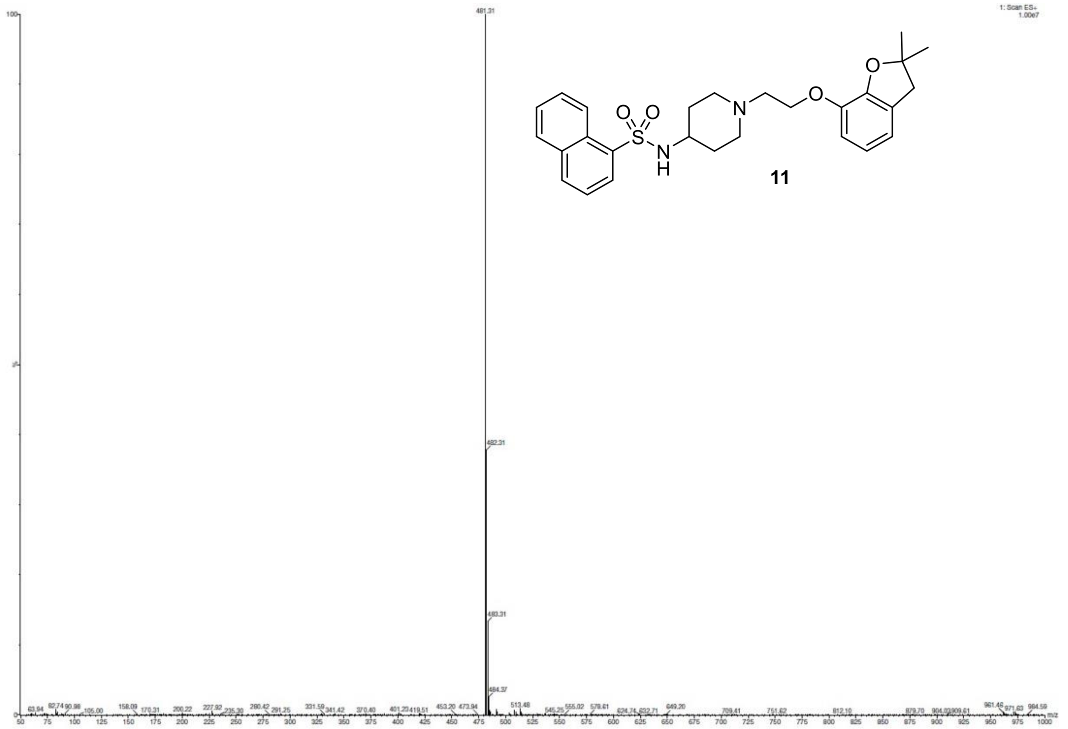


**Figure S28.** MS spectra for 1-naphthalene-*N*-{1-[2-(2,2-dimethyl-2,3-dihydrobenzofuran-7-yloxy)ethyl]piperidin-4-yl}sulfonamide (**11**).

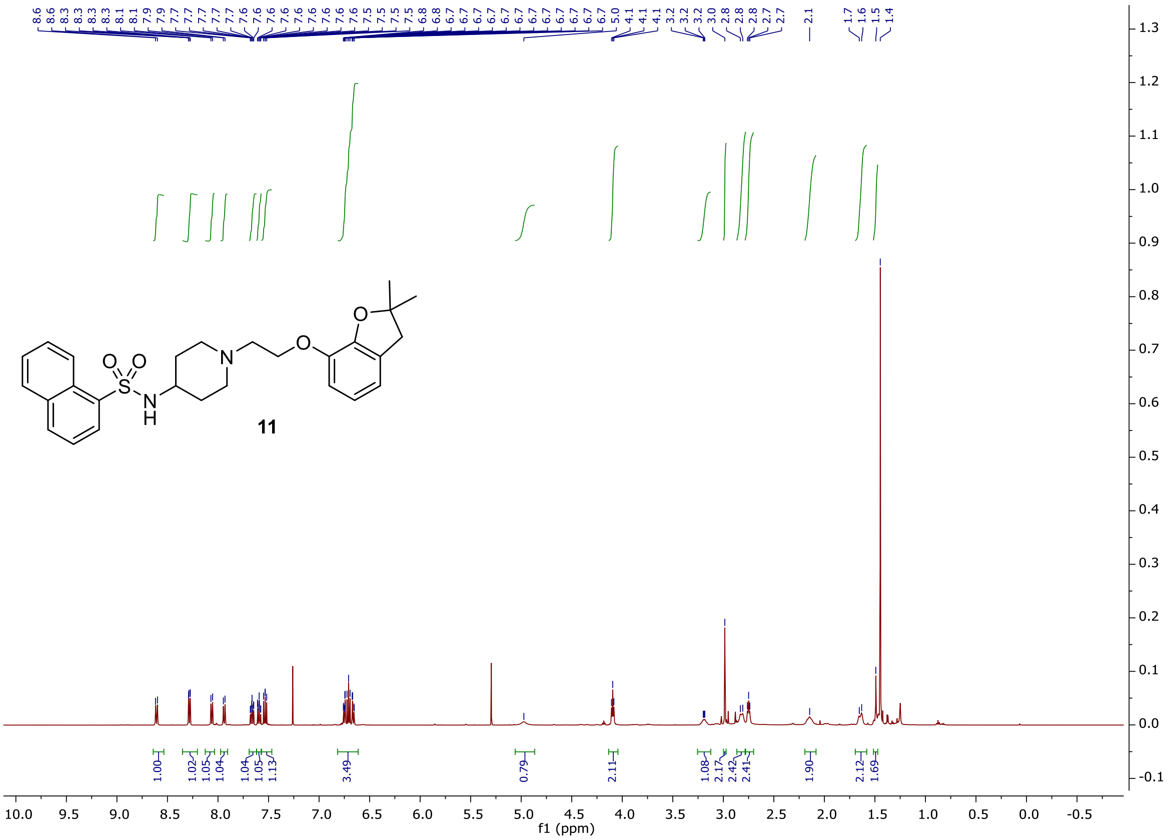


**Figure S29.** $^1$H-NMR spectra (500 MHz, $CDCl_3$) for 1-naphthalene-*N*-{1-[2-(2,2-dimethyl-2,3-dihydrobenzofuran-7-yloxy)ethyl]piperidin-4-yl}sulfonamide (**11**).

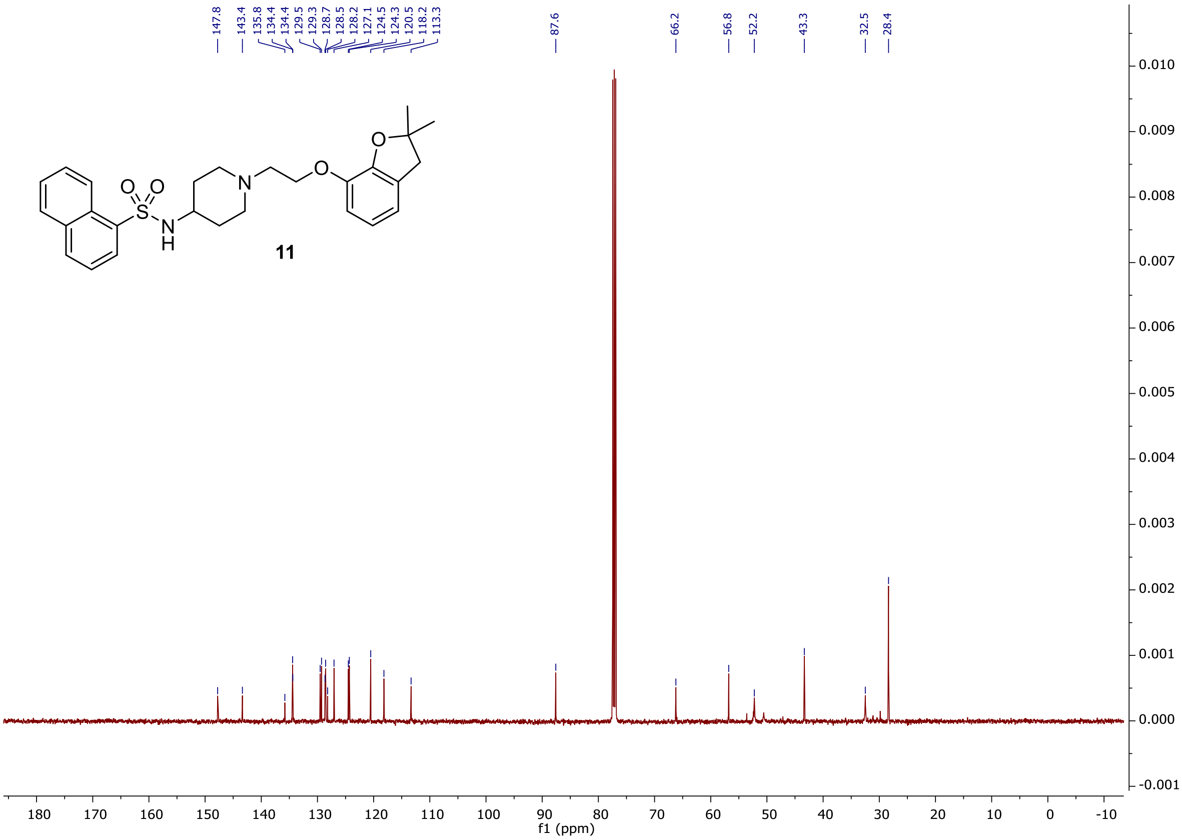


**Figure S30.** $^{13}C$-NMR spectra (125 MHz, $CDCl_3$) for 1-naphthalene-*N*-{1-[2-(2,2-dimethyl-2,3-dihydrobenzofuran-7-yloxy)ethyl]piperidin-4-yl}sulfonamide (**11**).

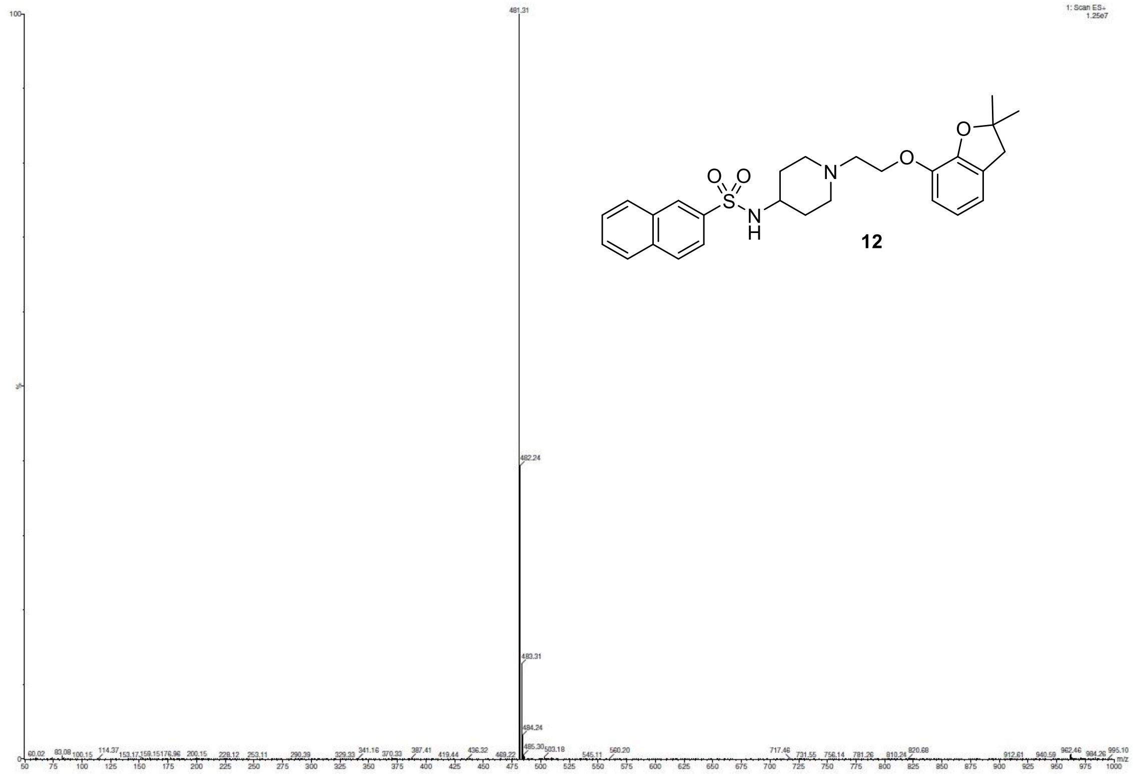


**Figure S31.** MS spectra for 2-naphthalene-*N*-{1-[2-(2,2-dimethyl-2,3-dihydrobenzofuran-7-yloxy)ethyl]piperidin-4-yl}sulfonamide (**12**).

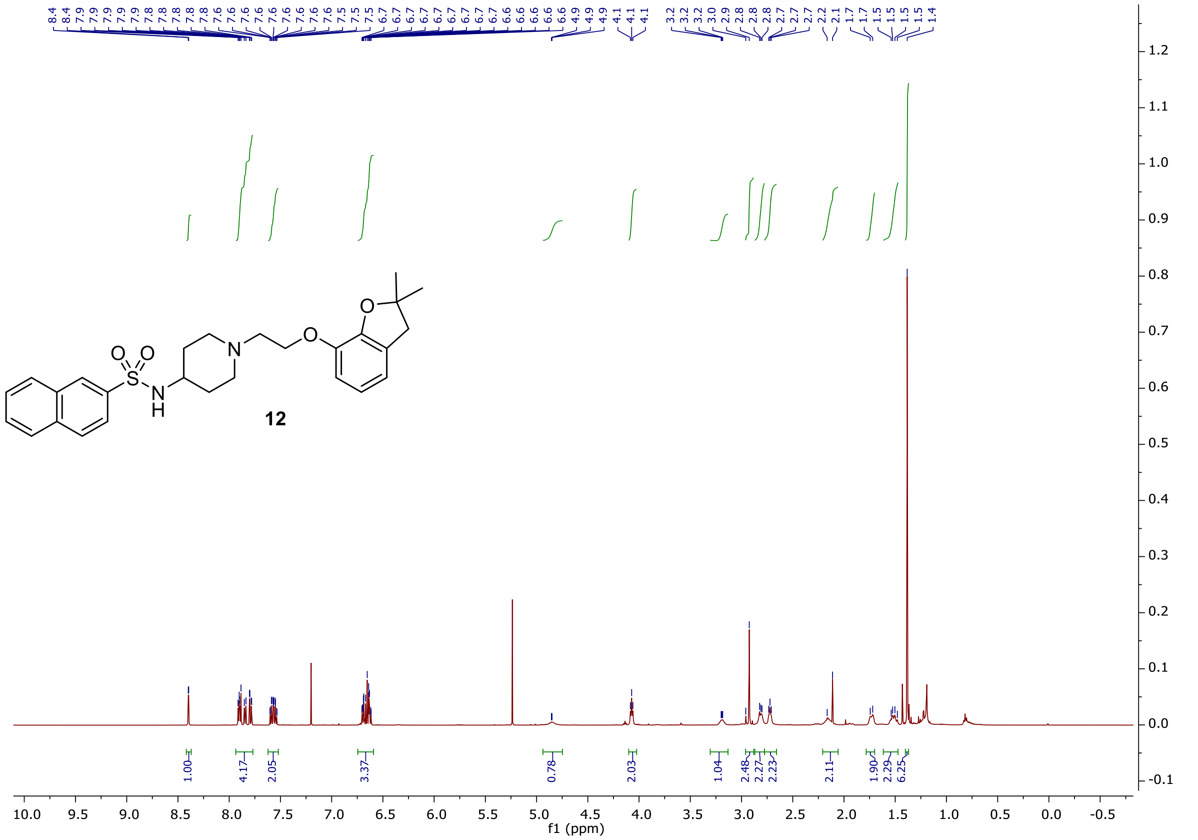

**Figure S32.** $^{1}H$-NMR spectra (500 MHz, $CDCl_3$) for 2-naphthalene-*N*-{1-[2-(2,2-dimethyl-2,3-dihydrobenzofuran-7-yloxy)ethyl]piperidin-4-yl}sulfonamide (**12**).

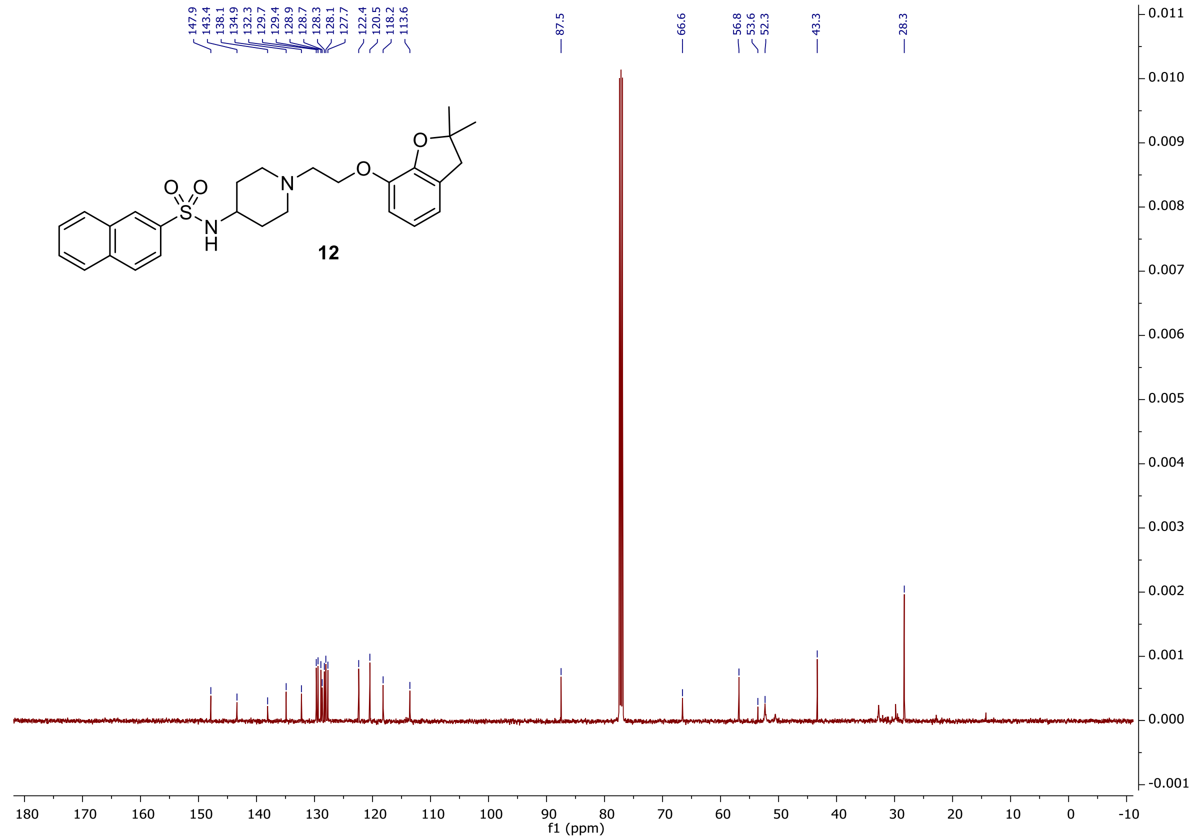

**Figure S33.** $^{13}C$-NMR spectra (125 MHz, $CDCl_3$) for 2-naphthalene-*N*-{1-[2-(2,2-dimethyl-2,3-dihydrobenzofuran-7-yloxy)ethyl]piperidin-4-yl}sulfonamide (**12**).

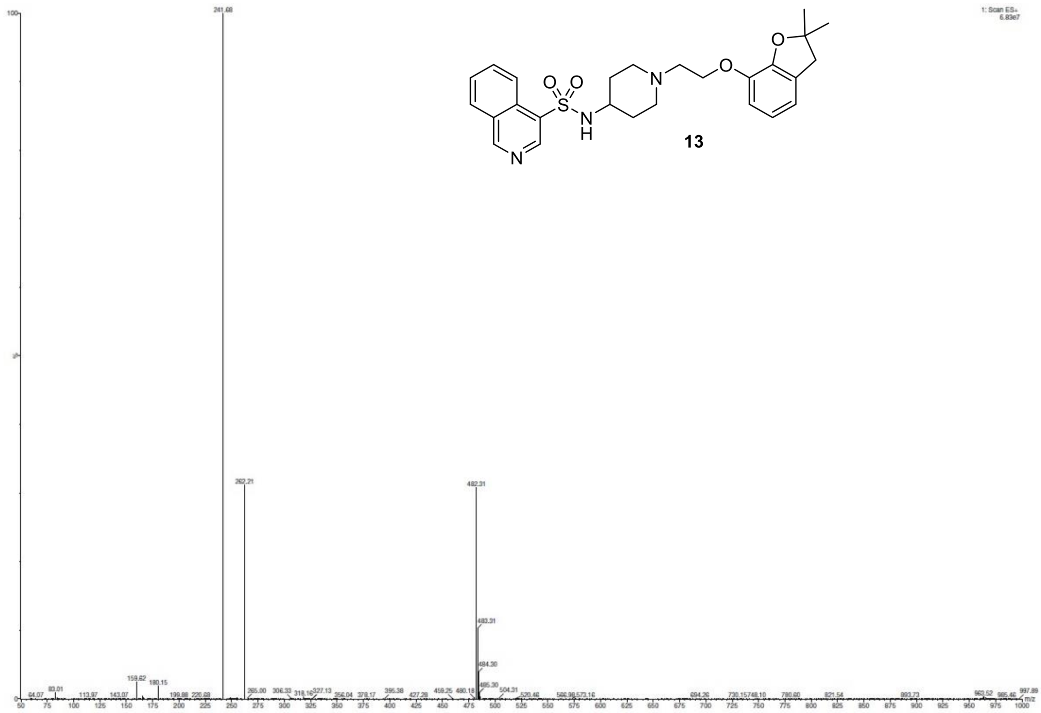

**Figure S34.** MS spectra for 4-isoquinoline-*N*-{1-[2-(2,2-dimethyl-2,3-dihydrobenzofuran-7-yloxy)ethyl]piperidin-4-yl}sulfonamide (13).

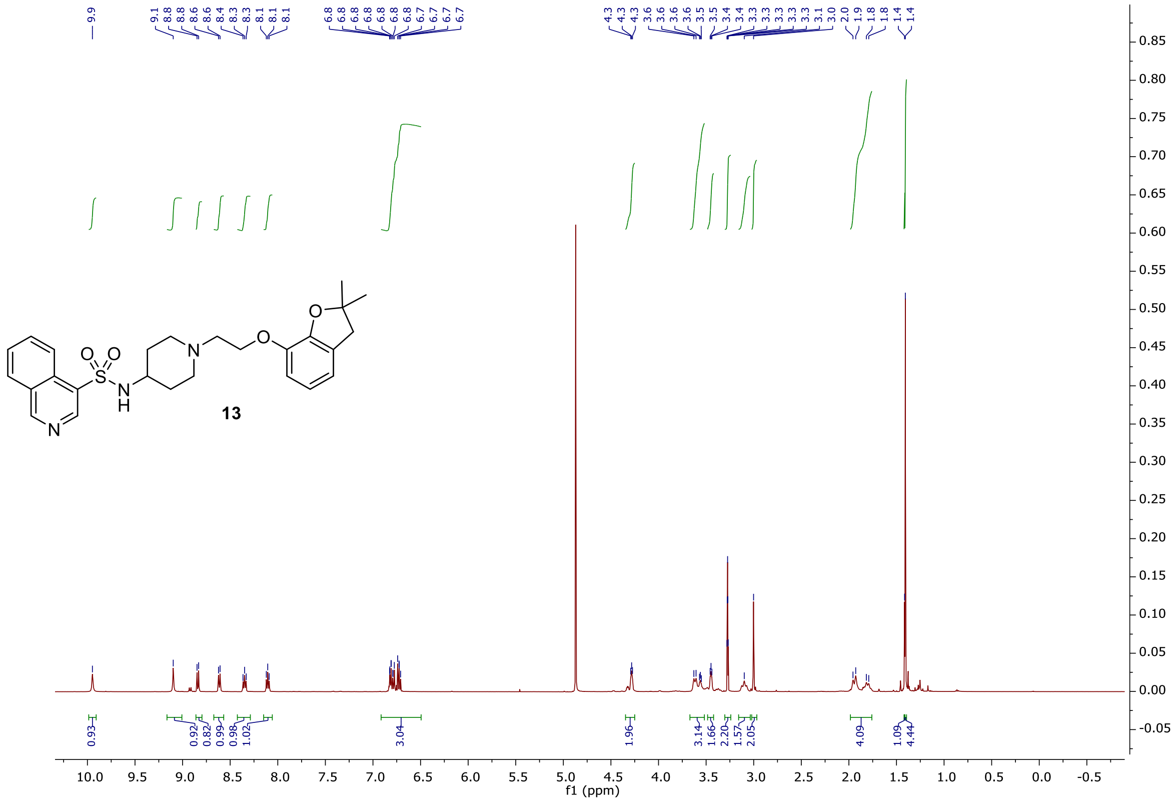

**Figure S35.** $^{1}$H-NMR spectra (500 MHz, $CDCl_3$) for 4-isoquinoline-*N*-{1-[2-(2,2-dimethyl-2,3-dihydrobenzofuran-7-yloxy)ethyl]piperidin-4-yl}sulfonamide (**13**).

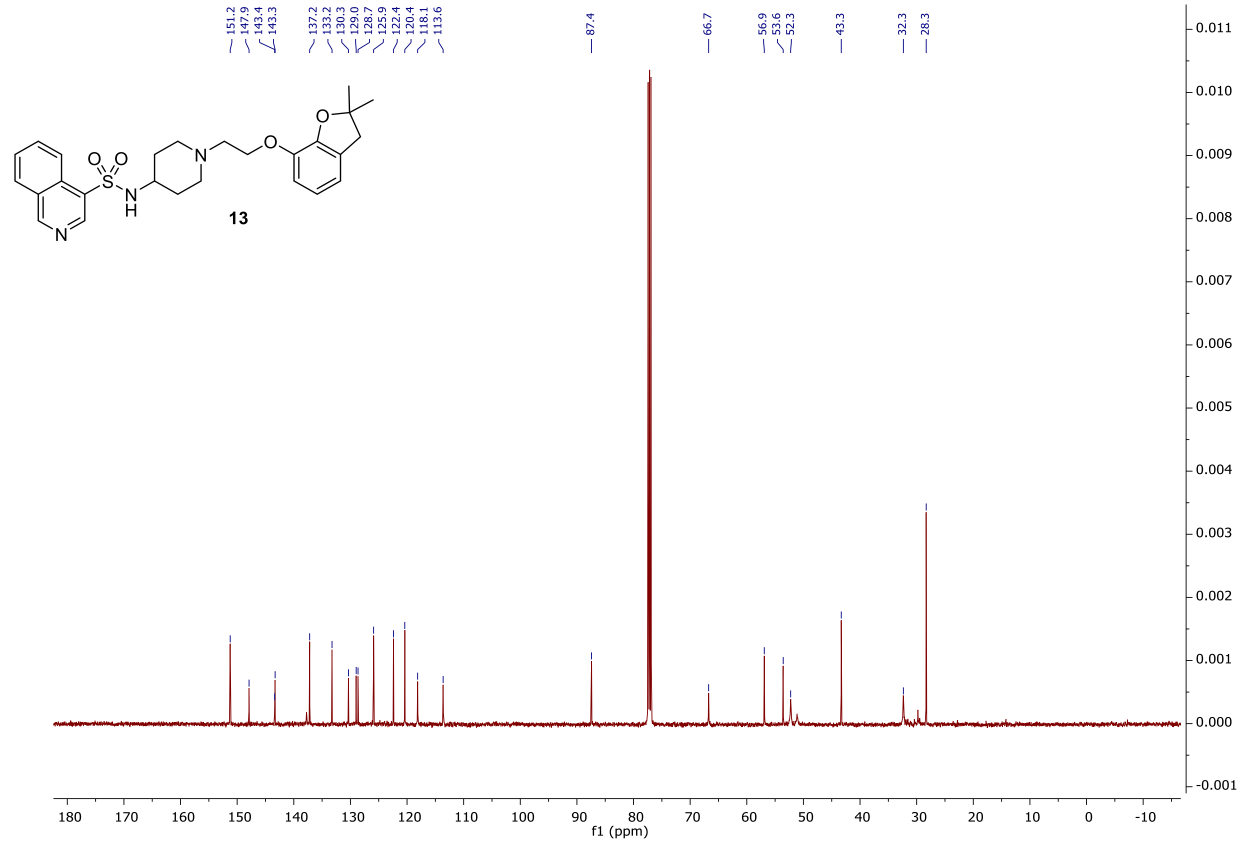


**Figure S36.** $^{13}$C-NMR spectra (125 MHz, $CDCl_3$) for 4-isoquinoline-*N*-{1-[2-(2,2-dimethyl-2,3-dihydrobenzofuran-7-yloxy)ethyl]piperidin-4-yl}sulfonamide (**13**).

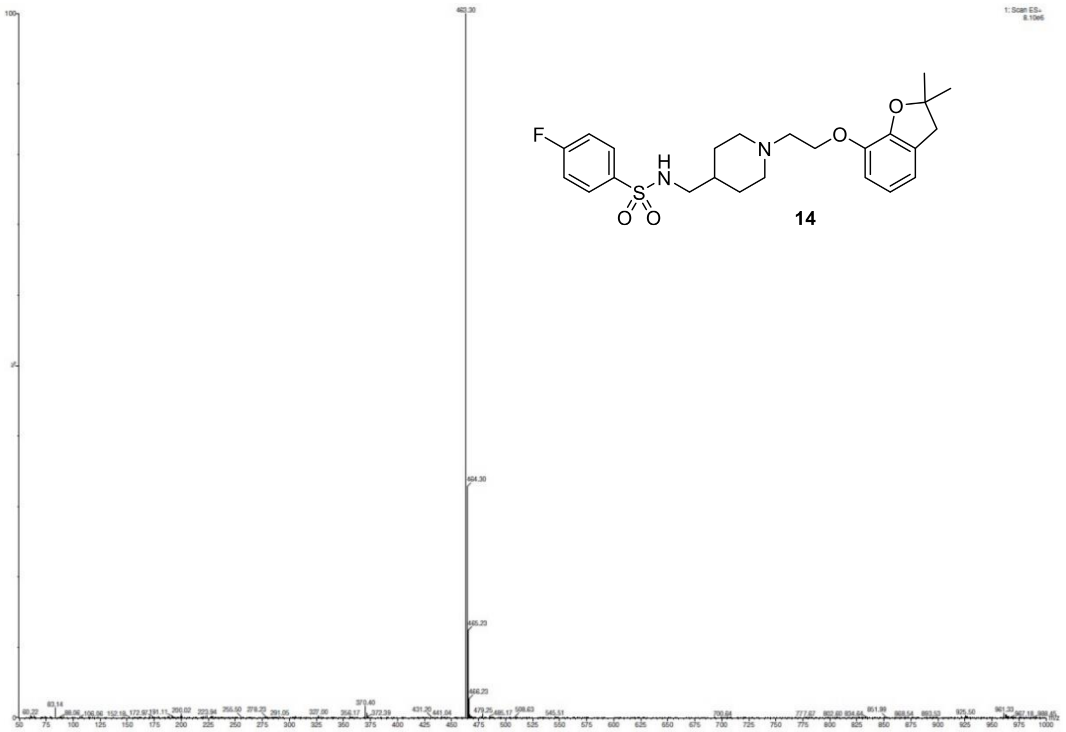

**Figure S37.** MS spectra for 4-fluoro-*N*-({1-[2-(2,2-dimethyl-2,3-dihydrobenzofuran-7-yloxy)ethyl]piperidin-4-yl}methyl)benzenesulfonamide (**14**).

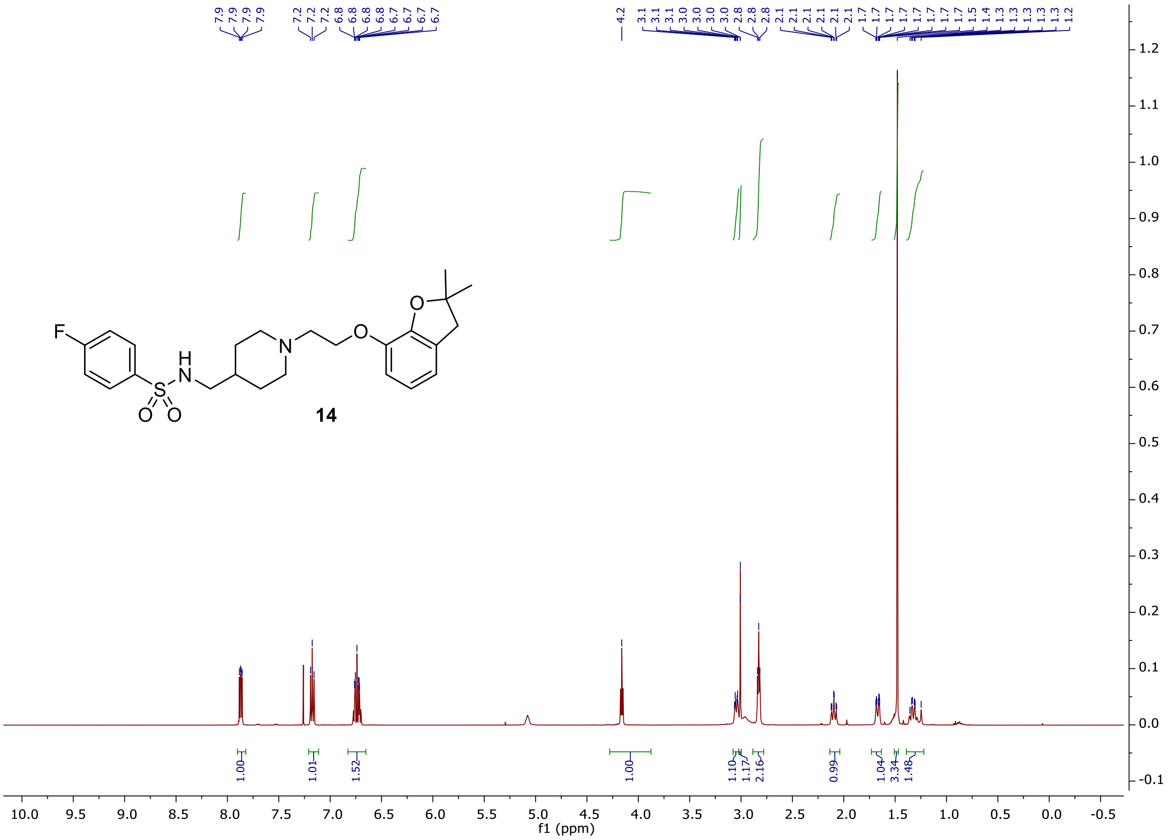


**Figure S38.** $^{1}$H-NMR spectra (500 MHz, $CDCl_3$) for 4-fluoro-*N*-({1-[2-(2,2-dimethyl-2,3-dihydrobenzofuran-7-yloxy)ethyl]piperidin-4-yl}methyl)benzenesulfonamide (**14**).

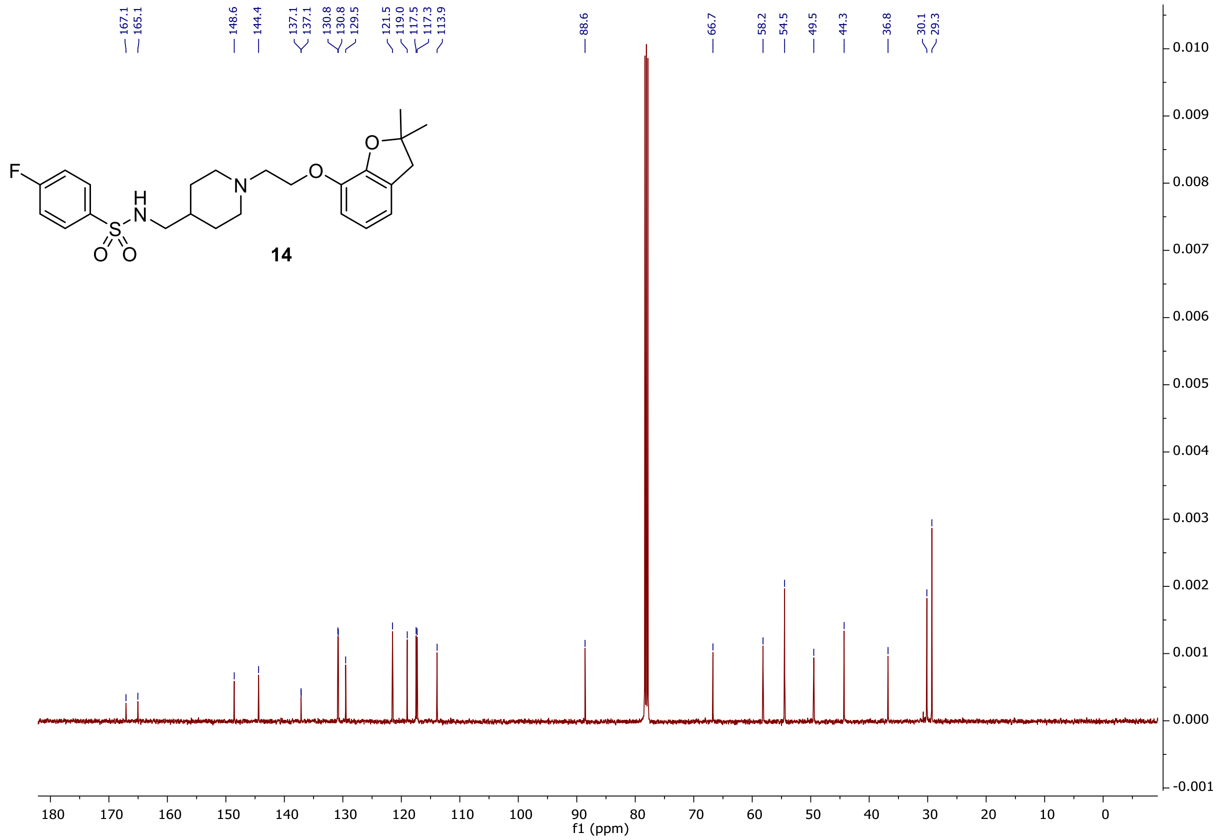


**Figure S39.** $^{13}C$-NMR spectra (125 MHz, $CDCl_3$) for 4-fluoro-*N*-({1-[2-(2,2-dimethyl-2,3-dihydrobenzofuran-7-yloxy)ethyl]piperidin-4-yl}methyl)benzenesulfonamide (**14**).

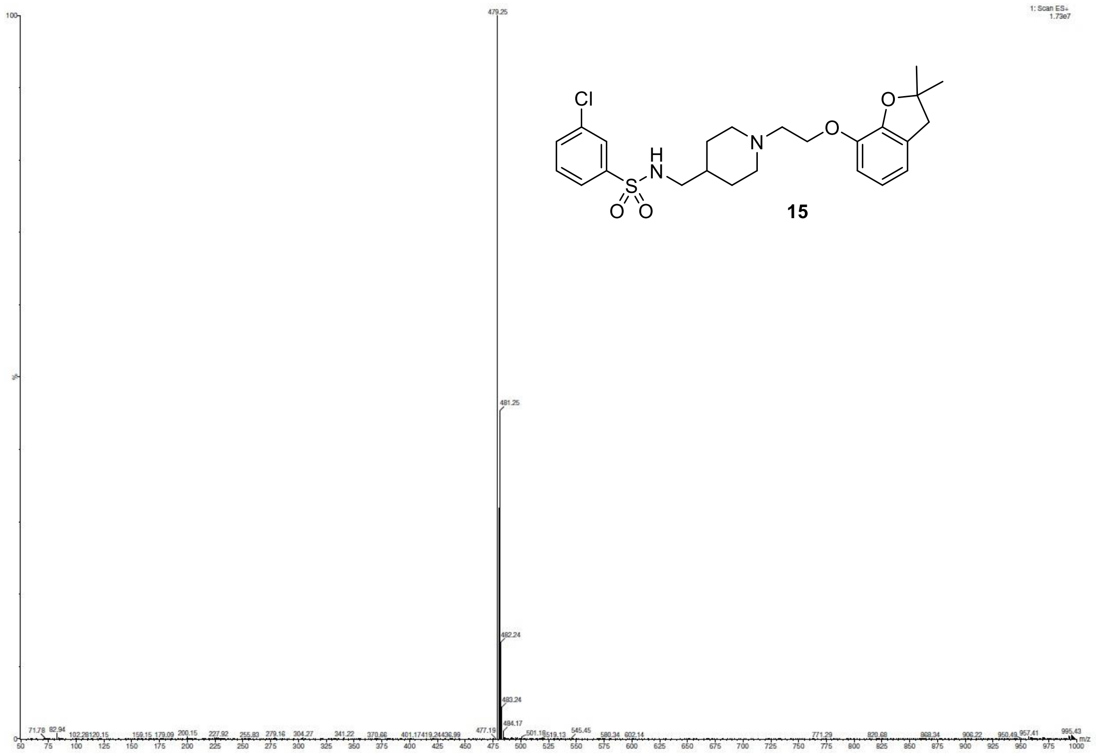

**Figure S40.** MS spectra for 3-chloro-*N*-({1-[2-(2,2-dimethyl-2,3-dihydrobenzofuran-7-yloxy)ethyl]piperidin-4-yl}methyl)benzenesulfonamide (15).

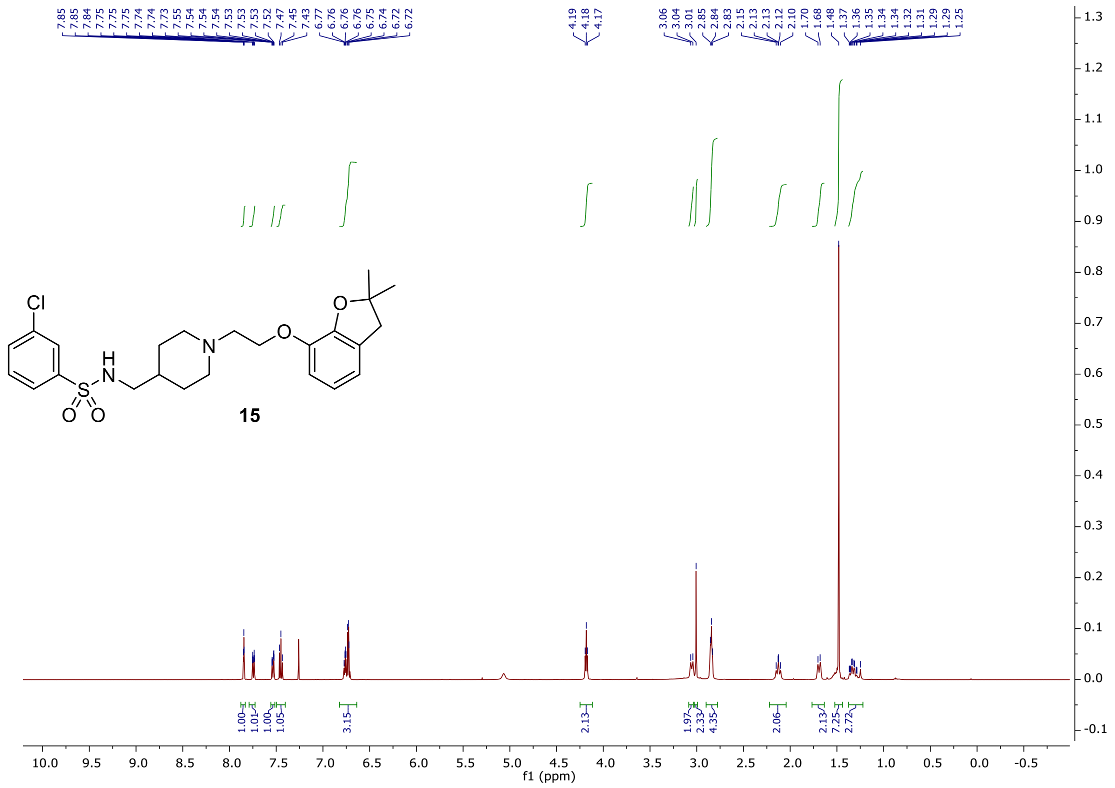

**Figure S41.** $^1$H-NMR spectra (500 MHz, $CDCl_3$) for 3-chloro-*N*-({1-[2-(2,2-dimethyl-2,3-dihydrobenzofuran-7-yloxy)ethyl]piperidin-4-yl}methyl)benzenesulfonamide (**15**).

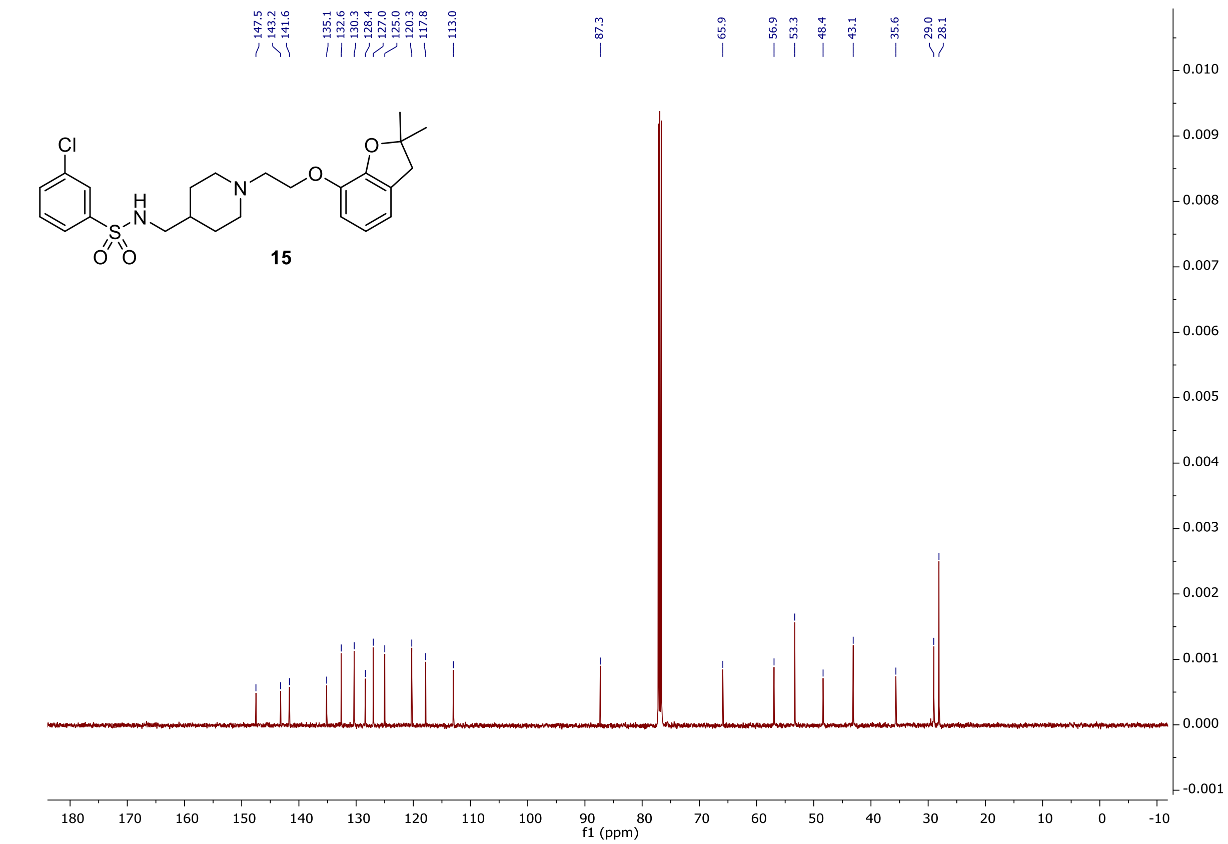


**Figure S42.** $^{13}$C-NMR spectra (125 MHz, $CDCl_3$) for 3-chloro-*N*-({1-[2-(2,2-dimethyl-2,3-dihydrobenzofuran-7-yloxy)ethyl]piperidin-4-yl}methyl)benzenesulfonamide (**15**).

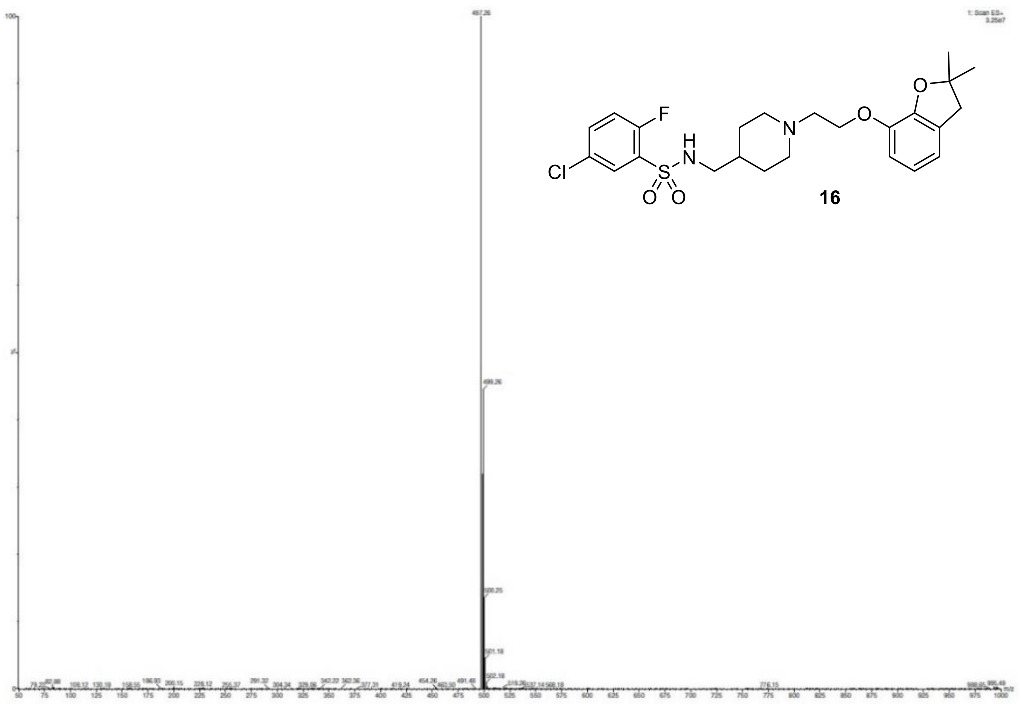


**Figure S43.** MS spectra for 5-chloro-2-fluoro-*N*-({1-[2-(2,2-dimethyl-2,3-dihydrobenzofuran-7-yloxy)ethyl]piperidin-4-yl}methyl)benzenesulfonamide (16).

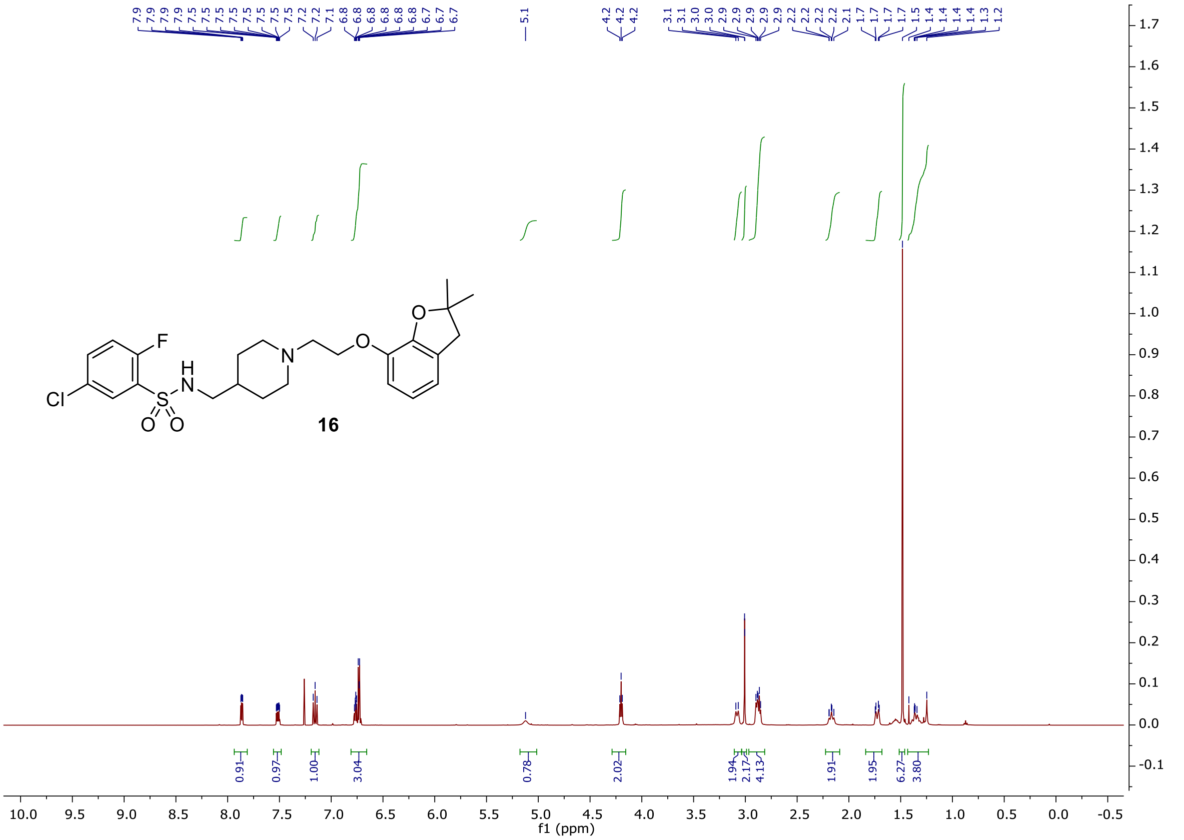


**Figure S44.** $^{1}$H-NMR spectra (500 MHz, $CDCl_3$) for 5-chloro-2-fluoro-*N*-({1-[2-(2,2-dimethyl-2,3-dihydrobenzofuran-7-yloxy)ethyl]piperidin-4-yl}methyl)benzenesulfonamide (**16**).

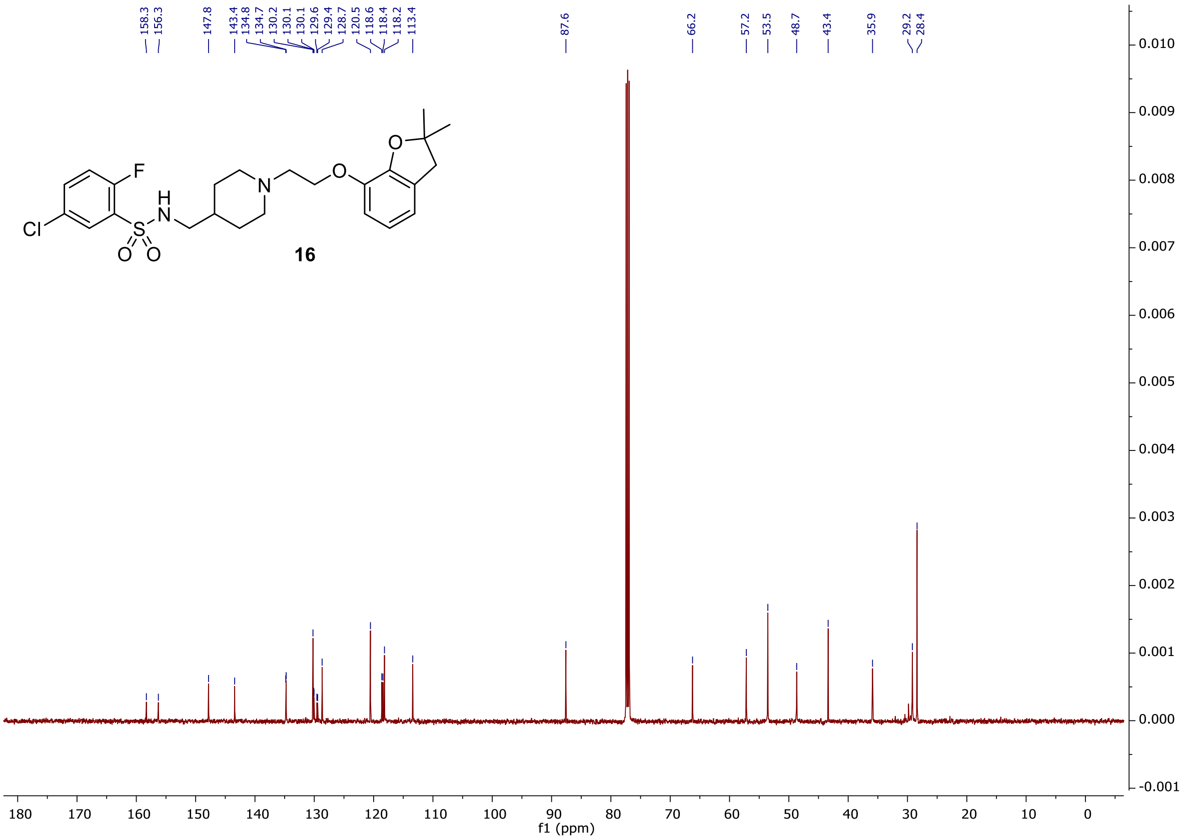


**Figure S45.** $^{13}$C-NMR spectra (125 MHz, $CDCl_3$) for 5-chloro-2-fluoro-*N*-({1-[2-(2,2-dimethyl-2,3-dihydrobenzofuran-7-yloxy)ethyl]piperidin-4-yl}methyl)benzenesulfonamide (**16**).

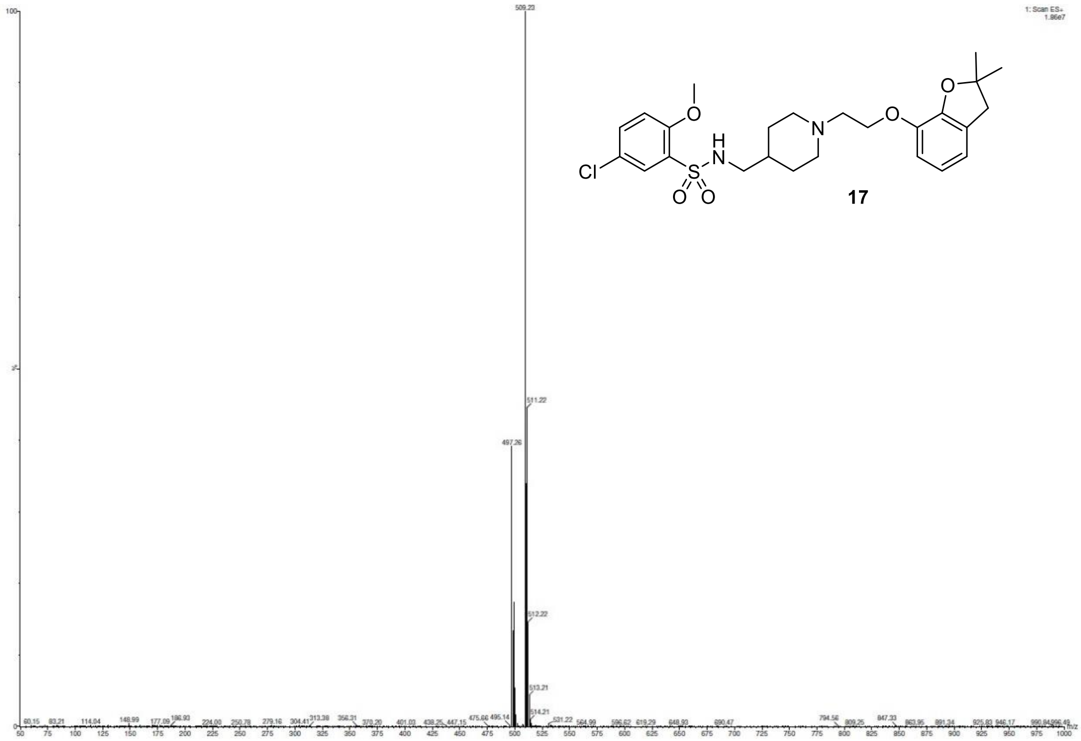


**Figure S46.** MS spectra for 5-chloro-2-methoxy-*N*-({1-[2-(2,2-dimethyl-2,3-dihydrobenzofuran-7-yloxy)ethyl]piperidin-4-yl}methyl)benzenesulfonamide (**17**).

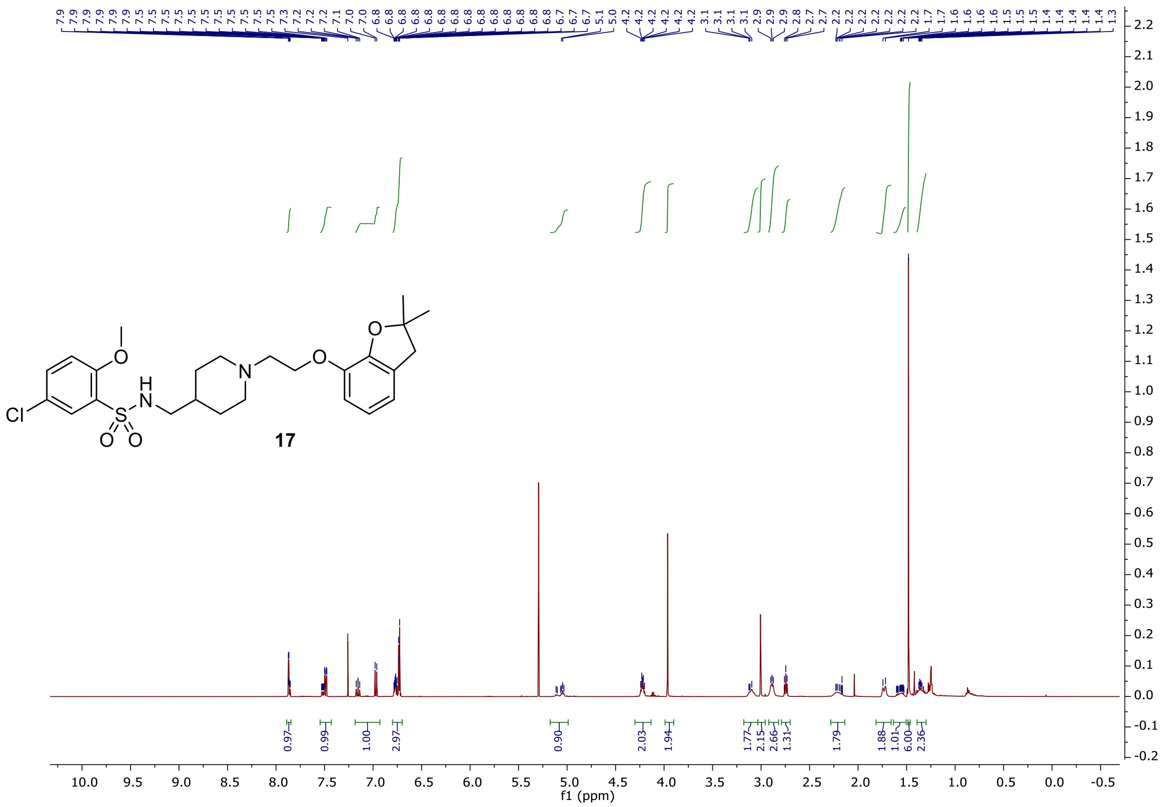


**Figure S47.** $^{1}$H-NMR spectra (500 MHz, $CDCl_3$) for 5-chloro-2-methoxy-*N*-({1-[2-(2,2-dimethyl-2,3-dihydrobenzofuran-7-yloxy)ethyl]piperidin-4-yl}methyl)benzenesulfonamide (**17**).

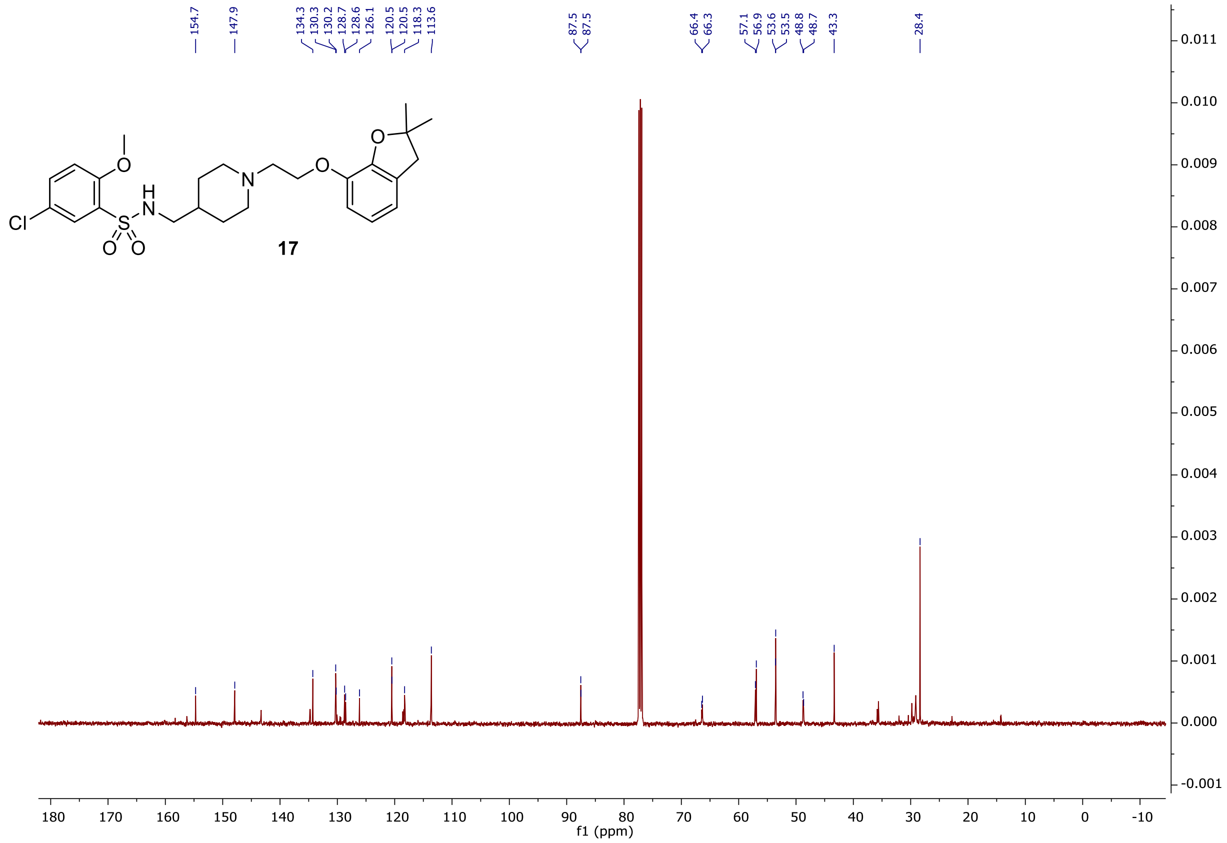


**Figure S48.** $^{13}$C-NMR spectra (125 MHz, $CDCl_3$) for 5-chloro-2-methoxy-*N*-({1-[2-(2,2-dimethyl-2,3-dihydrobenzofuran-7-yloxy)ethyl]piperidin-4-yl}methyl)benzenesulfonamide (**17**).

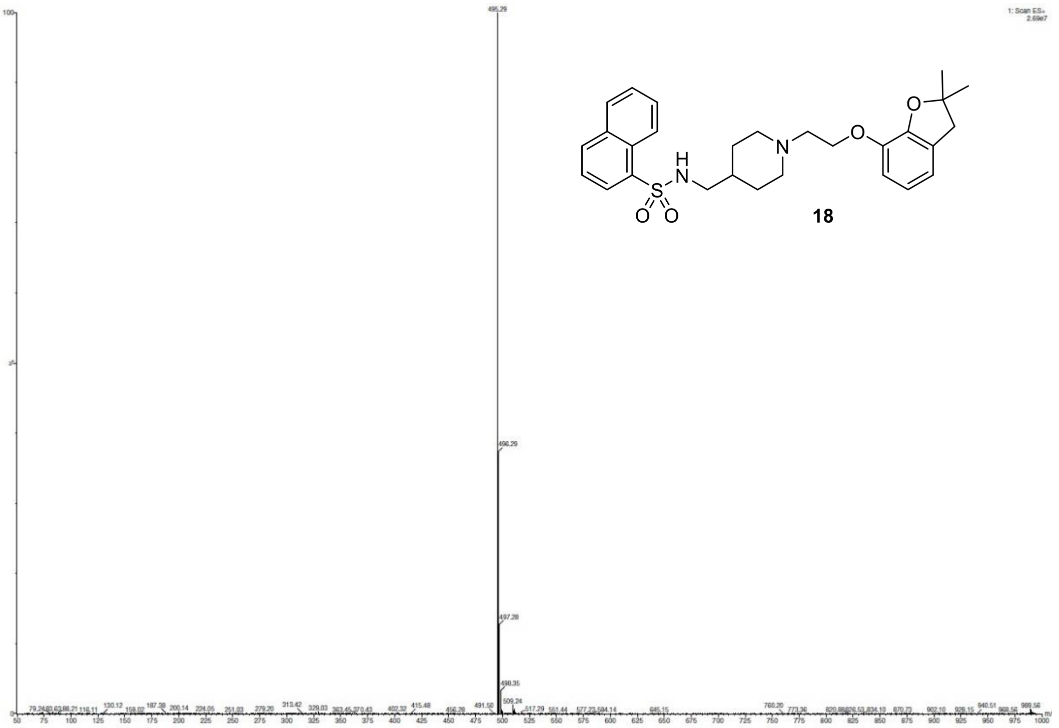


**Figure S49.** MS spectra for 1-naphthalene-*N*-({1-[2-(2,2-dimethyl-2,3-dihydrobenzofuran-7-yloxy)ethyl]piperidin-4-yl}methyl)sulfonamide (**18**).

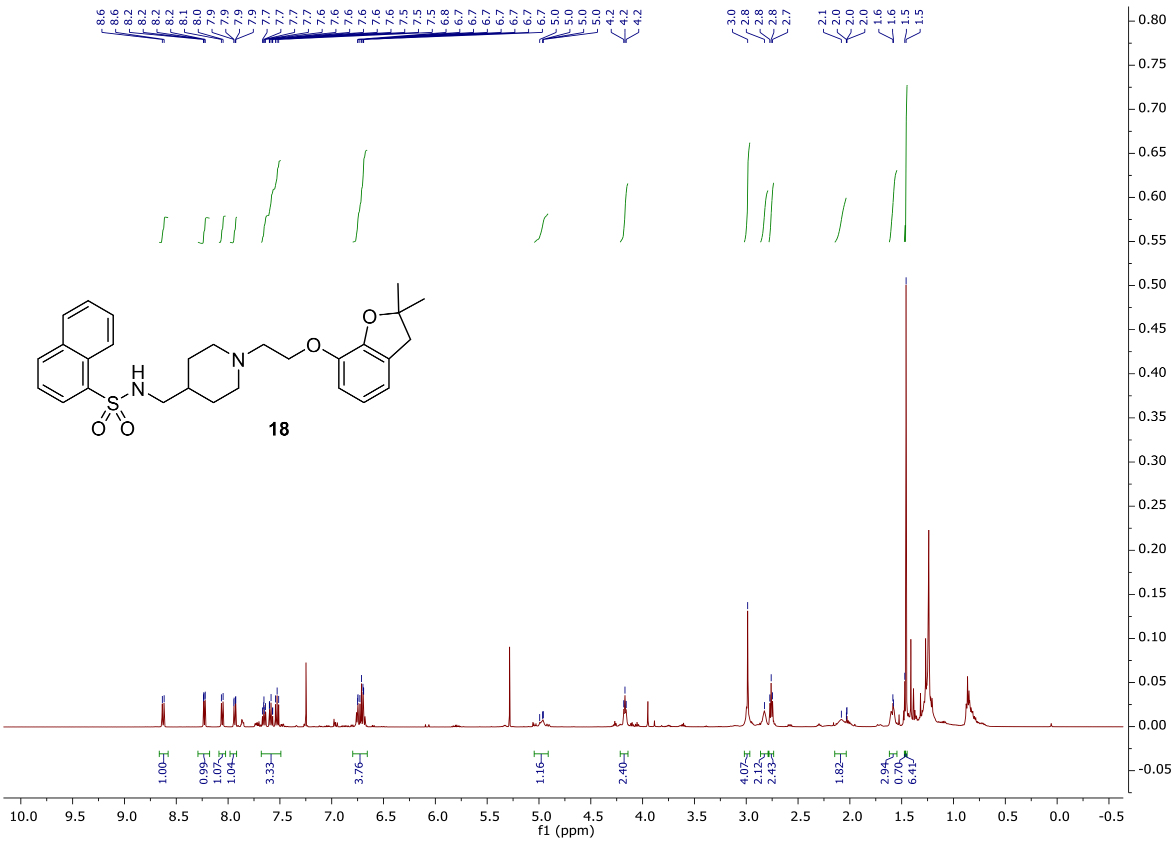


**Figure S50.** $^{1}$H-NMR spectra (500 MHz, $CDCl_3$) for 1-naphthalene-*N*-({1-[2-(2,2-dimethyl-2,3-dihydrobenzofuran-7-yloxy)ethyl]piperidin-4-yl}methyl)sulfonamide (**18**).

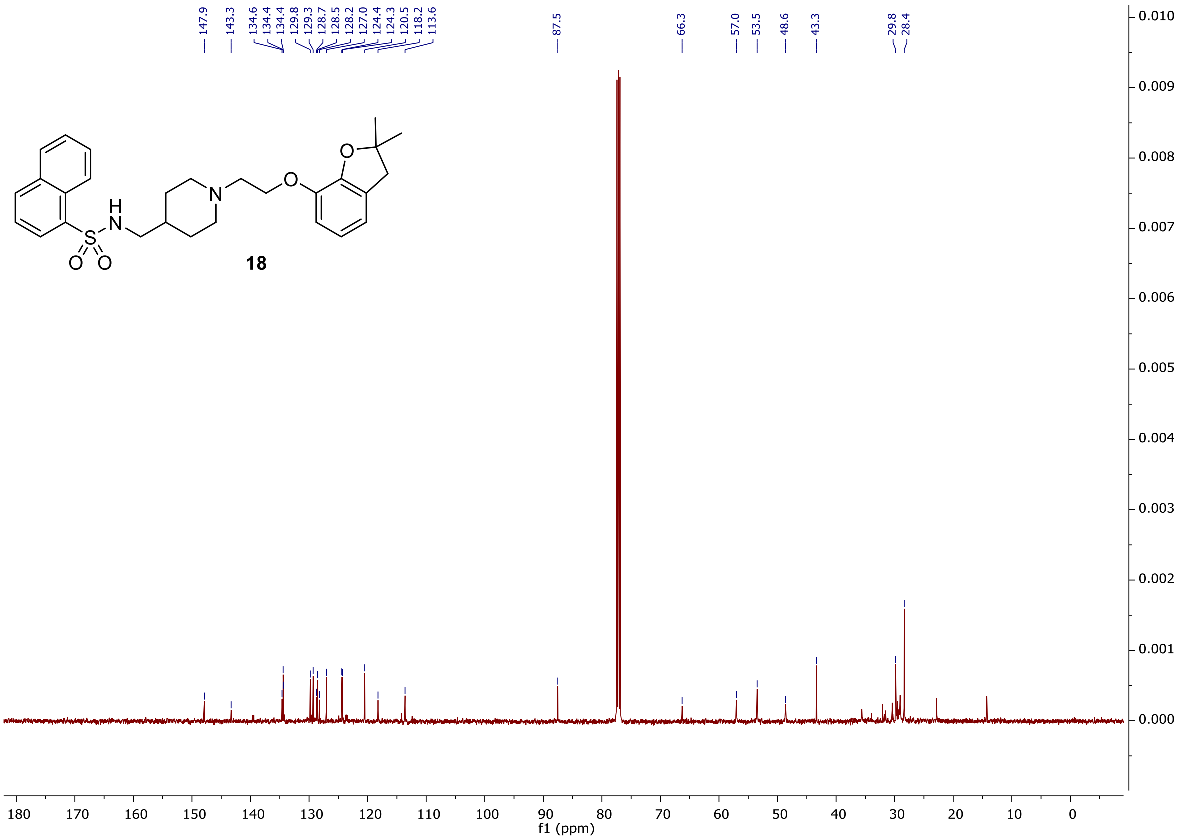


**Figure S51.** $^{13}$C-NMR spectra (125 MHz, $CDCl_3$) for 1-naphthalene-*N*-({1-[2-(2,2-dimethyl-2,3-dihydrobenzofuran-7-yloxy)ethyl]piperidin-4-yl}methyl)sulfonamide (**18**).

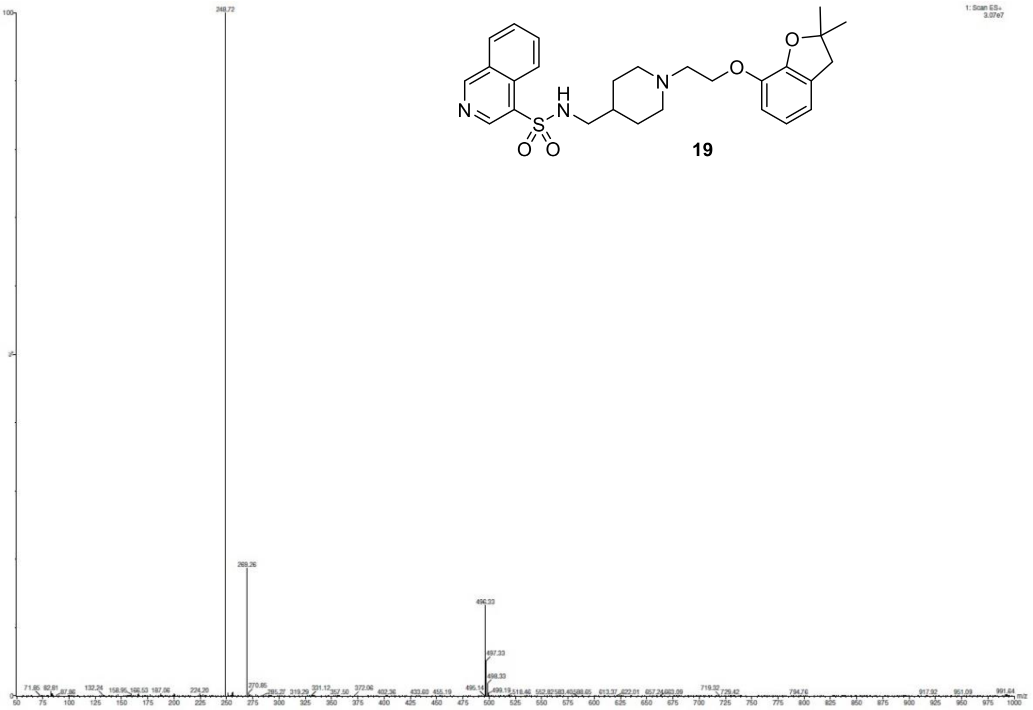


**Figure S52.** MS spectra for 4-isoquinoline-*N*-({1-[2-(2,2-dimethyl-2,3-dihydrobenzofuran-7-yloxy)ethyl]piperidin-4-yl}methyl)sulfonamide (**19**).

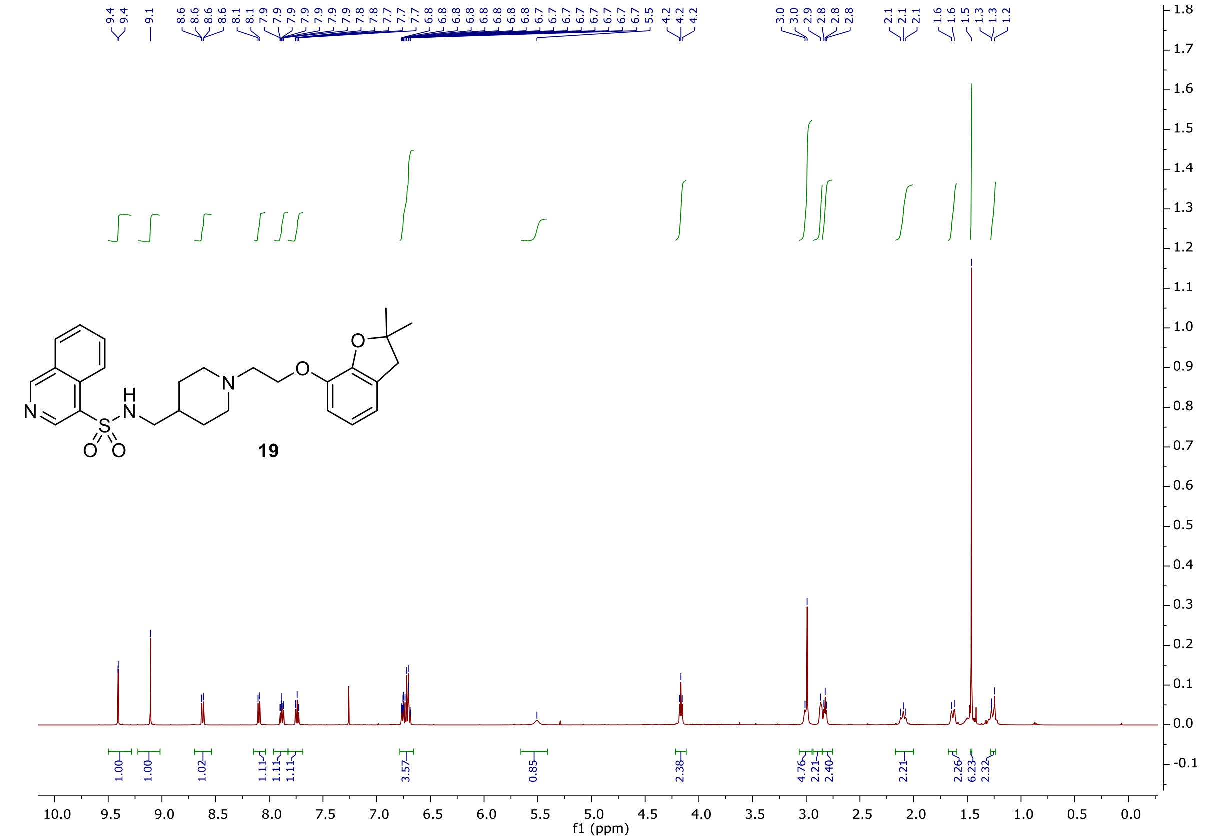


**Figure S53.** $^{1}$H-NMR spectra (500 MHz, $CDCl_3$) for 4-isoquinoline-*N*-({1-[2-(2,2-dimethyl-2,3-dihydrobenzofuran-7-yloxy)ethyl]piperidin-4-yl}methyl)sulfonamide (**19**).

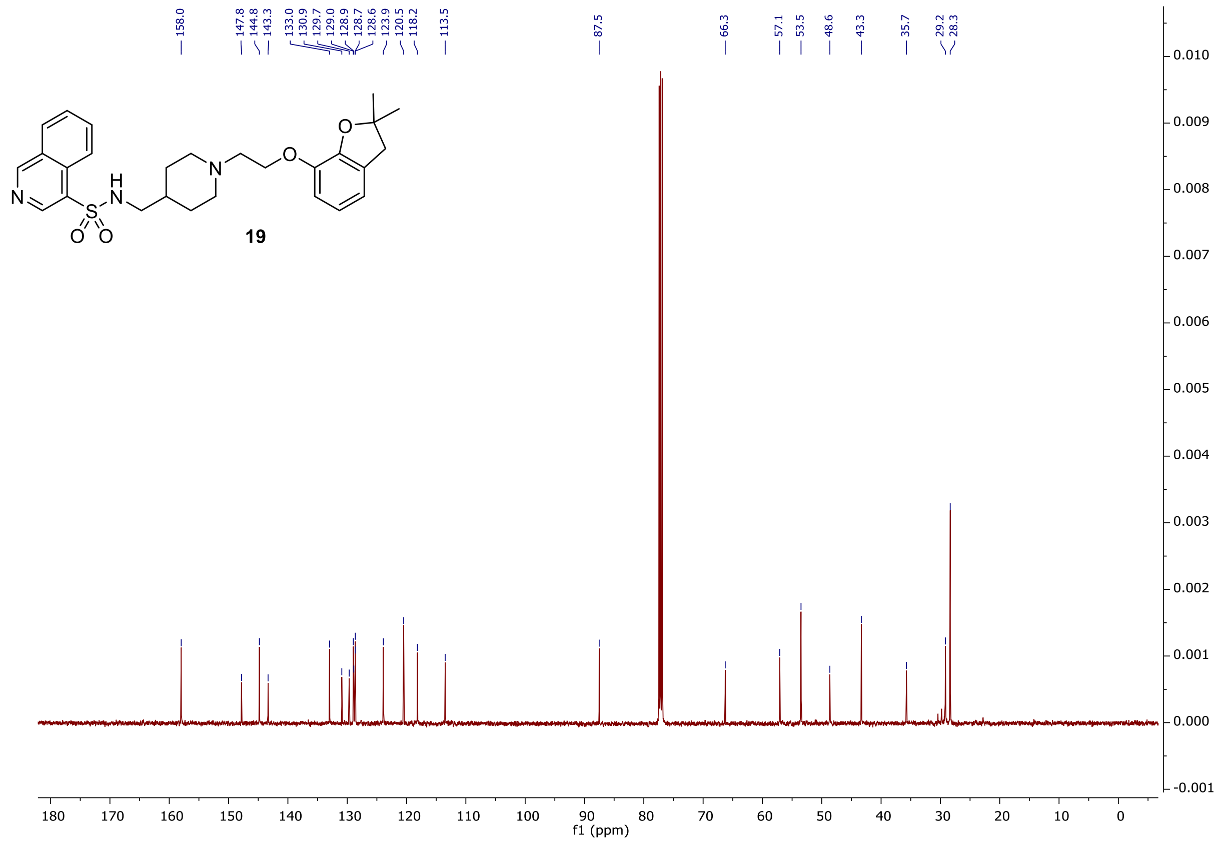


**Figure S54.** $^{13}$C-NMR spectra (125 MHz, $CDCl_3$) for 4-isoquinoline-*N*-({1-[2-(2,2-dimethyl-2,3-dihydrobenzofuran-7-yloxy)ethyl]piperidin-4-yl}methyl)sulfonamide (**19**).
